# A plant NLR receptor activates auxin signaling through Aux/IAAs-ARF19 and YUC8-TIR1/AFBs to promote callose-mediated antiviral defense

**DOI:** 10.1126/sciadv.aea2275

**Published:** 2025-12-17

**Authors:** Tongqing Yang, Ruizhen Zhao, Ruoxin Mei, Hongmin Cui, Zixuan Ding, Yanan Wen, Qian Wu, Zhengqiang Chen, Shen Huang, Chunli Wang, Lu Hong, Wenyu Zuo, Zhongkai Zhang, Min Zhu, Leiyun Yang, Zhengguang Zhang, Suomeng Dong, Yi Xu, Xiaorong Tao

**Affiliations:** ^1^ State Key Laboratory of Agricultural and Forestry Biosecurity, Nanjing Agricultural University, Nanjing 210095, China.; ^2^ Key Laboratory of Plant Immunity, Department of Plant Pathology, Nanjing Agricultural University, Nanjing 210095, China.; ^3^ Key Laboratory of Integrated Management of Crop Disease and Pests, Ministry of Education, Nanjing Agricultural University, Nanjing 210095, China.; ^4^ Inner Mongolia Potato Engineering and Technology Research Center, Inner Mongolia University, Hohhot 010021, China.; ^5^ Key Laboratory of Biohazard Monitoring, Green Prevention and Control for Artificial Grassland, Ministry of Agriculture and Rural Affairs, Institute of Grassland Research of Chinese Academy of Agricultural Sciences, Hohhot 010010, China.; ^6^ Yunnan Provincial Key Laboratory of Agri-Biotechnology, Institute of Biotechnology and Genetic Resources, Yunnan Academy of Agricultural Sciences, Kunming 650223, China.

## Abstract

Phytohormone signaling pathways are crucial for defense against pathogens mediated by pattern recognition receptors and nucleotide-binding leucine-rich repeat (NLR) immune receptors. The induction of auxin signaling by immune receptors for antiviral immunity is poorly understood despite its notable role in plant defense against viral pathogens. Here, we report that plant NLR Sw-5b initiates and amplifies auxin signaling through auxin/indole-3-acetic acid (Aux/IAAs)–ARF19 and YUC8–transport inhibitor response1/auxin-signaling F-box (TIR1/AFB) modules to promote callose-mediated antiviral defense. Upon recognizing viral effector, Sw-5b associates with and relieves repressors Aux/IAAs on transcription factor ARF19. ARF19 then activates callose synthase gene *GSL5/8* to deposit callose at plasmodesmata, inhibiting viral cell-to-cell spread. Meanwhile, ARF19 activates auxin biosynthesis gene *YUC8* to boost auxin production; this further amplifies callose deposition signaling via TIR1/AFB receptors and Aux/IAAs-ARF19–glucan synthase-like (GSL) module, thereby restricting the virus in localized cell death. Our findings provide valuable insights into the mechanism by which plant immune receptors induce phytohormone signaling pathways to combat pathogens.

## Teaser

Plant NLR receptor uses auxin central modules to activate its signaling pathway, promoting callose-mediated antiviral immunity.

## INTRODUCTION

Plants have evolved a two-layered immune system to combat pathogen invasions (*1*–*3*). The first layer, pattern-triggered immunity (PTI), relies on pattern recognition receptors (PRRs) that detect conserved pathogen/microbe-associated molecular patterns (PAMPs/MAMPs) to activate basal defense responses (*4*, *5*). To combat viral pathogen infections, plants have also evolved an RNA interference (RNAi) system that detects viral double-stranded RNA (dsRNA)—a viral PAMP—to activate basal antiviral immunity (*6*, *7*). However, pathogens counteract PTI by evolving effector proteins to suppress host immunity and establish infection—a strategy termed virulence (*8*–*10*). In response, plants have evolved the second layer of defense, effector-triggered immunity (ETI), which is mediated by intracellular nucleotide-binding leucine-rich repeat (NLR) immune receptors, to trigger a long-lasting and strong plant immunity to eliminate invading pathogens (*1*–*3*). NLR immune receptors represent the largest class of resistance genes in plants and are characterized by three major domains: an N-terminal Toll/interleukin-1 receptor (TIR) or coiled-coil (CC) domain, a central nucleotide-binding adaptor shared by APAF-1, certain R proteins and CED-4 (NB-ARC) domain, and a C-terminal LRR domain. They recognize specific pathogen effectors directly or indirectly to trigger a robust plant immunity. ETI is typically associated with a series of defense response, including calcium ion influx, reactive oxygen species burst, defense hormones production, callose deposition, and the hypersensitive response (HR), which leads to localized cell death to prevent the spread of pathogens (*1*–*3*, *11*). Despite this, the underlying mechanism by which NLR receptors activate defense hormone signaling and callose deposition remains elusive.

As the first found phytohormone, auxin plays a pivotal role in plant growth and development (*12*). The nuclear auxin signaling cascade is initiated when auxin [mainly indole-3-acetic acid (IAA) in plants] binds to its cognate receptors, transport inhibitor response1/auxin-signaling F-box (TIR1/AFB) proteins. This ligand-receptor interaction recruits auxin/IAA (Aux/IAA) transcriptional repressors, triggering their ubiquitination and subsequent proteasomal degradation through the TIR1/AFB containing Skp1-Cul1-F-box protein (SCF) ubiquitin ligase complex. The degradation of these repressors releases auxin response factors (ARFs), enabling their dimerization and binding to auxin-responsive promoter elements, ultimately leading to ARF-mediated transcriptional reprogramming of auxin response genes (*13*–*15*). As an essential phytohormone for plants, auxin has also been widely involved in host immune responses against pathogens. Activation of auxin signaling results in reduced disease susceptibility to necrotrophic fungi (*16*–*18*), oomycetes (*19*), and viruses (*20*–*24*). In rice, disruption of auxin signaling by inhibiting the expression of auxin receptor TIR1/AFBs results in enhanced susceptibility to *Rhizoctonia solani* (*18*). Knocking out *OsARF17* leads to reduced resistance against several RNA viruses, and loss of function of *OsARF12* or *OsARF16* exhibit reduced resistance to rice dwarf virus (RDV) (*20*, *24*). Also, pathogens have evolved effectors that target different components of the auxin signaling pathway to promote their infection (*21*–*26*). A recent study reported that PRR-mediated PTI can activate auxin signaling pathways (*27*). However, it remains unclear whether plant NLR receptors can activate auxin signaling upon recognition of effectors. Furthermore, the biological importance of auxin pathway activation against pathogens remains poorly characterized.

Callose (beta-1,3 glucan) deposition is a well-known immune response in both PTI and ETI (*28*). During pathogen infection, plants induce callose synthesis and accumulation to reinforce cell walls, thereby preventing pathogen penetration and spread (*29*). Plasmodesmata (PD) are plasma membrane–lined channels that interconnect plant cells, allowing for the intercellular transport of various molecules across tissues and organs (*30*). Viral movement proteins (MPs) can increase the size exclusion limit of PD, facilitating viral cell-to-cell movement (*31*). However, callose deposition at PD can effectively restrict viral cell-to-cell movement by physically occluding these intercellular channels, thereby impeding viral spread between plant cells (*32*, *33*). The regulation of PD connectivity is primarily controlled by callose deposition at the neck regions of PD (*34*). Callose synthesis is mainly regulated by the glucan synthase–like (GSL) enzymes, which play crucial roles in modulating symplastic transport (*34*). In *Arabidopsis*, 12 GSL family members have been identified, with GSL5, GSL8, and GSL12 shown to be localized at PD (*35*). The *Arabidopsis gsl8* mutants exhibit reduced callose accumulation at the cell plate and PD, leading to abnormal stomatal distribution in epidermal leaf cells (*36*). GSL8 interacts with GSL10 to regulate PD permeability and male gametophyte development (*37*, *38*). In response to H_2_O_2_ treatment or wounding, the *gsl4* mutants fail to induce callose accumulation (*39*).

Tomato spotted wilt orthotospovirus (TSWV) is one of the most important plant viruses, with a broad host range encompassing over 1000 plant species including economically important crops such as tomatoes, peppers, peanuts, lettuce, and numerous ornamental plants, causing an estimated annual economic loss of more than one billion US dollars worldwide (*40*). *Sw-5b* is one of the most effective resistance genes that confers the resistance against TSWV and has been extensively used in tomato resistance breeding. As a CC-type NLR (CNL) protein that harbors an additional N-terminal *Solanaceae* domain (SD), Sw-5b recognizes the TSWV movement protein NSm (nonstructural protein from viral medium RNA segment) to induce antiviral immune responses (*41*–*43*). Our previous investigations have established that the SD mediates nuclear localization of Sw-5b, a prerequisite for conferring resistance against TSWV intercellular spread (*44*). However, the molecular mechanism by which nuclear-localized Sw-5b orchestrates immune responses to restrict TSWV intercellular movement remain unexplored.

In this study, we report that upon NSm recognition, Sw-5b NLR initiates and boosts auxin signaling via the Aux/IAAs-ARF19 and the YUC8-IAA-TIR1/AFBs modules to induce a robust callose deposition at PD against viral infection. We demonstrate that upon perception of the viral effector NSm, Sw-5b triggers the transcriptional activation of *GSL* genes 5/8/10 (*GSL5/8/10*), leading to plasmodesmal callose deposition and subsequent impediment of viral intercellular movement. ARF19 directly binds to *GSL8* promoter to promote callose production, a process negatively regulated by the transcriptional repressors IAA13/27/29/33/35. Upon activation by NSm, Sw-5b interacts with these Aux/IAAs via its SD, disrupting their association with ARF19 and thereby releasing ARF19 to activate the expression of downstream genes. Furthermore, ARF19 binds to the promoter of *YUC8* and up-regulates *YUC8* expression, which augment IAA biosynthesis. The elevated IAA activates auxin receptors TIR1/AFBs to degrade Aux/IAA transcriptional repressors, establishing a positive feedback loop that further amplifies auxin signaling, thereby inducing a strong callose deposition in cell wall. This coordinated regulatory mechanism reinforces PD closure, thereby effectively restricting viral spread through localized cell death. In addition, another SD-containing NLR R8 interacts with IAA13/27 and similarly activates auxin signaling and callose deposition upon recognition of the effector AVR8.

## RESULTS

### Sw-5b inhibits TSWV intercellular movement by up-regulating GSL5/8/10-mediated callose deposition in *Nicotiana benthamiana*

**Fig. 1.**
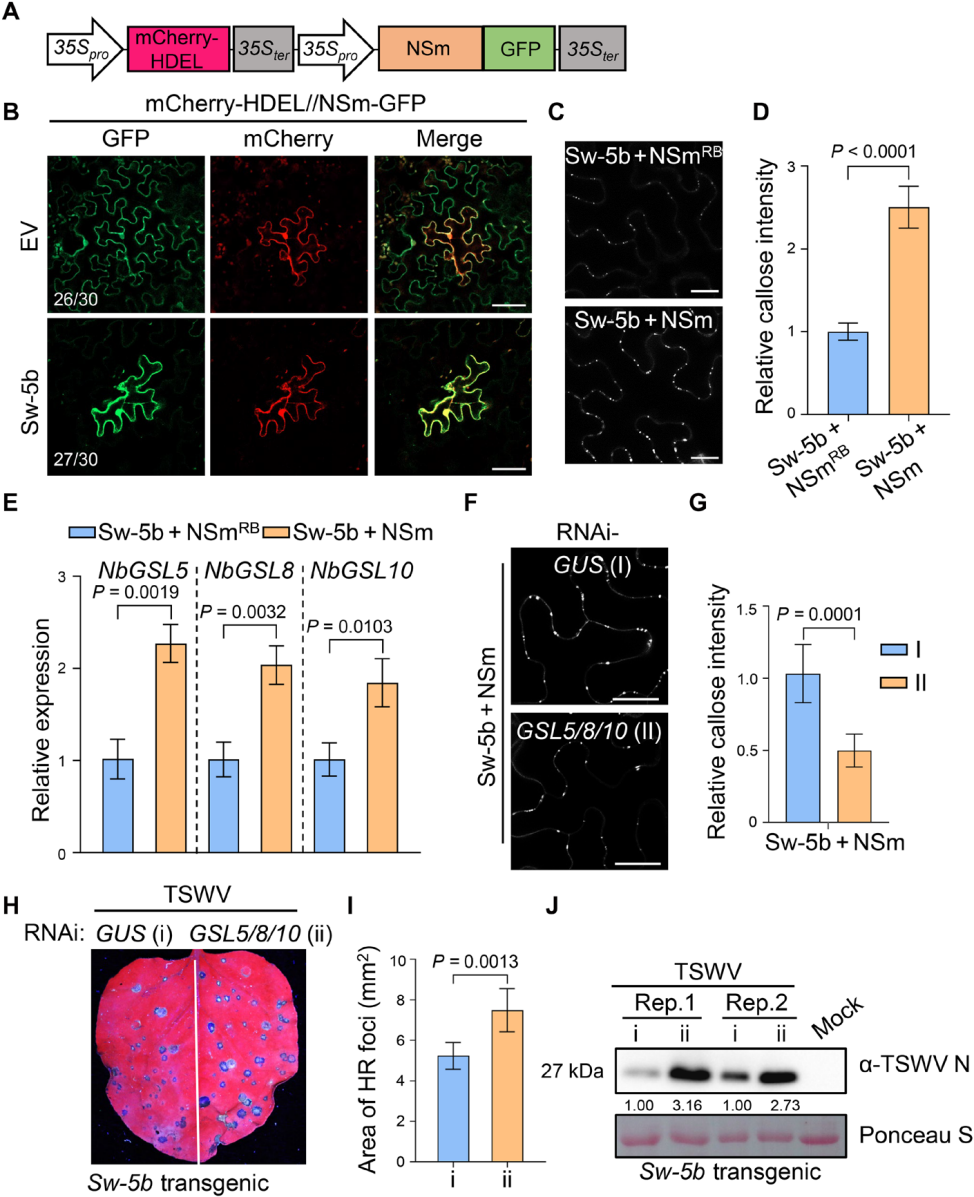
Callose synthetases GSL5/8/10 positively regulate *Sw-5b*–mediated inhibition of TSWV movement in *N. benthamiana*. (**A**) Schematic diagram of the binary construct coexpressing NSm-GFP and mCherry-HDEL. mCherry-HDEL served as the unmovable endoplasmic reticulum marker. (**B**) Fluorescent images of NSm-GFP coexpressed with Sw-5b or EV in *N. benthamiana* leaf cells. (**C**) Aniline blue staining of callose in leaf epidermis expressing Sw-5b with NSm or NSm^RB^. (**D**) Quantification of callose intensity in (C). Values are means ± standard deviation of six biologically independent samples. (**E**) Relative expression levels of *NbGSL5*, *NbGSL8*, and *NbGSL10* in leaves expressing Sw-5b together with NSm or NSm^RB^ by quantitative real-time polymerase chain reaction (qRT-PCR). *Nbactin* was used as the internal reference gene. Values represent means ± standard deviation of three biological replicates. (**F**) Callose fluorescence in *GSL5/8/10*- or *GUS*-silenced leaves coexpressing Sw-5b and NSm. (**G**) Relative intensity of callose in (F). Values represent means ± standard deviation of six biologically independent samples. (**H**) HR foci in *GSL5/8/10*- or *GUS*-silenced half-leaves from Sw-5b–transgenic plants inoculated with TSWV. Photos were taken at 3 dpi under an ultraviolet (UV) light. (**I**) Quantification of mean areas of HR foci in (H). Values are means ± standard deviation of six biologically independent leaf samples. (**J**) Immunoblotting analysis of TSWV accumulation in leaf samples from (H). Ponceau staining shows protein loading. Protein levels were determined using ImageJ software. Images in (B), (C), (E), and (F) were taken at 22 hpi. Statistical analysis in (D), (E), (G), and (I) was performed by two-tailed Student’s *t* test. Scale bars, 20 μm.

TSWV encodes an MP NSm that typically promotes cell-to-cell spread and is recognized by Sw-5b to trigger antiviral immunity (*41*, *45*). Our previous investigations have demonstrated that upon recognition of NSm in the cytoplasm, the immune receptor Sw-5b initiates immune responses in the nucleus to restrict viral intercellular movement (*44*). To further investigate the underlying mechanisms, we transiently coexpressed Sw-5b or empty vector (EV) with the mCherry-HDEL//NSm–green fluorescent protein (GFP), a dual-fluorescence reporter system carrying TSWV MP NSm-GFP gene fusion and an endoplasmic reticulum restricted mCherry in the same construct (Fig. 1A) (*45*) in *N. benthamiana* leaves. Consistent with our prior findings, coexpression with Sw-5b resulted in the NSm-GFP (green fluorescence) with mCherry-HDEL (red fluorescence) confined to single cells, whereas in control samples, NSm-GFP spread from one cell to adjacent cells (Fig. 1B). To examine whether the plasmodesmal permeability is reduced upon Sw-5b activation, we applied 5(6)-carboxyfluorescein diacetate (CFDA) dye onto the *N. benthamiana* leaves coexpressing Sw-5b and NSm or Sw-5b and nonelicitor NSm^RB^ mutant (*41*). Confocal microscopy analysis showed that fluorescent area of CF dye is significantly smaller in leaf cells expressing Sw-5b and NSm compared to those expressing Sw-5b and NSm^RB^ (fig. S1, A and B). To determine whether the reduced plasmodesmal permeability resulted from callose deposition, we applied aniline blue staining in treated leaves. As shown in Fig. 1 (C and D), callose intensity was significantly elevated in cells coexpressing Sw-5b and NSm compared to those coexpressing Sw-5b and NSm^RB^. Moreover, the callose signal was colocalized with the PD marker PDLP5–yellow fluorescent protein (YFP) (fig. S1C) (*46*). Collectively, these data indicate that upon NSm recognition, Sw-5b induces a robust callose deposition at PD.

To investigate how Sw-5b induces callose deposition, we conducted RNA sequencing (RNA-seq) analysis using *N*. *benthamiana* leaves coexpressing Sw-5b with NSm or EV. A total of 6929 differentially expressed genes (DEGs) were identified, including 3343 up-regulated and 3586 down-regulated genes (|log_2_ FC| > 1.5, *P* < 0.05) (table S1). Given that GSL family proteins are responsible for the callose biosynthesis (*47*), we focused on the expression profiles of *GSL* genes. Among seven identified *GSLs* in RNA-seq data, four genes were significantly up-regulated (fig. S2A), with *NbGSL5*, *NbGSL8*, and *NbGSL10* showing the highest fold increases upon Sw-5b activation (Fig. 1E). In addition, overexpression of NbGSL5, NbGSL8, or NbGSL10 each in *N. benthamiana* leaves significantly induced callose deposition at PD (fig. S2, B and C), suggesting that these GSLs are responsible for PD callose production. To investigate the role of NbGSL5/8/10 in TSWV infection, we expressed TSWV infectious clones [L_(+)opt_ + SR_(+)eGFP_ + M_(−)opt_] together with these GSLs or with EV. The results showed that overexpression of these three proteins reduced the number of green fluorescent cells infected by TSWV infectious clones (fig. S2, D and E) and the accumulation of the viral nucleocapsid protein (NP) (fig. S2F), indicating that all three GSLs play critical roles in resistance against TSWV.

Next, we attempted to simultaneously silence *NbGSL5/8/10* using tobacco rattle virus (TRV)–based virus-induced gene silencing (VIGS) in *N. benthamiana* plants. However, newly emerged leaves of silenced plants exhibited severe downward curling and failed to expand normally at the early stage, followed by stunted growth and abnormal floral organ development at the later stage (fig. S3), indicating their essential roles in plant growth and development. These phenotypic abnormalities prompted us to switch to knock down *NbGSL5/8/10* using a transient silencing system via dsRNA-induced RNAi (fig. S4A). We expressed RNAi: *GSL5/8/10* construct in the half leaves of *N. benthamiana* and treated with RNAi: *GUS* construct as a control in another half of the same leaves (fig. S4, B and C). After RNAi treatment for 12 hours, Sw-5b and NSm were transiently coexpressed, and 22 hours later, the level of callose was analyzed (fig. S4B). The results showed that callose accumulation at PD was significantly reduced in *GSL5/8/10*-silenced leaf tissues compared to controls (Fig. 1, F and G). We also coexpressed Sw-5b and mCherry-HDEL//NSm-GFP reporter in RNAi-treated leaves and observed the GFP signals under a confocal microscope (fig. S4B). As shown in fig. S4 (D and E), RNAi of *GSL5/8/10* notably weakened Sw-5b’s ability to inhibit intercellular movement of NSm. We also mechanically inoculated with TSWV in *Sw-5b*–transgenic *N. benthamiana* leaves after RNAi treatment (fig. S4B) and observed significantly enlarged HR lesions on *GSL5/8/10-*silenced half-leaves and increased accumulation of viral NP compared to the nonsilenced control leaf tissues (Fig. 1, H to J). Collectively, these results indicate that NbGSL5/8/10 induce plasmodesmal callose deposition and positively regulate *Sw-5b*–mediated defense against viral intercellular movement.

### Sw-5b interacts with transcription repressors IAA35

**Fig. 2.**
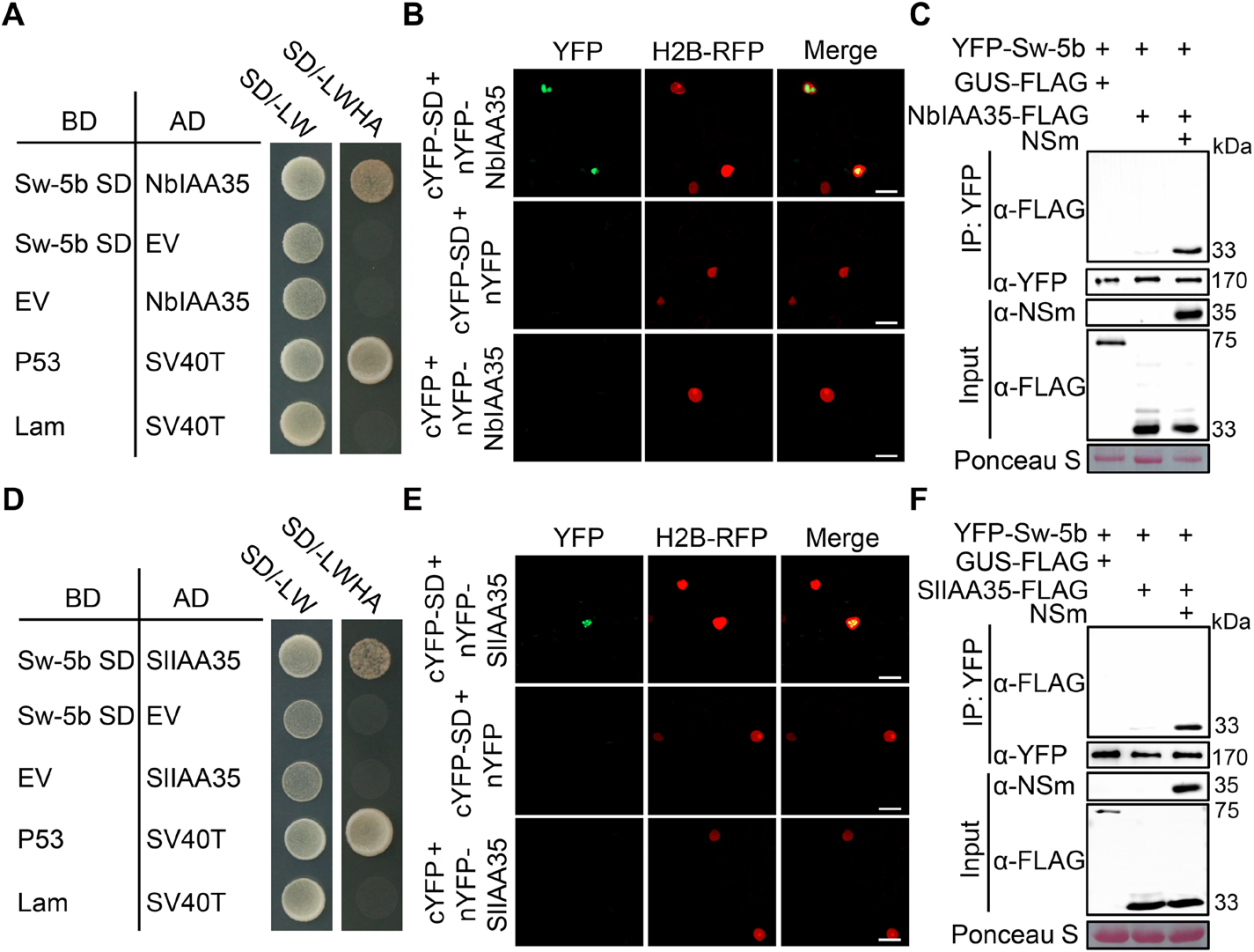
Sw-5b interacts with transcriptional repressor IAA35. (**A** and **D**) Y2H analysis of the interaction between Sw-5b SD and NbIAA35 (A) or SlIAA35 (D). AD, activation domain; BD, DNA binding domain. SV40-T + P53 and SV40-T + Lam were used as positive control and negative control, respectively. Yeast cells were grown on medium SD/-LW and selected on medium SD/-LWHA at 30°C for 4 days. (**B** and **E**) BiFc analysis of the interaction between Sw-5b SD and NbIAA35 (B) or SlIAA35 (E) in *N. benthamiana* leaves. H2B-RFP was used as a marker of nucleus. Twenty-five micromolar MG132 was applied at 16 hpi to prevent protein degradation. Images were taken at 24 hpi. Scale bars, 20 μm. (**C** and **F**) Co-IP analysis of the interaction between YFP-Sw-5b and NbIAA35-FLAG (C) or SlIAA35-FLAG (F) in *N. benthamiana* leaves with or without NSm. Twenty-five micromolar MG132 was applied at 14 hpi, and leaf samples were collected at 22 hpi. Proteins were detected by immunoblotting using YFP and FLAG antibody. Ponceau staining shows protein loading. Experiments were repeated three times with similar results.

Our previous findings demonstrated that Sw-5b relies on its SD to enter the nucleus and initiate immune responses that inhibit the intercellular movement of TSWV (*44*). Since Sw-5b induces robust callose deposition upon activation (Fig. 1, C and D), we next examined the effect of SD on plasmodesmal callose accumulation. As shown in fig. S5 (A to C), the callose level and *NbGSL5/8/10* expression level were higher in YFP-SD–expressing leaf discs than in YFP-expressing controls. Given the positive role of NbGSL5/8/10 in *Sw-5b*–mediated defense against TSWV, we next investigated whether SD could inhibit TSWV infection by up-regulating *GSL5/8/10* expression. We knocked down *NbGSL5/8/10* or *GUS* control by RNAi in *N. benthamiana* leaves and inoculated TSWV at 12 hours postinfiltration (hpi). Twelve hours later, SD was transiently expressed in treated leaves, followed by viral N protein detection at 3 days postinoculation (dpi) (fig. S5D). The results showed that SD overexpression markedly reduced TSWV accumulation, whereas silencing *GSL5/8/10* abolished the SD’s inhibition effect (fig. S5E). To elucidate the mechanistic involvement of SD in *NbGSL5/8/10* up-regulation, we used the SD domain as bait and performed yeast two-hybrid (Y2H) screening against a cDNA library of *N. benthamiana* and identified an SD-interacting protein auxin-responsive protein 35 (NbIAA35, Niben101Scf05720g02003) (fig. S6A), which belongs to the Aux/IAA protein family (*48*). The full-length *NbIAA35* gene was cloned from *N. benthamiana* cDNA, encoding 210 amino acids. Y2H assays further confirmed that SD interacted with NbIAA35 (Fig. 2A). In addition, bimolecular fluorescence complementation (BiFC) assays showed that the coexpression of cYFP-SD and nYFP-NbIAA35 produced green fluorescence signals that overlapped with the red fluorescence of the nuclear marker H2B–red fluorescent protein (RFP), indicating that their interaction occurs in the nucleus (Fig. 2B). Coimmunoprecipitation (Co-IP) experiment revealed a strong interaction between full-length Sw-5b and NbIAA35 in the presence of the viral effector NSm (Fig. 2C). Of note, these interactions were similarly observed between Sw-5b and the tomato IAA35 (SlIAA35, Solyc07g008020) (Fig. 2, D to F).

### IAA35 coordinates with IAA13/27/29/33 to repress *GSL5/8* expression and callose accumulation

**Fig. 3.**
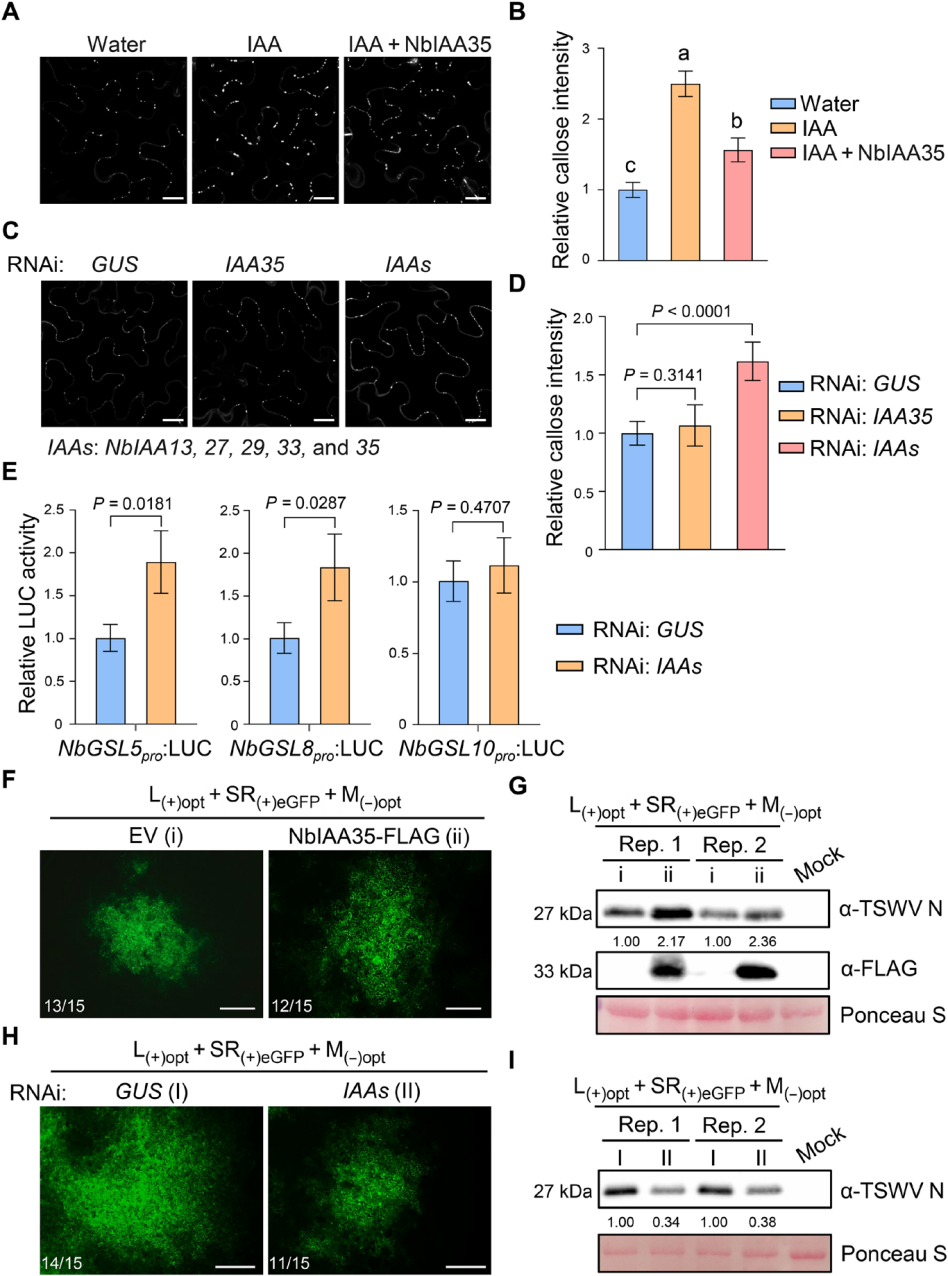
NbIAA13/27/29/33/35 negatively regulate *NbGSL5/8* transcription and callose accumulation and promote TSWV infection. (**A**) Callose fluorescence at PD in *N. benthamiana* leaves treated with water, IAA, or IAA + NbIAA35, respectively. Leaves expressing NbIAA35 were treated with 25 μM IAA at 24 hpi, followed by aniline blue staining at 30 hpi. Scale bars, 20 μm. (**B**) Quantification of callose intensity in (A). Values represent means ± standard deviation [one-way analysis of variance (ANOVA), *n* = 6 biologically independent samples]. Different letters represent significant differences. (**C**) Plasmodesmal callose in leaves silencing *GUS*, *NbIAA35*, or *NbIAAs* (*IAA13/27/29/33/35*), respectively. Scale bars, 20 μm. (**D**) Quantification of callose intensity in (C). Values are means ± standard deviation of six biological replicates. (**E**) Relative LUC activities of reporters driven by *NbGSL5/8/10* promoter in *NbIAAs-*silenced or nonsilenced *N. benthamiana* leaf discs. Values represent means ± standard deviation of three biological replicates. Statistical analysis in (D) and (E) was performed by two-tailed Student’s *t* test. (**F**) Fluorescence images of TSWV infectious clones coexpressed with EV or NbIAA35-FLAG in *N. benthamiana* leaves. Scale bars, 400 μm. (**G**) Western blot analysis of TSWV N accumulation in (F). (**H**) Fluorescence images of TSWV infectious clones in *N. benthamiana* leaves with silencing of *GUS* or *IAAs*. Scale bars, 400 μm. (**I**) Immunoblotting analysis of TSWV N accumulation in (H). Protein levels in (G) and (I) were determined using ImageJ software, and protein loading was indicated by Ponceau staining.

Members in Aux/IAA protein are negative regulators of auxin signaling pathway (*49*). Next, we investigated whether auxin and IAA35 are involved in the regulatory control of *GSL5/8/10* expression and callose deposition. Exogenous application of IAA to *N. benthamiana* leaves significantly up-regulated the expression of *NbGSL5*, *NbGSL8*, and *NbGSL10*, as determined by quantitative real-time polymerase chain reaction (qRT-PCR) (fig. S7A). Correspondingly, callose level was markedly enhanced in both IAA-treated tobacco and tomato leaves compared to water-treated controls (Fig. 3, A and B, and fig. S7, B and C). Notably, the IAA-induced increase in callose accumulation was significantly attenuated by NbIAA35 expression (Fig. 3, A and B). To determine whether IAA35 inhibits the transcription of *GSL5/8/10*, we cloned the *NbGSL5/8/10* promoters and generated luciferase reporter constructs (*NbGSL5/8/10_pro_*: LUC) (fig. S8A). These reporters were transiently coexpressed with either NbIAA35 or EV in *N. benthamiana* leaves. Luciferase assays showed that expression of NbIAA35 significantly reduced LUC activity driven by the promoter of *NbGSL8*, but not *NbGSL5* or *NbGSL10* (fig. S8B), suggesting that *NbGSL8* is transcriptionally repressed by NbIAA35. We next knocked down *NbIAA35* using RNAi-mediated gene silencing (RNAi: *IAA35*) and performed luciferase assays (fig. S8, C and D). The results showed no significant changes in LUC activity driven by *NbGSL5/8/10* promoters (fig. S8E). Consistently, no significant difference in callose intensity was observed between RNAi: *IAA35* and RNAi: *GUS* leaf tissues (Fig. 3, C and D). These data indicate that there might be multiple Aux/IAA proteins that function redundantly with IAA35 to repress *GSL8* expression and callose accumulation.

To test whether any other Aux/IAA proteins that interact with Sw-5b, we cloned all 25 Aux/IAA members from *Solanum lycopersicum* and performed Y2H assays. The results showed that in addition to SlIAA35, the SD domain of Sw-5b also interacted with SlIAA13, SlIAA27, SlIAA29, and SlIAA33 (fig. S6B). We then cloned the homologs of these four genes from *N. benthamiana* and confirmed that NbIAA13, NbIAA27, NbIAA29, and NbIAA33 also interacted with Sw-5b SD in Y2H assays (fig. S6C). To assess the functional relevance of these interactions, we cosilenced *NbIAA35* together with *NbIAA13*, *27*, *29*, and *33* (referred to as RNAi: *IAAs*) (fig. S9, A and B). Luciferase assays showed that silencing these five *IAAs* significantly increased LUC activity driven by *NbGSL5* and *NbGSL8* promoter, compared to the RNAi: *GUS* control (Fig. 3E). Aniline blue staining further revealed a marked increase in callose level in *IAAs*-silenced leaf tissues relative to the nonsilenced control (Fig. 3, C and D). These results suggest that NbIAA13/27/29/33/35 coordinately down-regulate *NbGSL5/8* expression to inhibit callose deposition.

We next investigated whether IAA application could affect TSWV infection. We treated *N. benthamiana* and tomato plants (susceptible cultivars lacking *Sw-5b*) with IAA or water, followed by TSWV inoculation, and monitored disease progression. As shown in fig. S7 (D and F), IAA-treated plants exhibited milder disease symptoms compared to water-treated controls. Notably, disease symptom onset was delayed by approximately 3 days in *N. benthamiana* and 4 days in tomato (fig. S7, E and G), indicating that IAA treatment impedes TSWV systemic movement or symptom development. To further examine the role of NbIAAs in TSWV infection, we coexpressed TSWV infectious clones with NbIAA35 or EV in *N. benthamiana* epidermal cells. At 2.5 dpi, the size of TSWV infection foci were much larger in *NbIAA35*-expressing leaf cells compared to EV control (Fig. 3F). Immunoblotting analysis further confirmed increased TSWV accumulation in leaves overexpressing NbIAA35 (Fig. 3G). Conversely, in leaf tissues cosilenced for *NbIAA13/27/29/33/35* (RNAi: *IAAs*), both the size of infection foci and TSWV levels were significantly reduced compared to RNAi: *GUS* controls (Fig. 3, H and I). To further test whether these Aux/IAAs modulate TSWV resistance by regulating *GSL8/10* expression, we conducted TSWV inoculation assays in *N. benthamiana* leaves expressing RNAi: *IAAs*, RNAi: *GSL5*/*8*/*10*, or RNAi: *IAAs* plus RNAi: *GSL5/8/10* (fig. S10A). Western blot analysis showed that TSWV accumulation was inhibited in RNAi: *IAAs* leaves. However, this inhibition in RNAi: *IAAs* plus RNAi: *GSL5/8/10* leaves was largely compromised (fig. S10).

### ARF19 directly activates *GSL8* transcription and enhances host resistance against TSWV

**Fig. 4.**
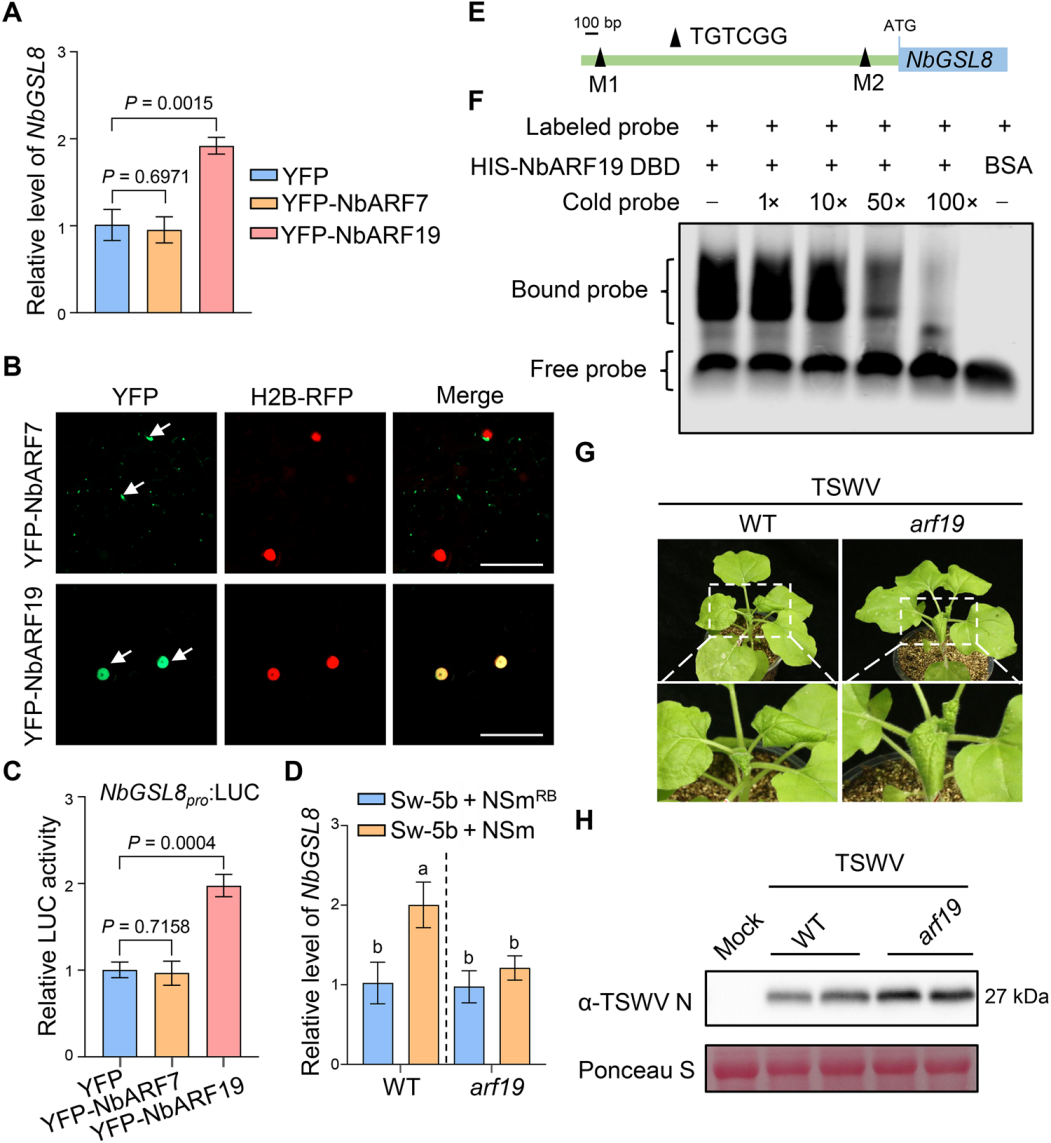
ARF19 directly activates *GSL8* expression to enhance host defense against TSWV. (**A**) Relative expression level of *NbGSL8* in *N. benthamiana* leaves expressing YFP, YFP-NbARF7, or YFP-NbARF19, respectively. Leaf samples were collected at 22 hpi. Values are means ± standard deviation, *n* = 3 biological replicates. (**B**) Subcellular localization of YFP-NbARF7 and YFP-NbARF19 in *N. benthamiana* leaves. H2B-RFP was served as a marker of nucleus. Images were taken at 24 hpi by confocal microscopy. Scale bars, 20 μm. (**C**) Relative LUC activity of reporter driven by *NbGSL8* promoter in *N. benthamiana* leaves expressing YFP, YFP-NbARF7, or YFP-NbARF19, respectively. Values were means ± standard deviation, *n* = 3 biological replicates. (**D**) Relative expression level of *NbGSL8* in WT or *arf19* plants coexpressing Sw-5b with NSm^RB^ or NSm by qRT-PCR. Values were means ± standard deviation (two-way ANOVA, *n* = 3 biological replicates). Different letters represent significant differences. (**E**) Schematic diagrams of two AuxREs (M1 and M2) in *NbGSL8* promoter. (**F**) EMSA analysis for the binding of NbARF19 DBD to *NbGSL8* promoter. The experiment was repeated three times with similar results. (**G**) Symptoms of WT and *arf19* mutant plants inoculated with TSWV. Photos were taken at 9 dpi. (**H**) Accumulation of TSWV N in systemic leaves of WT or *arf19* plants by immunoblotting. Ponceau staining shows protein loading. Leaf samples in (A) to (D) were collected at 22 hpi. For (A) and (C), data were analyzed by two-tailed Student’s *t* test.

The auxin-ARF7-GSL8 feedback circuit has been reported to induce PD callose deposition during the hypocotyl tropic response in *Arabidopsis thaliana* (*50*). Y2H assay confirmed the interaction between NbARF7 and NbIAA35 (fig. S11A), so we examined the potential role of NbARF7 in regulating *GSL8* expression in *N. benthamiana* leaf tissues. However, overexpressing YFP-NbARF7 did not affect the expression level of *NbGSL8* (Fig. 4A) despite successful expression of the recombinant protein confirmed by immunoblotting (fig. S11B). Unexpectedly, confocal microscopy analysis showed that both YFP-NbARF7 and NbARF7-YFP localized predominantly to the cytoplasm but not to the nucleus (Fig. 4B and fig. S11C).

ARF19 had the closest relationship with ARF7, and the two function redundantly in several biological processes in *Arabidopsis* (*51*–*53*). We next performed BLAST search using AtARF19 protein sequence against the Sol Genomics database and identified two candidate homologs, named NbARF19 and NbARF19.1, in *N. benthamiana* (fig. S11D). Y2H assay showed that NbARF19, but not NbARF19.1, interacted with NbIAA35 (fig. S10A). Moreover, confocal microscopy analysis showed that YFP-NbARF19 colocalized with the nuclear marker H2B-RFP, confirming its nuclear localization (Fig. 4B). The qRT-PCR analysis further revealed a significant increase in *NbGSL8* transcript levels in leaves expressing YFP-NbARF19 compared to leaves expressing YFP alone (Fig. 4A). Moreover, luciferase assay showed that the expression of YFP-NbARF19, but not YFP-NbARF7, led to a marked increase in LUC activity driven by *NbGSL8* promoter compared to YFP control (Fig. 4C), indicating transcriptional activation of the *NbGSL8* promoter by NbARF19. We next generated *Nbarf19* mutant *N. benthamiana* plants using CRISPR-Cas9–based gene editing and observed lightly downward leaf-curling (fig. S12, A and B). Next, we coexpressed Sw-5b and NSm or NSm^RB^ in both wild-type (WT) and *arf19* plants and performed qRT-PCR. In contrast to WT plants, Sw-5b failed to up-regulate *NbGSL8* expression in *arf19* plants (Fig. 4D).

ARFs regulate gene expression by binding to auxin-responsive elements (AuxREs) in the promoters of target genes (*54*), and sequence analysis revealed two AuxREs (M1 and M2) in the *NbGSL8* promoter (Fig. 4E). To determine whether NbARF19 directly binds to the *NbGSL8* promoter, we expressed and purified the DNA binding domain (DBD) of NbARF19 from *Escherichia coli* and performed electrophoretic mobility shift assay (EMSA) using an Alexa Fluor 660–labeled *NbGSL8* probe containing M2 motif (probe sequence is listed in table S2). As shown in Fig. 4F, the addition of NbARF19 DBD, but not bovine serum albumin (BSA), caused an evident shift of probe bands. Furthermore, excess unlabeled probe competed effectively with the labeled probe, confirming the binding specificity (Fig. 4F).

To investigate the role of ARF19 in TSWV infection, we mechanically inoculated TSWV in *arf19*-knockout *N. benthamiana* plants and *ARF19-*knockdown tomato plants (fig. S13, A and B) and monitored disease development. As shown in Fig. 4 (G and H), *arf19* mutant plants exhibited more severe symptoms and higher viral accumulation than WT plants. Consistently, TSWV-inoculated *SlARF19*-silenced tomato plants exhibited more severe symptoms and higher viral accumulation than TRV-*GUS*–treated control plants (fig. S13, C and D). These data suggest that ARF19 positively regulates host defense against TSWV.

To further assess whether ARF19 contributes to *Sw-5b*–mediated inhibition of TSWV movement and accumulation, we silenced *NbARF19* (RNAi: *ARF19*) in *Sw-5b*–transgenic *N. benthamiana* leaves followed by TSWV inoculation (fig. S14, A and B). Aniline blue staining revealed a significant reduction in callose levels in *ARF19*-silenced leaf samples compared to nonsilenced controls (fig. S14, C and D). Moreover, both the size of HR foci and the accumulation of TSWV NP were markedly increased in *ARF19*-silenced leaf tissues (fig. S14, E to G). We also silenced *SlARF19* in *S. lycopersicum* plants containing *Sw-5b* using VIGS and then inoculated TSWV. Similarly, silencing *SlARF19* led to increased HR lesion size and higher accumulation of TSWV compared to *GUS* controls (fig. S13, E to G). Together, these results suggest that ARF19 plays a crucial role in *Sw-5b*–mediated antiviral immunity by directly activating *GSL8* expression to promote callose deposition.

### Sw-5b interferes with IAA13/27/29/33/35-ARF19 interaction, alleviating the repression on ARF19 to induce callose deposition

**Fig. 5.**
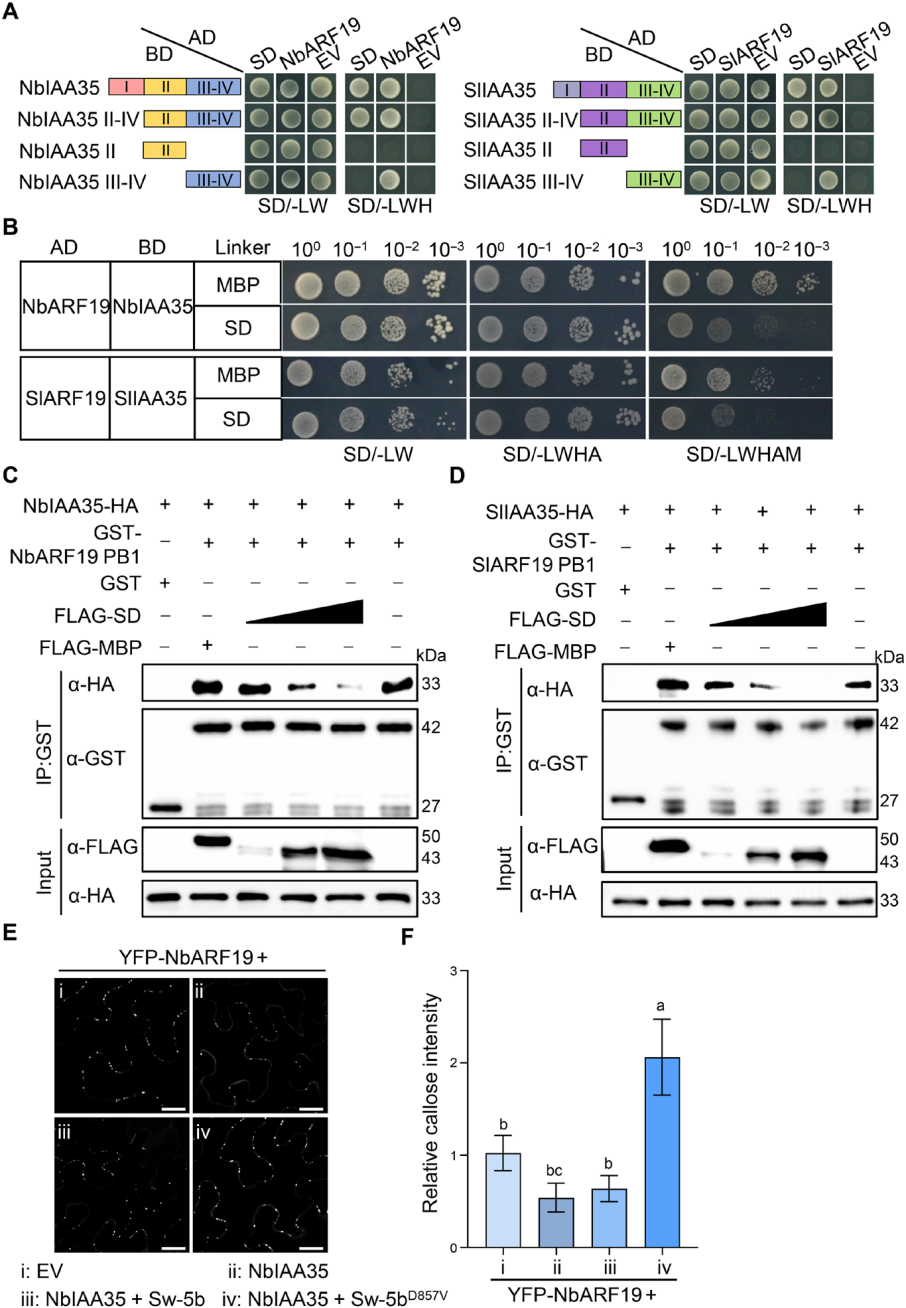
Sw-5b depresses Aux/IAAs-mediated repression on ARF19 to activate callose deposition. (**A**) Y2H showing the domains of IAA35 responsible for the interaction with SD or with ARF19. Yeast cells were grown on medium SD/-LW and selected on medium SD/-LWH at 30°C for 4 days. (**B**) Y3H analysis showing the effect of Sw-5b SD on the interaction between ARF19 and IAA35. Yeast cells were grown on medium SD/-LW and selected on medium SD/-LWHAM at 30°C for 4 days. (**C** and **D**) Competitive GST pull-down analysis of FLAG-SD on the interaction between GST-NbARF19 PB1 and NbIAA35-HA (C) or between GST-SlARF19 PB1 and SlIAA35-HA (D). Purified GST-ARF19 PB1 was incubated with IAA35-HA and increasing amount of FLAG-SD. (**E**) Callose fluorescence upon aniline blue staining in leaves coexpressing YFP-NbARF19 with EV, NbIAA35, NbIAA35 + Sw-5b, or NbIAA35 + Sw-5b^D857V^, respectively. Images were taken at 22 hpi. Scale bars, 20 μm. (**F**) Quantification of the callose intensity in (E). Values are means ± standard deviation, *n* = 6 biologically independent samples. Statistical analysis was performed by one-way ANOVA, and different letters represent significant differences. For (A) to (D), the experiments were repeated three times with similar results.

As IAA35 interacted with ARF19 and inhibited callose deposition, we sought to understand how Sw-5b activates *GSL* expression via Aux/IAAs and ARF19 modules. BiFC assay showed that ARF19-IAA35 interaction occurred in the nucleus (fig. S15A), which is also the location where Sw-5b SD interacts with IAA35 (Fig. 2, B and E), raising the possibility that SD and ARF19 may competitively bind to IAA35. Canonical Aux/IAA proteins harbor four conserved domains and interact with ARFs via domain III−IV, also known as PB1 domain (*49*). To investigate whether IAA35 associates with Sw-5b SD and ARF19 via the same domain, we conducted Y2H assays. The results showed that ARF19 interacted with the domain II−IV and III−IV regions of IAA35 (Fig. 5A). Notably, both NbIAA35 and SlIAA35 interacted with SD domain via their domain II−IV regions (Fig. 5A). To assess whether SD interferes with the ARF19-IAA35 interaction, we conducted a yeast three-hybrid (Y3H) assay using SD or maltodextrin binding protein (MBP) as the third component. Compared to MBP control, the interaction between IAA35 and ARF19 was significantly reduced in the presence of SD (Fig. 5B). Similarly, glutathione *S*-transferase (GST) pull-down assay also showed that hemagglutinin-tagged IAA35 (IAA35-HA) interacted with GST-ARF19 PB1 in vitro, and the amount of IAA35-HA pulled down by GST-ARF19 PB1 decreased as the amount of FLAG-SD increased (Fig. 5, C and D). In contrast, the addition of FLAG-MBP had no effect on IAA35-ARF19 PB1 interaction (Fig. 5, C and D). To further confirm the effect of Sw-5b on the ARF19-IAA35 interaction in vivo, we performed BiFC assay by coexpressing cYFP-ARF19 and nYFP-IAA35 along with either EV, Sw-5b SD, Sw-5b, or its autoactive mutant Sw-5b^D857V^. As shown in fig. S15 (B and C), SD expression led to a reduction in the intensity of fluorescent spots in leaf discs compared to the EV control. The addition of Sw-5b had little effect on the ARF19-IAA35 interaction. Notably, the expression of auto-active Sw-5b^D857V^ caused a pronounced reduction in the intensity of fluorescent spots.

Since Sw-5b SD domain also interacts with IAA13, 27, 29, and 33, we further examined whether SD disrupts the interactions between ARF19 and these Aux/IAA proteins. Y2H assays showed that all four Aux/IAAs directly interacted with ARF19 (fig. S16, A and B). Y3H assays further confirmed that the presence of SD impaired the interactions between ARF19 and IAA13/27/29/33, respectively (fig. S16, C and D). These results indicate that Sw-5b interferes with the interaction between ARF19 and multiple Aux/IAA proteins via its SD domain.

We also examined whether Sw-5b can depress the IAA35-mediated repression on ARF19. As shown in Fig. 5 (E and F), overexpression of NbIAA35 led to a 50% reduction in callose intensity in cells coexpressing NbARF19, while the addition of the inactive state Sw-5b had little effect on this suppression. However, coexpressing the autoactive mutant Sw-5b^D857V^ resulted in more than a twofold increase in callose level. These data suggest that Sw-5b interferes with Aux/IAAs-ARF19 interactions to promote callose accumulation.

### Sw-5b induces auxin biosynthesis via *YUC8* under the transcriptional control of ARF19

**Fig. 6.**
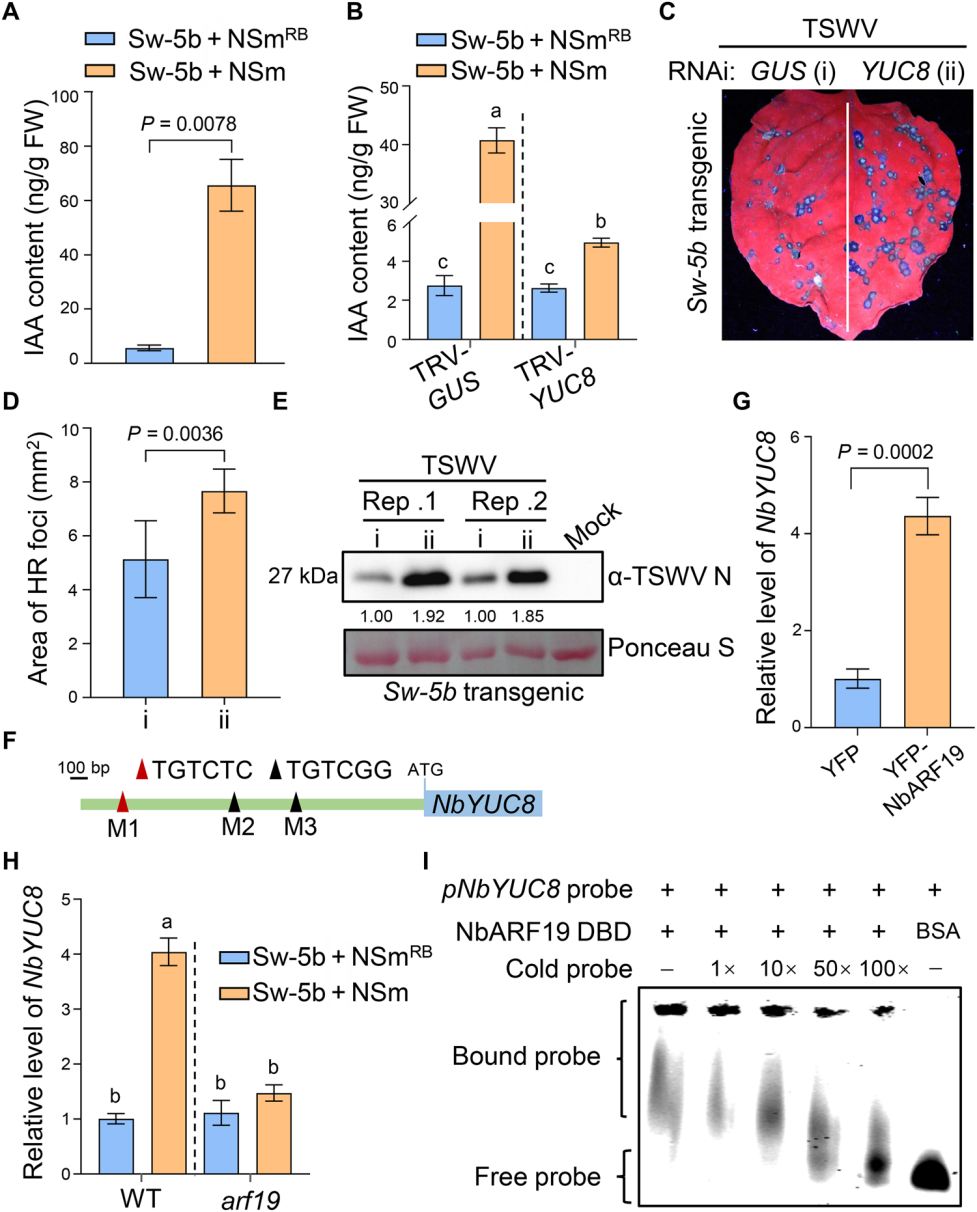
Sw-5b induces auxin biosynthesis via *YUC8* under the transcriptional control of ARF19. (**A**) LC-MS/MS showing the IAA content in *N. benthamiana* leaves coexpressing Sw-5b with NSm or NSm^RB^. (**B**) LC-MS/MS analysis of IAA content in TRV-*GUS* or TRV-*YUC8 N. benthamiana* plants coexpressing Sw-5b with NSm^RB^ or NSm, respectively. (**C**) HR foci in *YUC8-* or *GUS*-silenced leaves from *Sw-5b*–transgenic plants inoculated with TSWV. Photos were taken at 3 dpi under a UV light. (**D**) Quantification of mean areas of HR foci in (C). Values are means ± standard deviation, *n* = 6 biologically independent leaf samples. (**E**) Immunoblotting analysis of TSWV N accumulation in (C). Protein levels were determined by ImageJ software and Ponceau S–stained bands show protein loading. (**F**) Schematic diagrams of three AuxREs (M1, M2, and M3) in *NbGSL8* promoter. (**G**) Relative expression level of *NbYUC8* in *N. benthamiana* leaves expressing YFP or YFP-NbARF19 by qRT-PCR. Values are means ± standard deviation, *n* = 3 biologically independent replicates. (**H**) Relative expression levels of *NbYUC8* in WT or *arf19* plants coexpressing Sw-5b with NSm^RB^ or NSm, respectively. Values in (B) and (H) represent means ± standard deviation (two-way ANOVA, *n* = 3 biologically independent replicates). Different letters represent significant differences. (**I**) EMSA showing the binding of NbARF19 DBD to *NbYUC8* promoter. The experiment was repeated three times with similar results. Statistical analysis in (A), (D), and (G) was performed by two-tailed Student’s *t* test.

To investigate whether the interaction between Sw-5b and transcriptional repressor Aux/IAAs activates the auxin signaling pathway, we analyzed the transcriptome data described earlier and found that in samples coexpressing Sw-5b and NSm, numerous auxin-responsive genes, including those from the *SAUR*, *GH3*, and *Aux/IAA* families, were up-regulated (fig. S17A). qRT-PCR further confirmed that auxin-responsive genes *NbIAA15*, *NbSAUR36*, and *NbGH3.2* were significantly up-regulated in leaves coexpressing Sw-5b and NSm (fig. S17B). We further measured the IAA content using liquid chromatography–tandem mass spectrometry (LC-MS/MS), and the result showed that the IAA content was significantly increased in leaf tissues coexpressing Sw-5b and NSm (Fig. 6A). We also examined whether SD itself could activate auxin signaling. As shown in fig. S18 (A and B), the expression of YFP-SD caused a marked increase in both IAA content and auxin-responsive gene levels compared to YFP control. Next, we performed mechanical inoculation of TSWV on tomato plants (containing *Sw-5b*) and measured changes in the transcription levels of auxin-responsive genes and IAA content at 2 dpi. Compared with mock-inoculated controls, TSWV inoculation significantly up-regulated the expression levels of *SlIAA15*, *SlSAUR36*, and *SlGH3.2* (fig. S19A) and also increased leaf IAA content (fig. S19B). These results suggest that Sw-5b activates auxin biosynthesis and signaling pathways in both *N. benthamiana* and *S. lycopersicum* plants upon recognition of viral effector.

To elucidate the underlying mechanism of IAA up-regulation following Sw-5b activation, we focused on the YUCCA (YUC) family of flavin monooxygenases. These enzymes are known to catalyze a rate-limiting step in the tryptophan-dependent auxin biosynthesis pathway (*55*). RNA-seq analysis identified four *YUC* genes *NbYUC5*, *NbYUC6*, *NbYUC8*, and *NbYUC10* that were differentially expressed in leaf tissues coexpressing Sw-5b and NSm (fig. S17C). Among these, only *NbYUC8* was significantly up-regulated, while the other three were expressed in a lower level and were down-regulated upon Sw-5b activation (fig. S17D). Therefore, *NbYUC8* was selected for further functional analysis. We used TRV-based gene silencing to knock down *NbYUC8* in *N. benthamiana* and observed no obvious phenotypic difference between *GUS*-control and *NbYUC8*-silenced plants (fig. S20, A and B). To determine whether *NbYUC8* contributes to IAA biosynthesis upon Sw-5b activation, we measured IAA levels using LC-MS/MS in TRV-*GUS* and TRV-*YUC8* plants coexpressing Sw-5b with NSm or NSm^RB^. The result showed that the *Sw-5b*–induced IAA accumulation was reduced by approximately 90% in *NbYUC8*-silenced plants, indicating that NbYUC8 is essential for *Sw-5b*–mediated IAA biosynthesis (Fig. 6B). Consistent with this, qRT-PCR analysis revealed that *Sw-5b*–induced up-regulation of auxin-responsive genes were dramatically reduced in *NbYUC8*-silenced plants compared to the TRV-*GUS* plants (fig. S20C). In contrast, overexpression of NbYUC8 led to a significant increase in the expression of auxin-responsive genes (fig. S20D). These results suggest that Sw-5b induces IAA biosynthesis and signaling through activation of *YUC8*.

Since IAA treatment enhances host immunity against TSWV, we next investigated the role of *YUC8* during TSWV infection. We coexpressed TSWV infectious clones carrying GFP reporter with NbYUC8 or EV in *N. benthamiana* leaves and observed smaller infection foci and decreased viral NP accumulation in NbYUC8-expressing leaves compared to those expressing EV (fig. S20, E and F). In contrast, knockdown of *NbYUC8* resulted in larger infection foci and higher TSWV accumulation (fig. S20, G and H). To further examine whether YUC8 is essential for *Sw-5b*–mediated defense against TSWV, we silenced *NbYUC8* in *Sw-5b* transgenic *N. benthamiana* leaves, followed with mechanical inoculation of TSWV (fig. S21, A and B). Aniline blue staining revealed that plasmodesmal callose intensity was markedly reduced in *YUC8*-silenced half-leaves compared to nonsilenced controls (fig. S21, C and D). In addition, the average size of HR foci and the viral accumulation were significantly greater in *NbYUC8*-silenced half-leaves than in controls (Fig. 6, C to E), indicating that *YUC8* positively regulates host defense against TSWV and is critical for *Sw-5b*–mediated immunity in *N. benthamiana*.

We subsequently investigated the mechanism by which *NbYUC8* is up-regulated upon Sw-5b activation. Analysis of the *NbYUC8* promoter sequence revealed the presence of three AuxRE motifs (M1, M2, and M3) (Fig. 6F), indicating that *NbYUC8* might be regulated by ARFs. Since overexpression of SD could induce auxin accumulation (fig. S18A), we examined the effect of IAA13/27/29/33/35 on IAA accumulation. As shown in fig. S9 (C and D), both the IAA content and the expression level of auxin-responsive genes in RNAi: *IAAs* leaf tissues were higher than those in RNAi: *GUS* control, leaving the possibility that NbARF19 activates *NbYUC8* expression. As shown in Fig. 6G, overexpression of YFP-NbARF19 significantly increased the expression level of *NbYUC8*. On the basis of this result, we coexpressed Sw-5b and NSm in WT and *arf19* mutant *N. benthamiana* plant leaves. Sw-5b/NSm coexpression significantly up-regulated *NbYUC8* expression, whereas this effect was abrogated in the *arf19* mutant (Fig. 6H). To examine whether NbARF19 directly binds to *NbYUC8* promoter, we performed EMSA using Alexa Flour 660–labeled *NbYUC8* probe (probe sequence is listed in table S2) and HIS-tagged NbARF19 DBD. A clear binding signal was observed between the probe and NbARF19 DBD, but not with BSA, and this binding was competitively inhibited by the addition of unlabeled probe (Fig. 6I). These data indicate that ARF19-YUC8 module contributes to auxin biosynthesis and defense against TSWV during *Sw-5b*–mediated immunity.

### Auxin receptor TIR1/AFBs amplifies immune signaling induced by Sw-5b

**Fig. 7.**
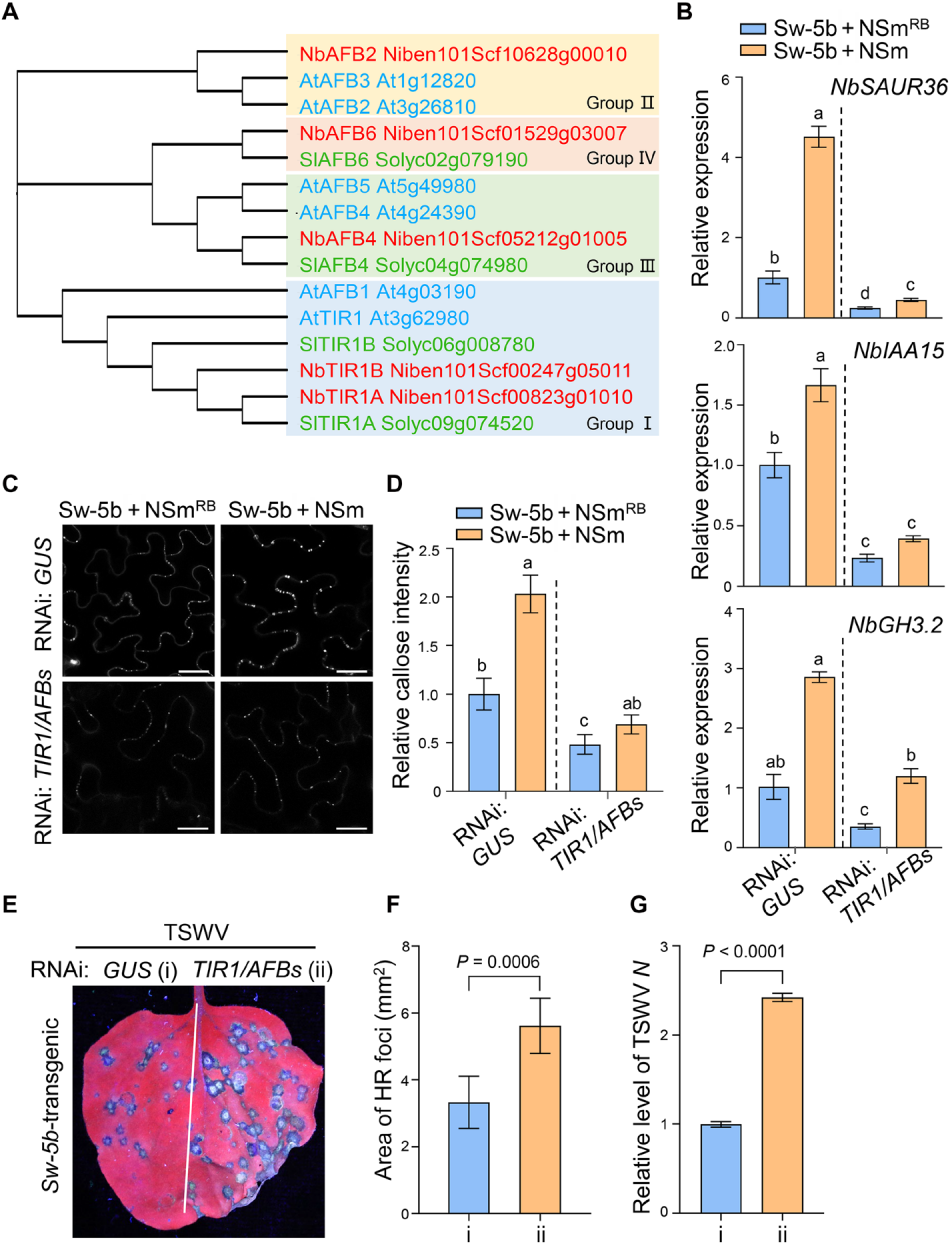
Knockdown of *TIR1/AFBs* attenuates Sw-5b–activated auxin signaling and callose deposition. (**A**) Phylogenetic analysis of TIR1/AFB family proteins in *A. thaliana*, *S. lycopersicum*, and *N. benthamiana.* The tree was constructed in MAGA7 software using a neighbor-joining method (Poisson model, bootstrap = 1000). (**B**) Relative expression levels of auxin-responsive genes in *GUS-* or *TIR1/AFBs*-silenced leaves coexpressing Sw-5b with NSm NSm^RB^ by qRT-PCR. Values represent means ± standard deviation of three biologically independent replicates. (**C**) Fluorescence images of plasmodesmal callose in *GUS-* or *TIR1/AFBs*-silenced half-leaves coexpressing Sw-5b with NSm^RB^ or NSm. Scale bars, 20 μm. (**D**) Quantification of the callose intensity in (C). Values are means ± standard deviation, *n* = 6 biologically independent samples). Statistical analysis in (B) and (D) was performed by two-way ANOVA, and different letters represent significant differences. (**E**) HR foci in *GUS*- or *TIR1/AFBs*-silenced half-leaves from *Sw-5b*–transgenic plants inoculated with TSWV. Photos were taken at 3 dpi under a UV light. (**F**) Quantification of mean areas of HR foci in (E). Values represent means ± standard deviation, *n* = 6 biologically independent leaf samples. (**G**) Relative expression levels of TSWV *N* in leaf samples from (E). Values are means ± standard deviation of three biologically independent replicates. Statistical analysis in (F) and (G) was performed by two-tailed Student’s *t* test.

Auxin receptors TIR1/AFBs are essential for the activation of downstream auxin signaling at high IAA concentration (*13*). As shown above, Sw-5b activation triggered a substantial increase in IAA level through ARF19-YUC8 module (Fig. 6). We wonder whether the increased IAA can promote the immune signaling initiated by Sw-5b-Aux/IAAs-ARF19 interaction. Using BLAST homology searches with AtTIR1 and conserved domain analysis, we identified five TIR1/AFB family members in *N. benthamiana*, whereas *A. thaliana* and *S. lycopersicum* have six and four, respectively. A neighbor-joining phylogenetic tree constructed with these proteins clustered them into four groups (Fig. 7A). Notably, group IV lacked *Arabidopsis* TIR1/AFB members, while none of the tomato TIR1/AFBs belonged to group II (Fig. 7A). Next, we silenced *NbTIR1A/B* together with *AFB4* and *AFB6* (belonging to groups I, III, and IV) by dsRNA-induced RNAi in *N. benthamiana* leaves and expressed NbIAA35-FLAG in RNAi-treated leaves, followed with application of IAA or water (fig. S22, A and B). Western blot analysis showed that IAA treatment caused a reduction in NbIAA35 accumulation in RNAi: *GUS* leaf samples. However, in *NbTIR1/AFBs*-silenced leaves, exogenous IAA failed to induce NbIAA35 degradation (fig. S22C), suggesting that *NbTIR1/AFBs* are responsible for NbIAA35 turnover. To determine whether *TIR1/AFBs* are required for *Sw-5b*–mediated activation of auxin signaling and plasmodesmal callose deposition, we coexpressed Sw-5b and NSm or NSm^RB^ in *TIR1/AFBs*-silenced or nonsilenced leaves (fig. S22A). qRT-PCR showed that Sw-5b could still up-regulate the expression of *NbSAUR36*, *NbIAA15*, and *NbGH3.2* in *TIR1/AFBs*-silenced leaf samples, but the expression levels were largely lower than those in the RNAi: *GUS* control (Fig. 7B). Consistently, the enhanced callose level and increased *NbGSL5/8/10* expression by Sw-5b/NSm coexpression were markedly attenuated in *TIR1/AFBs*-silenced leaves compared to those in nonsilenced samples (Fig. 7, C and D, and fig. S22D). We also expressed SD or EV in *NbTIR1/AFBs*-silenced leaves and analyzed the expression of auxin-responsive genes (fig. S23A). qRT-PCR showed that the up-regulation of *NbSAUR36*, *NbIAA15*, and *NbGH3.2* by SD was also attenuated in *TIR1/AFBs*-silenced leaf samples (fig. S23B). These data suggest that TIR1/AFB receptors are essential for amplification of Sw-5b–induced immune signals.

Given the positive role of auxin in defense against TSWV, we next examined whether *TIR1/AFBs* are necessary for this antiviral function. We expressed TSWV infectious clones in RNAi: *GUS* and RNAi: *TIR1/AFBs* half-leaves and monitored infection (fig. S22A). The result showed that silencing *TIR1/AFBs* led to larger viral infection foci and higher viral accumulation than in the nonsilenced *GUS* control leaf cells (fig. S22, E and F). We also performed TSWV inoculation on *TIR1/AFBs*-silenced leaves expressing SD or EV. Viral accumulation analysis by immunoblotting showed that the inhibitory effect of SD on TSWV was markedly attenuated in RNAi: *TIR1/AFBs* leaves compared with the RNAi: *GUS* control (fig. S23C), indicating that *TIR1/AFBs* are essential for SD-mediated resistance to TSWV. To further examine whether *TIR1/AFBs* contribute to *Sw-5b*–mediated inhibition of TSWV movement, we inoculated TSWV in *Sw-5b*–transgenic *N. benthamiana* leaves with silencing of *TIR1/AFBs* or nonsilenced control (fig. S22A). HR lesions in *TIR1/AFBs*-silenced half-leaves were significantly larger than those in controls (Fig. 7, E and F), and qRT-PCR confirmed the increased TSWV accumulation upon silencing *TIR1/AFBs* (Fig. 7G). In tomato plants that harbor *Sw-5b*, VIGS silencing of *SlTIR1/AFBs* led to stunted plant growth (fig. S24, A and B). Following TSWV inoculation, *SlTIR1/AFBs*-silenced plants exhibited enlarged HR foci and elevated TSWV *N* mRNA levels compared to nonsilenced controls (fig. S24, C to E). Collectively, these results suggest that auxin receptors TIR1/AFBs are essential for *Sw-5b*–activated auxin signaling and antiviral defense.

### SD-containing NLR R8 also activates auxin signaling pathway to promote callose deposition

Besides Sw-5b, *Solanaceae* CNLs including R8, Mi-1.2, Rpi-blb2, and Hero all contained an SD at their N termini (fig. S25A) (*56*). We investigated whether SDs from these CNLs interact with IAA13/27/29/33/35. Y2H assay showed that all these SDs from R8, Mi-1.2, Rpi-blb2, and Hero could interact with NbIAA13 and NbIAA27. However, none of them interacted with NbIAA29/33/35 (fig. S25B).

Given the positive role of auxin in host defense against *Phytophthora* pathogen (*19*), we further selected R8, an NLR confers the resistance to *Phytophthora infestans* (*57*) and examined whether it can activate auxin signaling upon recognition of effector AVR8. As shown in fig. S22C, R8 significantly up-regulated the expression levels of *NbIAA15*, *NbSAUR36*, and *NbGH3.2* upon recognition of AVR8. The IAA content in R8/AVR8 coexpression leaf samples was much more than that in R8/AVR8_m_ (a mutant that was incapable of activating R8) (fig. S25D). Consistently, coexpressing R8 and AVR8 caused a marked increase in *NbGSL8* mRNA level and induced pronounced callose deposition at PD (fig. S25, E to G). These results indicated that R8 also activates the auxin signaling to induce callose deposition.

## DISCUSSION

In this study, we demonstrated that upon recognition of viral MP NSm, Sw-5b induces marked callose deposition at PD, while knocking down *GSL5/8/10* attenuates the ability of Sw-5b in inducing plasmodesmal closure and inhibiting viral intercellular movement. Auxin application induces *GSL5/8/10* expression and callose accumulation, and this process is suppressed by multiple Aux/IAA proteins IAA13/27/29/33/35 through their interaction with the transcriptional activator ARF19. Knockout of *ARF19* significantly decreases *Sw-5b*–induced *NbGSL8* transcription, as well as callose deposition. We found that Sw-5b NLR interferes with the interaction between repressors and ARF19 and depresses the repression of IAA13/27/29/33/35 on ARF19, thereby inducing callose synthesis by up-regulating the expression of callose synthases *GSL5/8/10*. We also found that ARF19 directly regulates the expression of *YUC8*, a key gene in auxin biosynthesis, to further amplify the signal for callose production through auxin receptor TIR1/AFBs in *Sw-5b*–mediated immunity. Therefore, Sw-5b NLR initiates and amplifies auxin signaling through Aux/IAAs-ARF19 module and YUC8-Auxin-TIR1/AFBs circuit to induce robust callose deposition and immunity against viral pathogen invasion. Moreover, another SD-containing NLR R8 also interacts with IAA13 and IAA27 via SD and activates auxin signaling and callose deposition in cell wall.

Phytohormone auxin has been implicated in host defense against a broad range of pathogens, including fungi, oomycetes and viruses (*58*). However, the molecular mechanisms underlying auxin-mediated antiviral defense are poorly understood. Zhang and colleagues have shown that auxin reduced host susceptibility to *rice black-streaked dwarf virus* (RBSDV) (*59*). In rice, OsWRKY13 is responsible for OsARF12-mediated defense against RDV (*20*). In this study, we demonstrated that auxin enhances host resistance to TSWV through promoting plasmodesmal callose deposition via NbGSL5/8/10. Both auxin biosynthesis and signal transduction are essential for auxin-induced immunity to TSWV, as silencing/knockout of either *YUC8*, *TIR1/AFBs*, or *ARF19* facilitates TSWV infection. OsARF12 and OsARF16 play a positive role in antiviral resistance against RDV in rice (*20*). In *Arabidopsis*, ARF7 regulates *GSL8*-callose module during hypocotyl tropic growth (*50*). However, in this work, NbARF7 failed to regulate *NbGSL8* transcription in the leaf epidermal cells of *N. benthamiana*, which may be attributable to its predominant cytoplasmic localization. We showed that in *N. benthamiana* and *S. lycopersicum* plants, ARF19 positively regulates *Sw-5b*–mediated resistance against TSWV, through directly activating *GSL8* expression and callose deposition. These findings point to a central role for nuclear-localized ARF19 in coordinating auxin-dependent antiviral immunity in *N. benthamiana* leaves.

Our results showed that Sw-5b activates auxin biosynthesis and signaling upon recognition of NSm. At high auxin concentrations, IAA induces the association of auxin receptors TIR1/AFBs with Aux/IAA repressors, depressing the suppression of Aux/IAAs on ARFs to activate target genes (*13*, *14*, *49*). Sw-5b interacts with Aux/IAA repressors IAA13/27/29/33/35 via SD domain and interferes with Aux/IAA-ARF19 interaction, thereby initiating the auxin signaling and callose deposition via Aux/IAA-ARF module. However, the abundance of NLRs in plants is strictly regulated and maintained at a lower level; thus, this initiation of the auxin signaling by Sw-5b NLR may not be sufficient to induce a robust callose deposition. We found that Sw-5b activates YUC8-TIR1/AFBs-ARF19 signaling circuit for massive IAA production to boost the callose deposit signaling. Correspondently, IAA13/27/29/33/35 repressors were degraded in Sw-5b SD–overexpressed leaves (fig. S26). The YUC8-TIR1 module is essential for sufficient callose deposition since silencing of either *YUC8* or *TIR1/AFBs* markedly attenuated the *GSL8* expression and callose production induced by Sw-5b. The defense against TSWV by *Sw-5b* was also impaired in both *N. benthamiana* and *S. lycopersicum* plants knocking down *YUC8*- or *TIR1/AFBs*. In the *arf19* knockout mutant, Sw-5b failed to induce *NbYUC8* and *NbGSL8* expression, confirming the requirement of *YUC8*-auxin-TIR1/AFBs-ARF19 cascade for immune signaling amplification. Only *YUC8* was up-regulated, but other *YUCs* was down-regulated, suggesting that auxin signaling is likely tightly regulated by NLR receptors.

Upon activation, Sw-5b NLR induced callose deposition, a hallmark PTI and ETI response (*28*), to restrict the intercellular movement of TSWV. Although GSL enzymes catalyze callose synthesis, their involvement in ETI-mediated callose production remains uncharacterized. Here, we found that callose synthases *GSL5*, *GSL8*, and *GSL10* were specifically up-regulated by Sw-5b NLR. Overexpressing each of NbGSL5, NbGSL8, and NbGSL10 resulted in callose deposition, which inhibited intercellular movement of TSWV. As intercellular viral movement occurs through PD (*30*), Sw-5b has induced several GSLs to effectively deposit callose and restrict viral spread to neighboring cells. NLR can typically trigger a hypersensitive cell death and eliminate the pathogens within the localized cells (*1*, *11*). Silencing either *ARF19*, *TIR1*, *YUC8*, or *GSL5*/*8*/*10* leads to enlarged HR loci induced by Sw-5b. These findings suggest that callose deposition at PD can confine viral pathogen within initially infected cells. The callose-mediated physical restriction and programmed cell death may synergistically eliminate the pathogens at the infection site.

The Sw-5b SD is a multifunctional domain that strengthens the inhibition of CC on NB-LRR (*60*), perceives and interacts with low amount of effector NSm (*41*, *61*), and is responsible for Sw-5b’s nuclear localization (*44*). In this study, we found that Sw-5b SD interacts with transcriptional repressors IAA13/27/29/33/35 in auxin signaling. Other SD-containing NLR proteins including R8, Rpi-blb2, Mi-1.2, and Hero also interact with NbIAA13/27. Similar to Sw-5b, R8 can activate auxin biosynthesis and signaling, as well as callose deposition upon recognition of effector AVR8. Other SD-NLRs, like Mi-1.2 and Rpi-blb2, may similarly modulate auxin signaling. A previous study has shown that auxin signaling positively regulates host immunity against *Phytophthora* pathogen (*19*). These studies, together with our findings, indicate that auxin pathway have important roles in defense against different pathogens. Our findings also suggest that SD-containing NLRs use a conserved mechanism to activate auxin signaling pathway and to induce callose deposition in cell wall for defense against pathogen invasions.

**Fig. 8.**
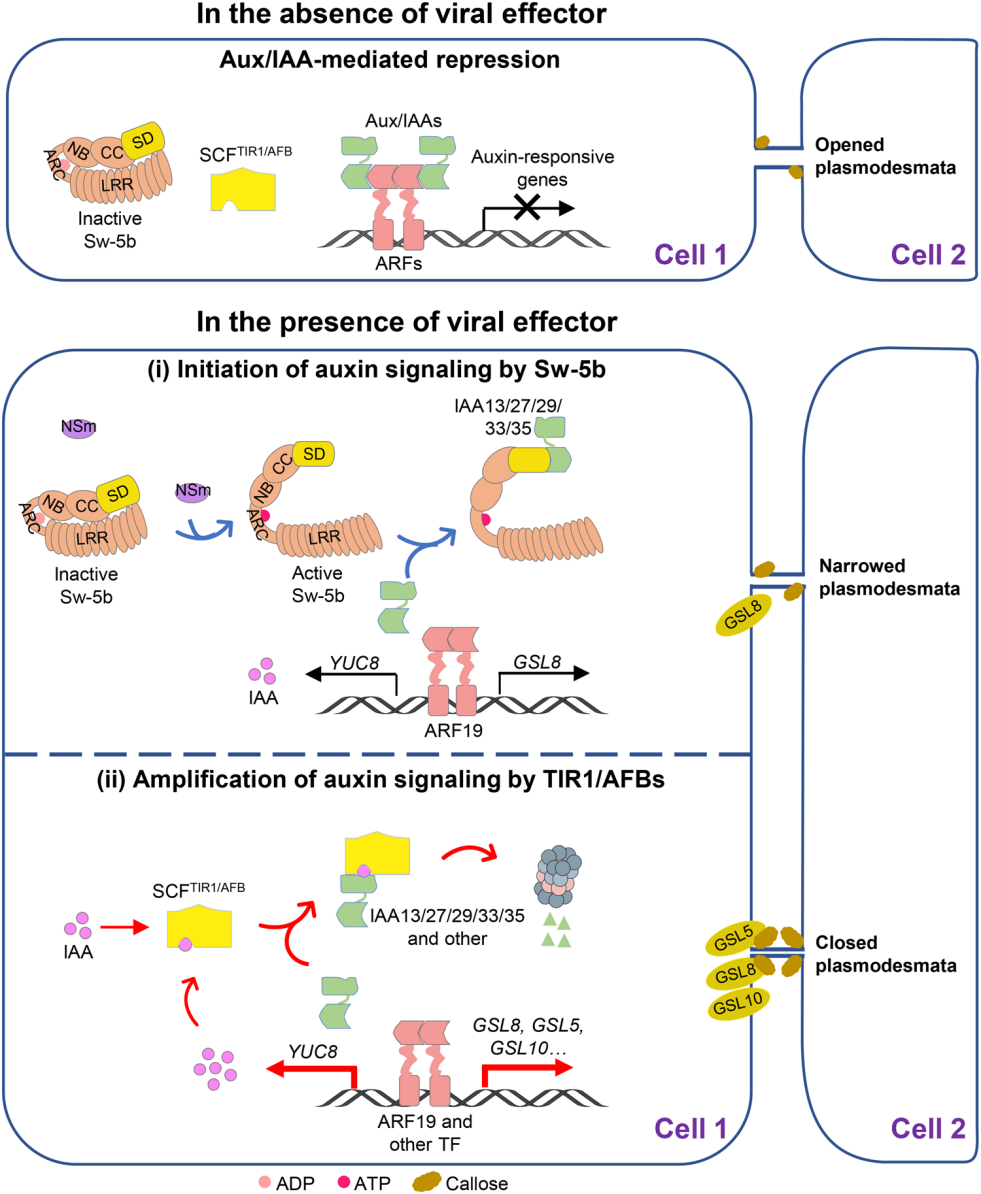
A proposed model that Sw-5b uses auxin central modules to induce callose-mediated antiviral immunity. Under normal condition (top), Sw-5b was inactive. The IAA concentration was low, and Aux/IAA repressors interacted with ARFs and inhibited the transcription of auxin-responsive genes, leading to a normal size exclusion limit of PD. Upon TSWV infection (bottom), Sw-5b NLR recognizes viral effector NSm and undergoes a conformational change from an inactive state to an active state. Then, activated Sw-5b directly interacts with several Aux/IAAs (IAA13, 27, 29, 33, and 35) via SD and depresses the repression on ARF19, thereby activating the transcription of *GSL8* and *YUC8*, inducing partial accumulation of callose and IAA. The plasmodesmal permeability is reduced. Moreover, the increased IAA binds to SCF^TIR1/AFB^ complex and targets more Aux/IAA proteins for degradation by the ubiquitin-26*S* proteasome pathway, releasing ARF19 and other ARFs to activate the transcription of *GSL8*, *GSL5*, and *GSL10*. Massive callose deposition results in PD closure and viral movement blocking, and the increased IAA further forms a feedback circuit to amplify the above responses.

On the basis of our findings, we propose a model in which Sw-5b uses Aux/IAAs-ARF19 module and YUC8-Auxin-TIR1/AFB signaling circuit to induce robust callose deposition and antiviral defense (Fig. 8). In the absence of viral effector NSm, Sw-5b maintains an inactive state. Aux/IAA repressors bind to ARFs and suppress the transcription of auxin-responsive genes. Upon recognition of the viral effector NSm, Sw-5b associates with Aux/IAA proteins (IAA13/27/29/33/35) and depresses the repression of Aux/IAAs on ARF19 to initiate the auxin signaling. ARF19 subsequently activates *YUC8* and *GSL8*, which promotes auxin biosynthesis and callose deposition, respectively. As IAA accumulates, the SCF^TIR1/AFB^ complex further facilitates the degradation of Aux/IAAs, reinforcing ARF19 activity and amplifying the immune response. This cascade ultimately strengthens plasmodesmal callose barriers and effectively blocks viral intercellular movement. Our findings shed light into regulatory mechanism by which plant NLR integrates hormone signaling to induce a robust immunity against pathogens.

## MATERIALS AND METHODS

### Plant materials and growth conditions

The *Sw-5b* transgenic lines of *N. benthamiana* were described previously (*60*). The seeds of tomato cv. IVF1545 carrying *Sw-5b* were provided by J. Li at the Institute of Vegetables and Flowers, Chinese Academy of Agricultural Sciences. The seeds of tomato cv. “Alisa Craig” without *Sw-5b* were provided by H. Zhang at the Institute of Horticulture Science, Shanghai Academy of Agricultural Sciences. *Nbarf19* knockout *N. benthamiana* mutant plants were generated using a CRISPR-Cas9–based technology with the “CRISPR-P 2.0” single guide RNA (sgRNA) design tool as described (*62*). Homozygous T2 lines were used after sequencing validation with specific primers. Primers sequences are provided in table S2.

*N. benthamiana* and tomato seedlings were grown in the greenhouse at 22°C (for VIGS assays) or 25°C (for virus inoculation, protein analysis, and other assays) with a 16/8-hour light/dark photoperiod. Five-/seven-leaf stage *N. benthamiana* plants were used for virus inoculation, protein analysis, and other assays, while three-/five-leaf stage *N. benthamiana* plants were used for VIGS assays.

### Plasmid construction

p2300-Sw-5b, p2300-Sw-5b^D857V^, p2300-YFP-Sw-5b, and p2300-NSm were from a previously described source (*60*). p2300-YFP-SD, pCV-cYFP-SD, p2300-YFP-SD, and pGBKT7-SD were constructed previously (*44*).

For Y2H assays, full-length open reading frame (ORF) of *Sl/NbIAAs* and *Sl/NbARF19* were amplified from *N. benthamiana* or *S. lycopercicum* cDNA and inserted into pGADT7 vector. The coding sequences of *Sl/NbIAA13/27/29/33/35* and *MBP* were amplified to generate pBridge-Sl/NbIAAs, pBridge-Sl/NbIAAs-MBP, and pBridge-Sl/NbIAAs-SD for Y3H assays.

For transient gene expression in *N. benthamiana* leaf cells, the ORFs of *NbGSL5/8/10* and *NbYUC8* were amplified from cDNA and cloned into p2300 and p2300-YFP vector (*21*). The *Sl/NbIAA35* or *GUS* fragments were fused with FLAG tag at C terminus, while *Sl/NbARF19* and *NbARF7* fragments were fused with YFP tag at N terminus, and subcloned into pCambia2300S vector individually. The *Sl/NbARF19* and *Sl/NbIAA35* fragments were fused with cYFP and nYFP individually and subcloned into pCV vector to generate pCV-cYFP-Sl/NbARF19 and pCV-nYFP-Sl/NbIAA35.

The fragment of *Nb/SlARF19* DBD were amplified and inserted into pET28a vector for EMSA assays. For GST pull-down assays, the fragment of *Nb/SlARF19* PB1 domain were amplified and fused with GST tag at N terminus and subcloned into pGEX2TK vector. The ORF of *Sl/NbIAA35* were fused with 3xHA tag, while the fragment of MBP and SD were fused with FLAG tag and cloned into pET28a vector individually.

For VIGS assays, 250 to 300–base pair (bp) fragments of *NbGSL5/8/10* were amplified, and then the fragment was fused by an overlap PCR. The fused fragment was inserted into TRV2 to generate pTRV2-*GSL5/8/10*. The same strategy was adopted to generate pTRV2-*TIR1/AFBs*, pTRV2-*ARF19*, and pTRV2-*YUC8*. For silencing gene by dsRNA-mediated RNAi in agro-infiltrated leaves, the sequences mentioned above and their reverse complementary sequences were subcloned into p2300-intron (mentioned in fig. S4A).

To generate *Nbarf19* knockout mutant *N. benthamiana* lines, two sgRNAs specific for *NbARF19* (Niben101Scf00981g04003) and its allele (Niben101Scf01869g00012) were designed using the online tool CRISPR-P 2.0 (http://cbi.hzau.edu.cn/CRISPR2/) as described (*63*). The sgRNA1-U6t-U6p-sgRNA2 module was amplified with four primers from pCBC-DT1T2 and inserted into pHEE401E-2sgR (*64*).

For dual-luciferase reporter assays, the promoter sequences of *NbGSL5/8/10* and *NbYUC8* was cloned from total DNA of *N. benthamiana* leaf samples and inserted into pGreen II 0800-LUC vector, to generate *NbGSL5/8/10_pro_*: LUC and *NbYUC8_pro_*: LUC, respectively.

All PCR amplifications were conducted with Phanta Max Super-Fidelity DNA Polymerase (Vazyme, Nanjing, China), and all the constructs mentioned above were sequenced before use. Primes used here are listed in table S2.

### Y2H screening and Y2H and Y3H assays

Y2H screening was performed as previously described (*65*). Briefly, the construct pGBKT7-SD was transformed into Y2H-gold yeast cells and mated with the *N. benthamiana* cDNA prey library (generated in Clonetech) at 30°C with 45 rpm shaking for 24 hours. The mated cell cultures were inoculated at 28°C on a selective medium SD/-THL for 5 days. Positive prey clones were then isolated to obtain plasmids for sequencing. Plasmids without frame shift were cotransformed with pGBKT7-SD into Y2H-gold yeast cells for the verification of interaction.

Y2H and Y3H assays were performed as instructed (Clontech, CA, USA). The bait and prey constructs were cotransformed into yeast strain Y2H-Gold. Following growth on SD-LW medium at 30°C for 3 days, transformants were selected on SD medium lacking leucine, tryptophan, histidine, and adenine (SD/-LWHA). Yeast transformed harbored BD-P53 and AD-SV40T was used as a positive control. For the Y3H assays, the Sl/NbIAA35 CDS were fused to the GAL4-binding domain, and the fragments of SD were inserted downstream of the *MET17* promoter in pBridge. The constructs were cotransformed into yeast strain Y2H-Gold and screened on LWHAM medium. Methionine was used to induce the *MET17* promoter for the expression of SD.

### Confocal laser scanning microscopy

The BiFc assays were performed in the leaves of *N. benthamiana* plants. Suspensions of *Agrobacterium tumefaciens* GV3101 transformed with appropriate constructs were adjusted to optical density at 600 nm = 0.5 in MES medium [50 mM MgCl_2_, 50 mM MES, and 200 μM acetosyringone (pH 5.7)] before infiltration. After infiltration, the plants were kept in the greenhouse for 24 hours before analysis. Images of individual samples were captured using Zeiss LSM980 Airyscan2 system (Carl Zeiss AG, Jena, German) with a 20×/0.8 objective.

To examine the cell-to-cell movement efficiency of NSm, the pZK358 (mCherry-HDEL//NSm-GFP) (*45*) was coexpressed with other constructs in *N. benthamiana* leaf cells. Samples were harvested 22 hpi and captured under a 20×/0.8 objective.

To observe the subcellular localization of ARF7/ARF19, *agrobacteria* containing YFP-tagged constructs were transformed into *N. benthamiana* leaves individually. H2B-RFP was served as the nuclear maker, respectively. Images were taken 24 hpi under a 40×/1.10 water objective.

To investigate the plasmodesmal permeability, A Drop-ANd-See assay was performed as described (*66*). Briefly, 1 mM CFDA (MCE, NJ, USA) was loaded as a 1-μl droplet on the upper surface of *N. benthamiana* leaf for 10 min, followed by a confocal imaging of the lower surface of the leaf under a 10×/0.8 objective.

RFP signal was excited at 568 nm, while the emission was captured at 600 to 620 nm. YFP signal was excited at 488 nm, while the range of emission was 497 to 520 nm.

### Western blot and Co-IP assays

Western blot and Co-IP assays were performed as described (*41*). *A. tumefaciens* GV3101 strains carrying the expression constructs were infiltrated into *N. benthamiana* leaves. Leaf tissues (1 g per sample) were harvested, homogenized in 2 ml of extraction buffer [10% glycerol, 25 mM tris-HCl (pH 7.5), 1 mM EDTA, 150 mM NaCl, 10 mM dithiothreitol (DTT), 2% polyvinylpolypyrrolidone, 0.5% Triton X-100, and protease inhibitors cocktail], and centrifuged at 20,000*g* for 20 min at 4°C. For Western blot assays, 60 μl of supernatant was mixed with 30 μl of SDS-loading buffer [150 mM tris-HCl (pH 6.8), 30% glycerol, 6% SDS, 0.3% bromophenol blue, and 300 mM DTT], boiled for 10 min, and separated by SDS–polyacrylamide gel electrophoresis (SDS-PAGE). For Co-IP assays, 1 ml of supernatant was mixed with 30 μl of GFP-trap agarose beads (KT Life Technology, Shenzhen, China) and incubated for 2 hours at 4°C. After centrifugation at 3000*g* for 3 min, the beads were washed four times with IP buffer [50 mM tris-HCl (pH 8.5), 100 mM NaCl, and 1 mM EDTA] and resuspended in 30 μl of SDS-loading buffer. Mixtures were boiled for 10 min, separated by SDS-PAGE, and then transferred to polyvinylidene difluoride membranes (Roche, Shanghai, China). The blots were detected using primary antibodies [anti–FLAG–horseradish peroxidase (HRP) (Sigma-Aldrich, 1:10,000), anti-YFP (1:20,000), anti-NSm (1:5000), or anti-N (1:5000)] and secondary goat anti-rabbit polyclonal antibody (Sigma-Aldrich, 1:10,000) conjugated with HRP. HRP staining was performed with a SuperSignal West Femto (Thermo Fisher Scientific, Waltham, MA, USA). The images were captured by ChemiDoc Imaging System (Bio-Rad, Hercules, CA, USA).

### RNA extraction and qRT-PCR

Total RNA was isolated from *N. benthamiana* and tomato plant leaves using a Total RNA Purification Kit (Tiangen Biotech, Beijing, China) according to the manufacturer’s instructions. First-strand cDNA was synthesized using a HiScript III qRT SuperMix (Vazyme Biotech, Nanjing, China). qRT-PCR was performed using the qPCR SYBR Green Master Mix (Yeasen Biotech, Shanghai, China) on a CFX Connect Real-Time PCR Detection System (Bio-Rad, Hercules, CA, USA). Each treatment was performed with three technical replicates and a minimum of three biological replicates (details in figure legends). *NbActin* and *SlActin* were used as internal controls. Primer sequences used for qRT-PCR are provided in table S2.

### RNA sequencing

RNA isolation was performed as described above. Sequencing libraries were generated using the NEBNext UltraTM RNA Library Prep Kit for Illumina as instructed [New England Biolabs (NEB), MA, USA] followed by addition of index codes. Clustering of the index-coded samples was performed using the TruSeq PE Cluster Kit v3-cBot-HS as instructed (Illumina, San Diego, CA, USA) and then sequenced on an Illumina Novaseq platform to generate ~150-bp paired-end reads. Index of the reference genome (https://btiscience.org/our-research/research-facilities/research-resources/nicotiana-benthamiana/) was built using the Hisat2 v2.0.5 software, and the resulting paired-end clean reads were aligned to the reference genome using the Hisat2 v2.0.5 software. The mapped reads from each sample were assembled using the StringTie software (v1.3.3b) through a reference-based approach. The expression level of each gene was output as fragments per kilobase of exon per million fragments mapped values. Groups of three biological replicates were combined, and the DEGs were identified using DEseq2. DEGs were identified using a cutoff of |log_2_ FC| > 1.5 and *P* value <0.05.

### Callose staining and quantification

PD callose staining was performed as described (*39*) with simply modifications. In brief, treated leaf samples were infiltrated with 1% (w/v) aniline blue solution (Sangon Biotech, Shanghai, China) containing 0.01 M K_3_PO_4_ (pH 12) for 0.5 to 1 hour before imaging. Images were taken by Zeiss LSM980 Airyscan2 system with a 40×/1.10 water objective. The 405-nm diode laser was selected for excitation and the 475- to 525-nm emission filter for aniline blue fluorescence. PDLP5-YFP was served as a PD marker with 488-nm excitation/500- to 550-nm emission. Callose quantification was performed using ImageJ software with the calloseQuant plugin as described before (*67*).

### Hormone measurement

The content of IAA was determined through LC-MS/MS. Fresh leaf tissues (100 mg per sample) were ground into powder using liquid nitrogen in a 2-ml centrifuge tube, and 1.5 ml extraction buffer (methanol:water:formic acid, 15:4:1) was added. The crude extract was fully vortexed, sonicated for 20 min, and centrifuged at 12,000 rpm for 10 min at 4°C. The supernatant was transferred into a new 1.5-ml tube and dried through evaporation under nitrogen gas stream. The dried powder was redissolved in methanol, and the solution was filtered through a 0.22-μm filter. Each sample was injected into a Xevo TQ-XS Triple Quadrupole Mass Spectrometer (Waters, Milford, MA, USA) equipped with an electrospray ionization ion source and an HSS T3 C18 column (1.8 μm, 2.1 mm by 100 mm).

### Generation of *Nbarf19* knockout *N. benthamiana* lines

The CRISPR-Cas9–based gene editing methods was described previously (*68*). Briefly, leaf samples were harvested from 8-week-old *N. benthamiana* plants and sterilized with 70% ethanol followed by 10% sodium hypochlorite solution and then rinsed three times with sterile water. Disks were cut from treated leaf samples and incubated in a culture of *A. tumefaciens* carrying construct for 10 min. Subsequently, the leaf disks were cultured on a cocultivation medium for 2 days at 25°C in the dark and transferred onto a shoot regeneration medium for 3 weeks at 25°C with a 16/8-hour light/dark photoperiod. The regenerated shoots were collected and transferred onto an MS rooting medium without hormones for 20 to 30 days. When the roots reached a length of 3 to 5 cm, the seeds were transferred into soil and grown in a greenhouse. Genomic DNA was extracted from regenerated plants, and the targeted sites of *NbARF19* and its alleles were amplified and sequenced to confirm that all the genes were edited successfully.

### In vitro protein expression, competitive GST pull down, and EMSA

Constructs were individually transformed into *E. coli* Rosetta strain to express and purify recombinant proteins as previously described (*61*). For His-tagged proteins, crude extracts were incubated with Ni–nitrilotriacetic acid Agarose Resin (Yeasen Biotech, Shanghai, China) for purification according to the manufacturer’s instructions.

For competition GST pull-down assays, crude extracts of GST-ARF19 or GST were individually incubated with 30 μl of glutathione-agarose resin (GenScript, Nanjing, China) in a 1.5-ml tube for 1 hour at 4°C and spun to collect the pellets. Purified His-IAA35-HA together with increasing amount of His-SD-FLAG or His-MBP-FLAG were added into the tube and incubated for another 1 hour at 4°C. After centrifugation, the beads were washed four times with wash buffer [50 mM tris-HCl (pH 8.5), 100 mM NaCl, and 1 mM EDTA]. Protein samples were separated by SDS-PAGE and detected with anti-GST (rabbit, 1: 5,000), anti–FLAG-HRP (Sigma-Aldrich, 1:10,000), and anti-HA (Abcam, mouse, 1:20,000).

For EMSA, DNA probes of *NbGSL8* and *NbYUC8* promoter were labeled with Alexa Flour 660 (Thermo Fisher Scientific, Shanghai, China). The purified His-NbARF19 DBD was transferred into a dialysis membrane (MW:14,000, Biosharp, Beijing, China) and dialyzed in 1× phosphate-buffered saline for 6 hours at 4°C to remove imidazole. Recombinant protein was incubated with labeled probes at room temperature for 20 min, and unlabeled probes were used as a competitor to examine the specificity of binding. Protein-DNA complexes were separated by electrophoresis on an agarose gel, and the fluorescence signal was detected by Odyssey DLx Imaging System (LI-COR, Nebraska, USA).

### Virus inoculation and IAA treatment

TSWV inoculation was performed as described (*21*). TSWV-infected leaf samples were harvested and ground in 1 × phosphate buffer (137 mM NaCl, 2.7 mM KCl, 10 mM Na_2_HPO_4_, and 2 mM KH_2_PO_4_, pH 7.4). The crude extracts were rub-inoculated onto the leaves of tomato or *N. benthamiana* plants. The viral abundance was detected by Western blot using anti-TSWV N antibody.

To assess the antiviral capacity by IAA treatment, 4-week-old *N. benthamiana* or 6-week-old tomato plant leaves were sprayed with 25 μM IAA (Yeasen Biotech, Shanghai, China) 1 day before TSWV inoculation. At 2 dpi, the plants were sprayed twice of IAA with a 3-day interval.

### VIGS and dsRNA-mediated RNAi

For VIGS assays, the leaves of 3-week-old *N. benthamiana* or tomato plants were infiltrated with *agrobacterium* culture harboring pTRV1 and various pTRV2 derivatives. The plants were grown in chamber for 3 weeks, and the systemic leaves were used for virus inoculation or other experiments.

For dsRNA-mediated RNAi assays, *agrobacterium* culture carrying dsRNA constructs was infiltrated into *N. benthamiana* leaves. At 12 hpi, the infiltrated leaves were used for qRT-PCR, transient gene expression, TSWV inoculation, or other experiments.

### Dual-luciferase reporter assay

*N. benthamiana* leaves were transformed with *Agrobacteria* containing the effectors and reporters mentioned in the figures. Leaf samples were collected at 22 hpi followed by incubation with d-Luciferin solution (Yeasen Biotech, Shanghai, China) in dark for 5 min. Images were taken under a low light–cooled charge-coupled device imaging system (VILBER, Ile-de-France, France). The relative LUC activities were quantified using ImageJ software according to gray levels.

### Phylogenetic analysis

The homologous proteins were searched in NCBI GenBank (https://ncbi.nlm.nih.gov/genbank/) and Sol Genomics Network (https://solgenomics.net/). Sequence alignment was conducted in MEGA 7 by ClustalW with default parameters. Neighbor-joining trees were constructed using Poisson model with bootstrap values of 1000 replicates.

### Statistical analysis

Callose intensity, relative LUC activity, and protein level quantification were performed in ImageJ software. All statistical analysis was performed using two-tailed Student’s *t* test (means comparison between two groups), one-way analysis of variance (ANOVA) (means comparison among at least three groups with one independent variable), or two-way ANOVA (means comparison among at least three groups with two independent variables) in GraphPad Prism v9 and shown in figures and figure legends.

## References

[1] J. D. G. Jones, J. L. Dangl, The plant immune system. Nature 444, 323–329 (2006). 17108957 10.1038/nature05286

[2] J. D. G. Jones, R. E. Vance, J. L. Dangl, Intracellular innate immune surveillance devices in plants and animals. Science 354, aaf6395 (2016). 27934708 10.1126/science.aaf6395

[3] J. D. G. Jones, B. J. Staskawicz, J. L. Dangl, The plant immune system: From discovery to deployment. Cell 187, 2095–2116 (2024). 38670067 10.1016/j.cell.2024.03.045

[4] T. A. DeFalco, C. Zipfel, Molecular mechanisms of early plant pattern-triggered immune signaling. Mol. Cell 81, 3449–3467 (2021). 34403694 10.1016/j.molcel.2021.07.029

[5] J.-M. Zhou, Y. Zhang, Plant immunity: Danger perception and signaling. Cell 181, 978–989 (2020). 32442407 10.1016/j.cell.2020.04.028

[6] Z. Guo, Y. Li, S.-W. Ding, Small RNA-based antimicrobial immunity. Nat. Rev. Immunol. 19, 31–44 (2019). 30301972 10.1038/s41577-018-0071-x

[7] D. C. Baulcombe, An RNA world. Annu. Rev. Plant Biol. 74, 1–20 (2023). 36542757 10.1146/annurev-arplant-070622-021021

[8] T. Boller, S. Y. He, Innate immunity in plants: An arms race between pattern recognition receptors in plants and effectors in microbial pathogens. Science 324, 742–744 (2009). 19423812 10.1126/science.1171647 PMC2729760

[9] T. Y. Toruño, I. Stergiopoulos, G. Coaker, Plant-pathogen effectors: Cellular probes interfering with plant defenses in spatial and temporal manners. Annu. Rev. Phytopathol. 54, 419–441 (2016). 27359369 10.1146/annurev-phyto-080615-100204 PMC5283857

[10] Y. Wang, R. N. Pruitt, T. Nürnberger, Y. Wang, Evasion of plant immunity by microbial pathogens. Nat. Rev. Microbiol. 20, 449–464 (2022). 35296800 10.1038/s41579-022-00710-3

[11] H. Cui, K. Tsuda, J. E. Parker, Effector-triggered immunity: From pathogen perception to robust defense. Annu. Rev. Plant Biol. 66, 487–511 (2015). 25494461 10.1146/annurev-arplant-050213-040012

[12] W. D. Teale, I. A. Paponov, K. Palme, Auxin in action: Signalling, transport and the control of plant growth and development. Nat. Rev. Mol. Cell Biol. 7, 847–859 (2006). 16990790 10.1038/nrm2020

[13] W. M. Gray, S. Kepinski, D. Rouse, O. Leyser, M. Estelle, Auxin regulates SCF(TIR1)-dependent degradation of AUX/IAA proteins. Nature 414, 271–276 (2001). 11713520 10.1038/35104500

[14] X. Tan, L. I. Calderon-Villalobos, M. Sharon, C. Zheng, C. V. Robinson, M. Estelle, N. Zheng, Mechanism of auxin perception by the TIR1 ubiquitin ligase. Nature 446, 640–645 (2007). 17410169 10.1038/nature05731

[15] M. Salehin, R. Bagchi, M. Estelle, SCF^TIR1/AFB^-based auxin perception: Mechanism and role in plant growth and development. Plant Cell 27, 9–19 (2015). 25604443 10.1105/tpc.114.133744 PMC4330579

[16] F. Llorente, P. Muskett, A. Sánchez-Vallet, G. López, B. Ramos, C. Sánchez-Rodríguez, L. Jordá, J. Parker, A. Molina, Repression of the auxin response pathway increases *Arabidopsis* susceptibility to necrotrophic fungi. Mol. Plant 1, 496–509 (2008). 19825556 10.1093/mp/ssn025

[17] L. Qi, J. Yan, Y. Li, H. Jiang, J. Sun, Q. Chen, H. Li, J. Chu, C. Yan, X. Sun, Y. Yu, C. Li, C. Li, *Arabidopsis thaliana* plants differentially modulate auxin biosynthesis and transport during defense responses to the necrotrophic pathogen *Alternaria brassicicola*. New Phytol. 195, 872–882 (2012). 22731664 10.1111/j.1469-8137.2012.04208.x

[18] L. Qiao, L. Zheng, C. Sheng, H. Zhao, H. Jin, D. Niu, Rice siR109944 suppresses plant immunity to sheath blight and impacts multiple agronomic traits by affecting auxin homeostasis. Plant J. 102, 948–964 (2020). 31923320 10.1111/tpj.14677

[19] L. Eshraghi, J. P. Anderson, N. Aryamanesh, J. A. McComb, B. Shearer, G. S. J. E. Hardy, Suppression of the auxin response pathway enhances susceptibility to *Phytophthora cinnamomi* while phosphite-mediated resistance stimulates the auxin signalling pathway. BMC Plant Biol. 14, 68 (2014). 24649892 10.1186/1471-2229-14-68 PMC3999932

[20] Q. Qin, G. Li, L. Jin, Y. Huang, Y. Wang, C. Wei, Z. Xu, Z. Yang, H. Wang, Y. Li, Auxin response factors (ARFs) differentially regulate rice antiviral immune response against rice dwarf virus. PLOS Pathog. 16, e1009118 (2020). 33264360 10.1371/journal.ppat.1009118 PMC7735678

[21] J. Chen, Y. Zhao, X. Luo, H. Hong, T. Yang, S. Huang, C. Wang, H. Chen, X. Qian, M. Feng, Z. Chen, Y. Dong, Z. Ma, J. Li, M. Zhu, S. Y. He, S. P. Dinesh-Kumar, X. Tao, NLR surveillance of pathogen interference with hormone receptors induces immunity. Nature 613, 145–152 (2023). 36517600 10.1038/s41586-022-05529-9

[22] S. Padmanabhan Meenu, P. Goregaoker Sameer, S. Golem, H. Shiferaw, N. Culver James, Interaction of the tobacco mosaic virus replicase protein with the Aux/IAA protein PAP1/IAA26 is associated with disease development. J. Virol. 79, 2549–2558 (2005). 15681455 10.1128/JVI.79.4.2549-2558.2005 PMC546588

[23] L. Jin, Q. Qin, Y. Wang, Y. Pu, L. Liu, X. Wen, S. Ji, J. Wu, C. Wei, B. Ding, Y. Li, Rice dwarf virus P2 protein hijacks auxin signaling by directly targeting the rice OsIAA10 protein, enhancing viral infection and disease development. PLOS Pathog. 12, e1005847 (2016). 27606959 10.1371/journal.ppat.1005847 PMC5015840

[24] H. Zhang, L. Li, Y. He, Q. Qin, C. Chen, Z. Wei, X. Tan, K. Xie, R. Zhang, G. Hong, J. Li, J. Li, C. Yan, F. Yan, Y. Li, J. Chen, Z. Sun, Distinct modes of manipulation of rice auxin response factor OsARF17 by different plant RNA viruses for infection. Proc. Natl. Acad. Sci. U. S. A. 117, 9112–9121 (2020). 32253321 10.1073/pnas.1918254117 PMC7183187

[25] E. Evangelisti, B. Govetto, N. Minet-Kebdani, M.-L. Kuhn, A. Attard, M. Ponchet, F. Panabières, M. Gourgues, The *Phytophthora parasitica* RXLR effector penetration-specific effector 1 favours *Arabidopsis thaliana* infection by interfering with auxin physiology. New Phytol. 199, 476–489 (2013). 23594295 10.1111/nph.12270

[26] Y. Zhao, Y. He, X. Chen, N. Li, T. Yang, T. Hu, S. Duan, X. Luo, L. Jiang, X. Chen, X. Tao, J. Chen, Different viral effectors hijack TCP17, a key transcription factor for host Auxin synthesis, to promote viral infection. PLOS Pathog. 20, e1012510 (2024). 39208401 10.1371/journal.ppat.1012510 PMC11389919

[27] F. Lopez-Moya, N. Escudero, E. A. Zavala-Gonzalez, D. Esteve-Bruna, M. A. Blázquez, D. Alabadí, L. V. Lopez-Llorca, Induction of auxin biosynthesis and *WOX5* repression mediate changes in root development in *Arabidopsis* exposed to chitosan. Sci. Rep. 7, 16813 (2017). 29196703 10.1038/s41598-017-16874-5 PMC5711845

[28] B. P. M. Ngou, P. Ding, J. D. G. Jones, Thirty years of resistance: Zig-zag through the plant immune system. Plant Cell 34, 1447–1478 (2022). 35167697 10.1093/plcell/koac041 PMC9048904

[29] L. German, R. Yeshvekar, Y. Benitez-Alfonso, Callose metabolism and the regulation of cell walls and plasmodesmata during plant mutualistic and pathogenic interactions. Plant Cell Environ. 46, 391–404 (2023). 36478232 10.1111/pce.14510 PMC10107507

[30] E. E. Tee, C. Faulkner, Plasmodesmata and intercellular molecular traffic control. New Phytol. 243, 32–47 (2024). 38494438 10.1111/nph.19666

[31] W. J. Lucas, Plant viral movement proteins: Agents for cell-to-cell trafficking of viral genomes. Virology 344, 169–184 (2006). 16364748 10.1016/j.virol.2005.09.026

[32] V. A. Iglesias, F. Meins Jr., Movement of plant viruses is delayed in a β-1,3-glucanase-deficient mutant showing a reduced plasmodesmatal size exclusion limit and enhanced callose deposition. Plant J. 21, 157–166 (2000). 10743656 10.1046/j.1365-313x.2000.00658.x

[33] W. Li, Y. Zhao, C. Liu, G. Yao, S. Wu, C. Hou, M. Zhang, D. Wang, Callose deposition at plasmodesmata is a critical factor in restricting the cell-to-cell movement of Soybean mosaic virus. Plant Cell Rep. 31, 905–916 (2012). 22200865 10.1007/s00299-011-1211-y

[34] S. W. Wu, R. Kumar, A. B. B. Iswanto, J. Y. Kim, Callose balancing at plasmodesmata. J. Exp. Bot. 69, 5325–5339 (2018). 30165704 10.1093/jxb/ery317

[35] B. Saatian, S. E. Kohalmi, Y. Cui, Localization of *Arabidopsis* glucan synthase-like 5, 8, and 12 to plasmodesmata and the GSL8-dependent role of PDLP5 in regulating plasmodesmal permeability. Plant Signal. Behav. 18, 2164670 (2023). 36645916 10.1080/15592324.2022.2164670 PMC9851254

[36] J. M. Guseman, J. S. Lee, N. L. Bogenschutz, K. M. Peterson, R. E. Virata, B. Xie, M. M. Kanaoka, Z. Hong, K. U. Torii, Dysregulation of cell-to-cell connectivity and stomatal patterning by loss-of-function mutation in *Arabidopsis CHORUS* (*GLUCAN SYNTHASE-LIKE 8*). Development 137, 1731–1741 (2010). 20430748 10.1242/dev.049197

[37] A. Töller, L. Brownfield, C. Neu, D. Twell, P. Schulze-Lefert, Dual function of *Arabidopsis* glucan synthase-like genes GSL8 and GSL10 in male gametophyte development and plant growth. Plant J. 54, 911–923 (2008). 18315544 10.1111/j.1365-313X.2008.03462.x

[38] B. Saatian, R. S. Austin, G. Tian, C. Chen, V. Nguyen, S. E. Kohalmi, D. Geelen, Y. Cui, Analysis of a novel mutant allele of GSL8 reveals its key roles in cytokinesis and symplastic trafficking in *Arabidopsis*. BMC Plant Biol. 18, 295 (2018). 30466394 10.1186/s12870-018-1515-y PMC6249969

[39] W. Cui, J.-Y. Lee, *Arabidopsis* callose synthases CalS1/8 regulate plasmodesmal permeability during stress. Nat. Plants 2, 16034 (2016). 27243643 10.1038/nplants.2016.34

[40] J. E. Oliver, A. E. Whitfield, The genus *Tospovirus*: Emerging bunyaviruses that threaten food security. Annu. Rev. Virol. 3, 101–124 (2016). 27578436 10.1146/annurev-virology-100114-055036

[41] M. Zhu, L. Jiang, B. Bai, W. Zhao, X. Chen, J. Li, Y. Liu, Z. Chen, B. Wang, C. Wang, Q. Wu, Q. Shen, S. P. Dinesh-Kumar, X. Tao, The intracellular immune receptor Sw-5b confers broad-spectrum resistance to tospoviruses through recognition of a conserved 21-amino acid viral effector epitope. Plant Cell 29, 2214–2232 (2017). 28814646 10.1105/tpc.17.00180 PMC5635987

[42] S. H. Brommonschenkel, A. Frary, A. Frary, S. D. Tanksley, The broad-spectrum tospovirus resistance gene *Sw-5* of tomato is a homolog of the root-knot nematode resistance gene *Mi*. Mol. Plant Microbe Interact. 13, 1130–1138 (2000). 11043474 10.1094/MPMI.2000.13.10.1130

[43] M. I. Spassova, T. W. Prins, R. T. Folkertsma, R. M. Klein-Lankhorst, J. Hille, R. W. Goldbach, M. Prins, The tomato gene *Sw5* is a member of the coiled coil, nucleotide binding, leucine-rich repeat class of plant resistance genes and confers resistance to TSWV in tobacco. Mol. Breed. 7, 151–161 (2001).

[44] H. Chen, X. Qian, X. Chen, T. Yang, M. Feng, J. Chen, R. Cheng, H. Hong, Y. Zheng, Y. Mei, D. Shen, Y. Xu, M. Zhu, X. S. Ding, X. Tao, Cytoplasmic and nuclear Sw-5b NLR act both independently and synergistically to confer full host defense against tospovirus infection. New Phytol. 231, 2262–2281 (2021). 34096619 10.1111/nph.17535

[45] Z. Feng, F. Xue, M. Xu, X. Chen, W. Zhao, M. J. Garcia-Murria, I. Mingarro, Y. Liu, Y. Huang, L. Jiang, M. Zhu, X. Tao, The ER-membrane transport system is critical for intercellular trafficking of the NSm movement protein and tomato spotted wilt tospovirus. PLOS Pathog. 12, e1005443 (2016). 26863622 10.1371/journal.ppat.1005443 PMC4749231

[46] J.-Y. Lee, X. Wang, W. Cui, R. Sager, S. Modla, K. Czymmek, B. Zybaliov, K. van Wijk, C. Zhang, H. Lu, V. Lakshmanan, A plasmodesmata-localized protein mediates crosstalk between cell-to-cell communication and innate immunity in *Arabidopsis*. Plant Cell 23, 3353–3373 (2011). 21934146 10.1105/tpc.111.087742 PMC3203451

[47] J. Tilsner, W. Nicolas, A. Rosado, E. M. Bayer, Staying tight: Plasmodesmal membrane contact sites and the control of cell-to-cell connectivity in plants. Annu. Rev. Plant Biol. 67, 337–364 (2016). 26905652 10.1146/annurev-arplant-043015-111840

[48] N. Dharmasiri, M. Estelle, Auxin signaling and regulated protein degradation. Trends Plant Sci. 9, 302–308 (2004). 15165562 10.1016/j.tplants.2004.04.003

[49] O. Leyser, Auxin signaling. Plant Physiol. 176, 465–479 (2018). 28818861 10.1104/pp.17.00765 PMC5761761

[50] X. Han, T. K. Hyun, M. Zhang, R. Kumar, E.-j. Koh, B.-H. Kang, W. J. Lucas, J.-Y. Kim, Auxin-callose-mediated plasmodesmal gating is essential for tropic auxin gradient formation and signaling. Dev. Cell 28, 132–146 (2014). 24480642 10.1016/j.devcel.2013.12.008

[51] K.-L. Huang, G.-J. Ma, M.-L. Zhang, H. Xiong, H. Wu, C.-Z. Zhao, C.-S. Liu, H.-X. Jia, L. Chen, J. O. Kjorven, X.-B. Li, F. Ren, The ARF7 and ARF19 transcription factors positively regulate *PHOSPHATE STARVATION RESPONSE1* in *Arabidopsis* roots. Plant Physiol. 178, 413–427 (2018). 30026290 10.1104/pp.17.01713 PMC6130041

[52] X. Kong, C. Zhang, H. Zheng, M. Sun, F. Zhang, M. Zhang, F. Cui, D. Lv, L. Liu, S. Guo, Y. Zhang, X. Yuan, S. Zhao, H. Tian, Z. Ding, Antagonistic interaction between Auxin and SA signaling pathways regulates bacterial infection through lateral root in *Arabidopsis*. Cell Rep. 32, 108060 (2020). 32846118 10.1016/j.celrep.2020.108060

[53] Y. Okushima, P. J. Overvoorde, K. Arima, J. M. Alonso, A. Chan, C. Chang, J. R. Ecker, B. Hughes, A. Lui, D. Nguyen, C. Onodera, H. Quach, A. Smith, G. Yu, A. Theologis, Functional genomic analysis of the *AUXIN RESPONSE FACTOR* gene family members in *Arabidopsis thaliana*: Unique and overlapping functions of ARF7 and ARF19. Plant Cell 17, 444–463 (2005). 15659631 10.1105/tpc.104.028316 PMC548818

[54] D. R. Boer, A. Freire-Rios, W. A. M. van den Berg, T. Saaki, I. W. Manfield, S. Kepinski, I. López-Vidrieo, J. M. Franco-Zorrilla, S. C. de Vries, R. Solano, D. Weijers, M. Coll, Structural basis for DNA binding specificity by the auxin-dependent ARF transcription factors. Cell 156, 577–589 (2014). 24485461 10.1016/j.cell.2013.12.027

[55] Y. Zhao, S. K. Christensen, C. Fankhauser, J. R. Cashman, J. D. Cohen, D. Weigel, J. Chory, A role for flavin monooxygenase-like enzymes in auxin biosynthesis. Science 291, 306–309 (2001). 11209081 10.1126/science.291.5502.306

[56] K. Seong, E. Seo, K. Witek, M. Li, B. Staskawicz, Evolution of NLR resistance genes with noncanonical N-terminal domains in wild tomato species. New Phytol. 227, 1530–1543 (2020). 32344448 10.1111/nph.16628

[57] R. Jiang, J. Li, Z. Tian, J. Du, M. Armstrong, K. Baker, J. Tze-Yin Lim, J. H. Vossen, H. He, L. Portal, J. Zhou, M. Bonierbale, I. Hein, H. Lindqvist-Kreuze, C. Xie, Potato late blight field resistance from QTL dPI09c is conferred by the NB-LRR gene *R8*. J. Exp. Bot. 69, 1545–1555 (2018). 29385612 10.1093/jxb/ery021 PMC5889011

[58] B. N. Kunkel, J. M. B. Johnson, Auxin plays multiple roles during plant–pathogen interactions. Cold Spring Harb. Perspect. Biol. 13, a040022 (2021). 33782029 10.1101/cshperspect.a040022 PMC8411954

[59] H. Zhang, X. Tan, L. Li, Y. He, G. Hong, J. Li, L. Lin, Y. Cheng, F. Yan, J. Chen, Z. Sun, Suppression of auxin signalling promotes rice susceptibility to *Rice black streaked dwarf virus* infection. Mol. Plant Pathol. 20, 1093–1104 (2019). 31250531 10.1111/mpp.12814 PMC6640184

[60] X. Chen, M. Zhu, L. Jiang, W. Zhao, J. Li, J. Wu, C. Li, B. Bai, G. Lu, H. Chen, P. Moffett, X. Tao, A multilayered regulatory mechanism for the autoinhibition and activation of a plant CC-NB-LRR resistance protein with an extra N-terminal domain. New Phytol. 212, 161–175 (2016). 27558751 10.1111/nph.14013

[61] J. Li, H. Huang, M. Zhu, S. Huang, W. Zhang, S. P. Dinesh-Kumar, X. Tao, A plant immune receptor adopts a two-step recognition mechanism to enhance viral effector perception. Mol. Plant 12, 248–262 (2019). 30639751 10.1016/j.molp.2019.01.005

[62] S. Fu, Y. Xu, C. Li, Y. Li, J. Wu, X. Zhou, Rice stripe virus interferes with S-acylation of remorin and induces its autophagic degradation to facilitate virus infection. Mol. Plant 11, 269–287 (2018). 29229567 10.1016/j.molp.2017.11.011

[63] H. Liu, Y. Ding, Y. Zhou, W. Jin, K. Xie, L.-L. Chen, CRISPR-P 2.0: An improved CRISPR-Cas9 tool for genome editing in plants. Mol. Plant 10, 530–532 (2017). 28089950 10.1016/j.molp.2017.01.003

[64] Z.-P. Wang, H.-L. Xing, L. Dong, H.-Y. Zhang, C.-Y. Han, X.-C. Wang, Q.-J. Chen, Egg cell-specific promoter-controlled CRISPR/Cas9 efficiently generates homozygous mutants for multiple target genes in *Arabidopsis* in a single generation. Genome Biol. 16, 144 (2015). 26193878 10.1186/s13059-015-0715-0 PMC4507317

[65] C. Wang, M. Zhu, H. Hong, J. Li, C. Zuo, Y. Zhang, Y. Shi, S. Liu, H. Yu, Y. Yan, J. Chen, L. Shangguan, A. Zhi, R. Chen, K. T. Devendrakumar, X. Tao, A viral effector blocks the turnover of a plant NLR receptor to trigger a robust immune response. EMBO J. 43, 3650–3676 (2024). 39020150 10.1038/s44318-024-00174-6 PMC11377725

[66] W. Cui, X. Wang, J. Y. Lee, Drop-ANd-See: A simple, real-time, and noninvasive technique for assaying plasmodesmal permeability. Methods Mol. Biol. 1217, 149–156 (2015). 25287202 10.1007/978-1-4939-1523-1_10

[67] C. Huang, J. Mutterer, M. Heinlein, “In vivo aniline blue staining and semiautomated quantification of callose deposition at plasmodesmata” in *Plasmodesmata: Methods and Protocols*, Y. Benitez-Alfonso, M. Heinlein, Eds. (Springer US, 2022), pp. 151–165. 10.1007/978-1-0716-2132-5_9 35349138

[68] Q. Wu, C. Tong, Z. Chen, S. Huang, X. Zhao, H. Hong, J. Li, M. Feng, H. Wang, M. Xu, Y. Yan, H. Cui, D. Shen, G. Ai, Y. Xu, J. Li, H. Zhang, C. Huang, Z. Zhang, S. Dong, X. Wang, M. Zhu, S. P. Dinesh-Kumar, X. Tao, NLRs derepress MED10b- and MED7-mediated repression of jasmonate-dependent transcription to activate immunity. Proc. Natl. Acad. Sci. U. S. A. 120, e2302226120 (2023). 37399403 10.1073/pnas.2302226120 PMC10334756

