# Supplementary material for: A plant NLR receptor activates auxin signaling through Aux/IAAs-ARF19 and YUC8-TIR1/AFBs to promote callose-mediated antiviral defense
Source: Sci Adv. 2025 Dec 17;11(51):eaea2275. doi: 10.1126/sciadv.aea2275 (PMC12710714; doi:10.1126/sciadv.aea2275)
Supplement: Supplementary file 1 — Figs. S1 to S26 Legends for tables S1 and S2 [file sciadv.aea2275_sm.pdf]

Science Advances
AAAS

Supplementary Materials for

A plant NLR receptor activates auxin signaling through Aux/IAAs-ARF19 and YUC8-TIR1/AFBs to promote callose-mediated antiviral defense

Tongqing Yang *et al.*

Corresponding author: Xiaorong Tao, taoxiaorong@njau.edu.cn; Yi Xu, xuyiqdpd@njau.edu.cn

Sci. Adv. **11**, eaea2275 (2025)
DOI: 10.1126/sciadv.aea2275

The PDF file includes:

Figs. S1 to S26
Legends for tables S1 and S2

Other Supplementary Material for this manuscript includes the following:

Tables S1 and S2

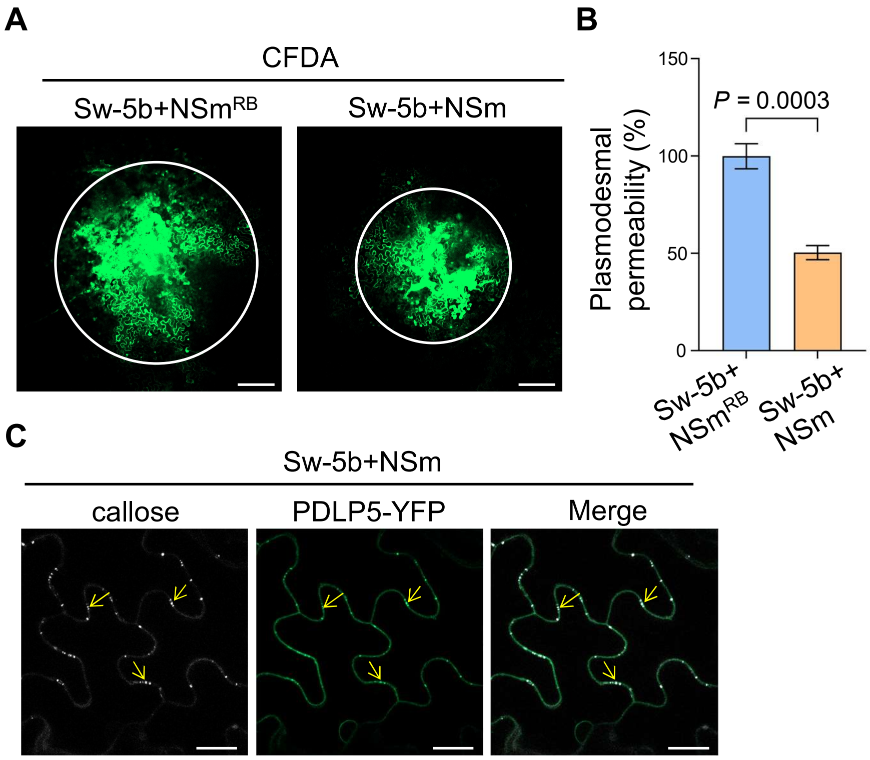

Figure S1. Sw-5b activation reduces plasmodesmal permeability by facilitating callose deposition.

(**A**) A DANS assay showing the diffusion of CF dye in *N. benthamiana* leaves co-expressing Sw-5b with NSm or NSm^{RB}. The dye was loaded onto the leaf surface at 22 hpi for 5 min followed by confocal imaging. Scale bars, 200 µm. (**B**) Relative plasmodesmal permeability in panel (A). Values are means ± SD (two-tailed Student's *t*-test, n = 3 biological replicates). (**C**) Fluorescent images of callose in *N. benthamiana* leaves co-expressing Sw-5b and NSm. PDLP5-YFP was served as a plasmodesmata marker. callose staining was performed at 22 hpi followed by confocal imaging. Scale bars, 20 µm.

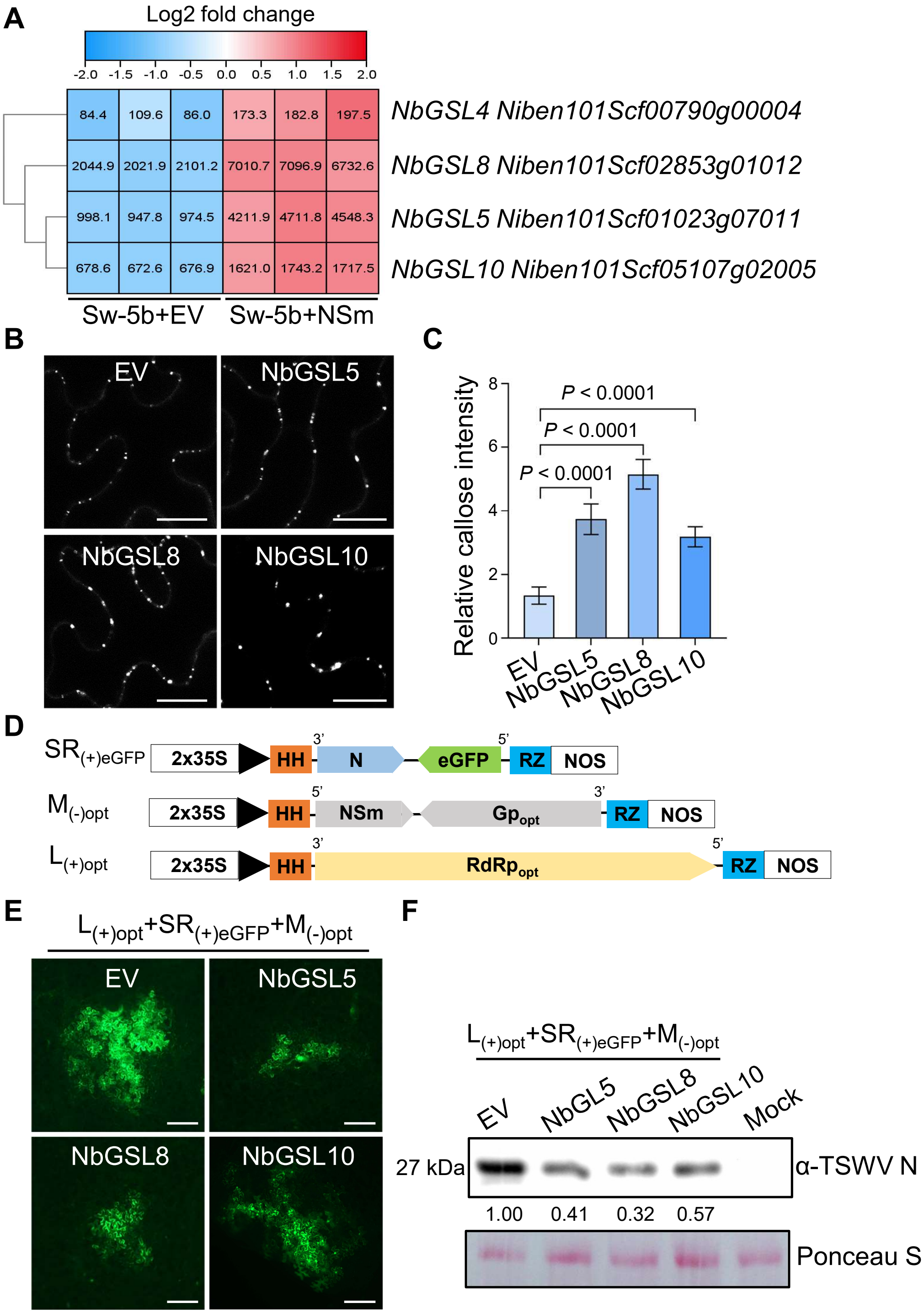

Figure S2. NbGSL5/8/10 positively regulates callose production and host immunity against TSWV.

(**A**) Heatmap showing the upregulated *NbGSL* genes from the RNA-seq data using Sw-5b and NSm co-expressed *N. benthamiana* leaf samples. The heatmap was drew with TBtools software and values in the map represent FPKM (fragments per kilobase million). (**B**) Callose fluorescence in *N. benthamiana* leaves transiently expressing EV, NbGSL5, NbGSL8, or NbGSL10, respectively. Scale bars, 20 µm. (**C**) Quantification of callose intensity in panel (B). Values are means ± SD (two-tailed Student's *t*-test, *n* = 6 biologically independent samples).

(**D**) Schematic representation of TSWV infectious clones carrying a GFP reporter. (**E**) Fluorescent images of TSWV infectious clones co-expressed with either EV, NbGSL5, NbGSL8, or NbGSL10, respectively. Scale bars, 400 μm. (**F**) Immunoblotting analysis of TSWV N accumulation in leaf samples from panel (E). Ponceau-stained bands shows protein loading and protein levels were determined by ImageJ software.

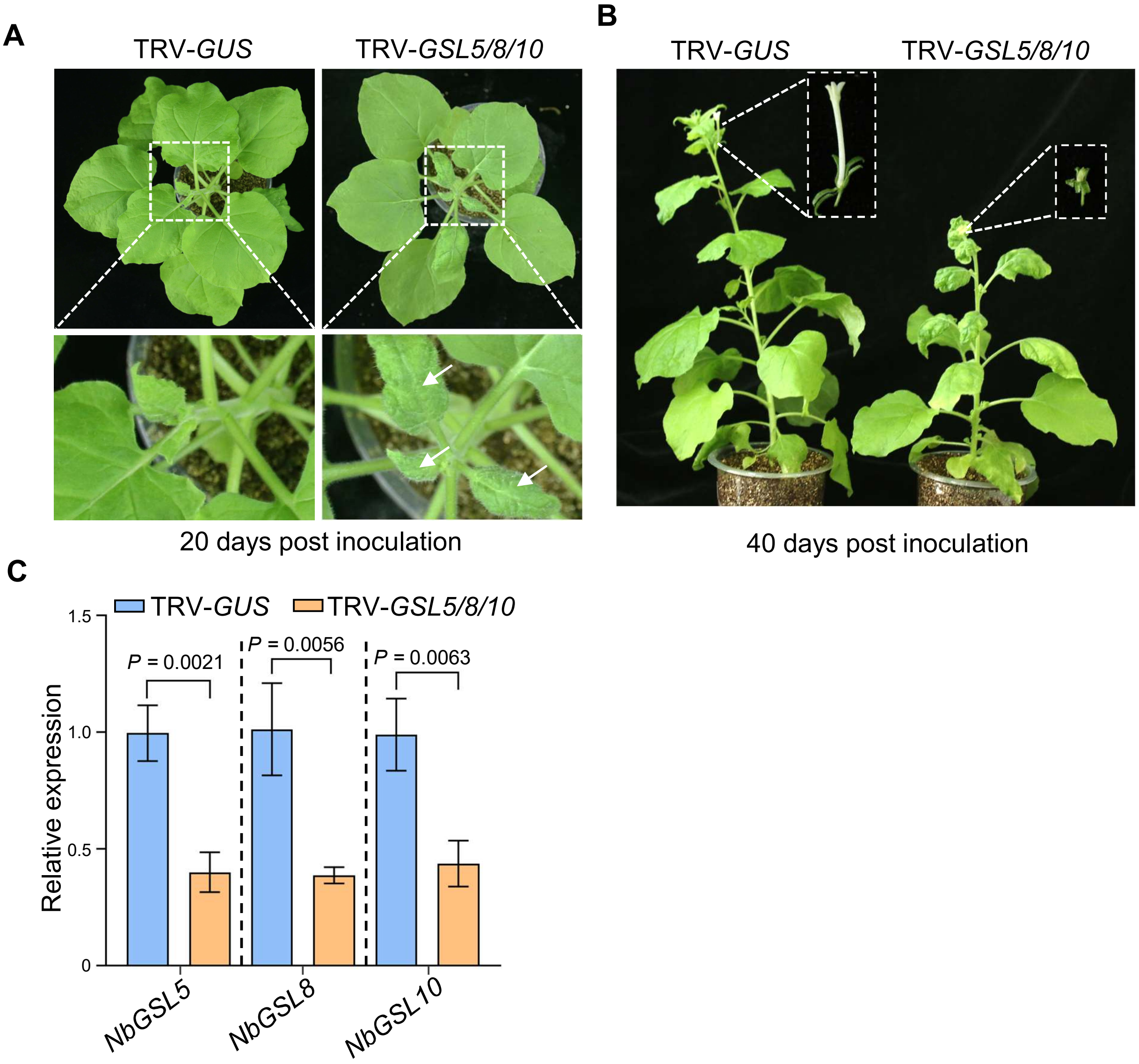

Figure S3. Knockdown of *NbGSL5/8/10* using TRV-based VIGS leads to abnormal phenotype in *N. benthamiana* plants.

(**A** and **B**) Images showing the phenotype of TRV-*GSL5/8/10 N. benthamiana* plants at 20 dpi (A) or 40 dpi (B). (**C**) Relative expression levels of *NbGSL5*, *NbGSL8*, and *NbGSL10* by qRT-PCR. Values are means ± SD (two-tailed Student's *t*-test, *n* = 3 biological replicates).

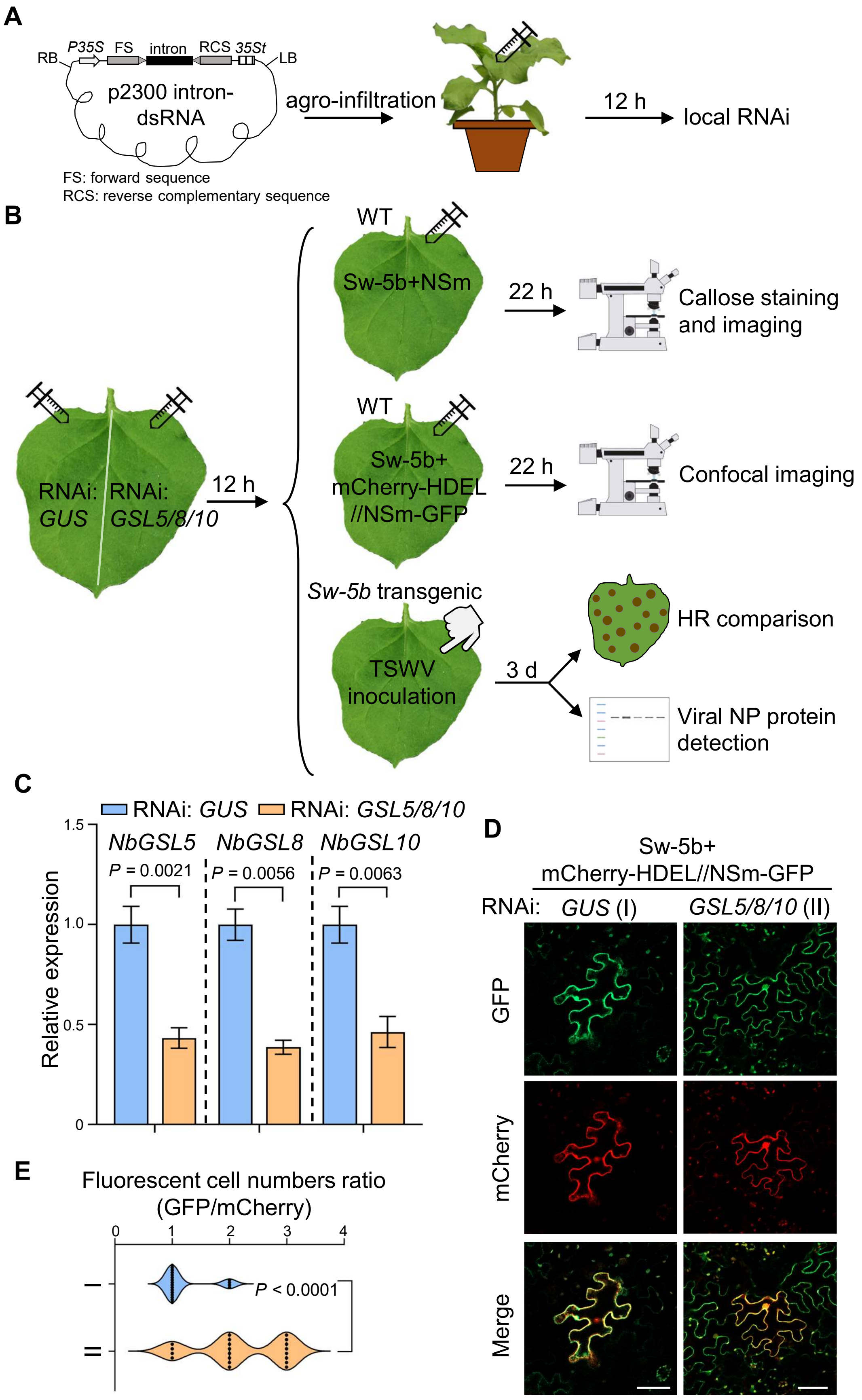

Figure S4. Knockdown of *NbGSL5/8/10* impaired Sw-5b' ability of inhibiting NSm movement.

(**A**) Schematic diagram and flowchart of dsRNA-based gene silencing in *N. benthamiana* local

leaves. (**B**) Schematic diagrams of RNAi of *NbGSL5/8/10* using a half-leaf method in *N. benthamiana* leaves and the flowchart showing experiments performed in Fig. 1 and fig. S4. (**C**) qRT-PCR analysis of *NbGSL5*, *NbGSL8*, and *NbGSL10* expression in leaves treated by RNAi construct. Values are means ± SD (two-tailed Student's *t*-test, *n* = 3 biological replicates). (**D**) Fluorescent images of NSm-GFP in RNAi: *GSL5/8/10*- or RNAi: *GUS*-treated half-leaves from *Sw-5b*-transgenic *N. benthamiana* plants. Scale bars, 20 μm. (**E**) Quantification of the intercellular movement efficiency of NSm-GFP. The average fluorescent cell numbers ratio (GFP/mCherry) from per image were shown on the y axis. *n* = 20.

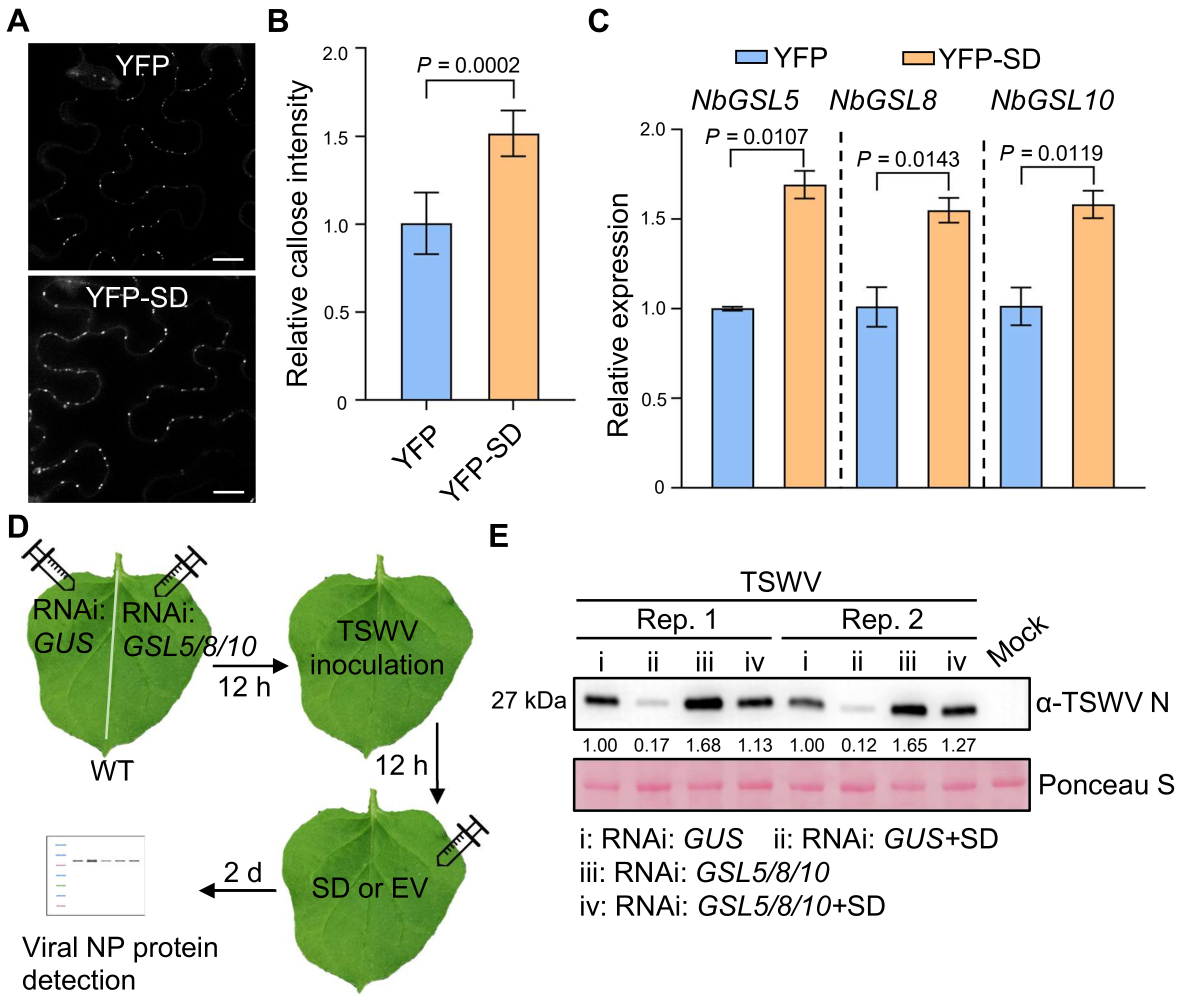

Figure S5. SD-mediated resistance to TSWV partially depends on NbGSL5/8/10.

(**A**) Fluorescent images of plasmodesmal callose in *N. benthamiana* leaves expressing YFP or YFP-SD. Scale bars, 20 µm. (**B**) Quantification of the callose intensity in panel (A). Values are means ± SD of six biologically independent samples. (**C**) qRT-PCR analysis of *NbGSL5/8/10* in YFP- or YFP-SD-expressed leaf samples. Values are means ± SD of three biologically independent replicates. Leaf samples in (A to C) were collected at 22 hpi. Statistical analysis in (B and C) was performed by two-tailed Student's *t*-test. (**D**) Schematic diagrams of RNAi of *NbGSL5/8/10* in *N. benthamiana* leaves and the flowchart showing experiments performed in fig. S5E. (**E**) TSWV accumulation in leaf samples from panel (D) by western blot. Ponceau-stained bands shows protein loading and protein levels were determined by ImageJ software.

A

Number and frequency of NbIAA35 selected in Y2H screening.

Name	Number	Frequency	Self-activation
NbIAA35	3	1.6%	No

B **C**

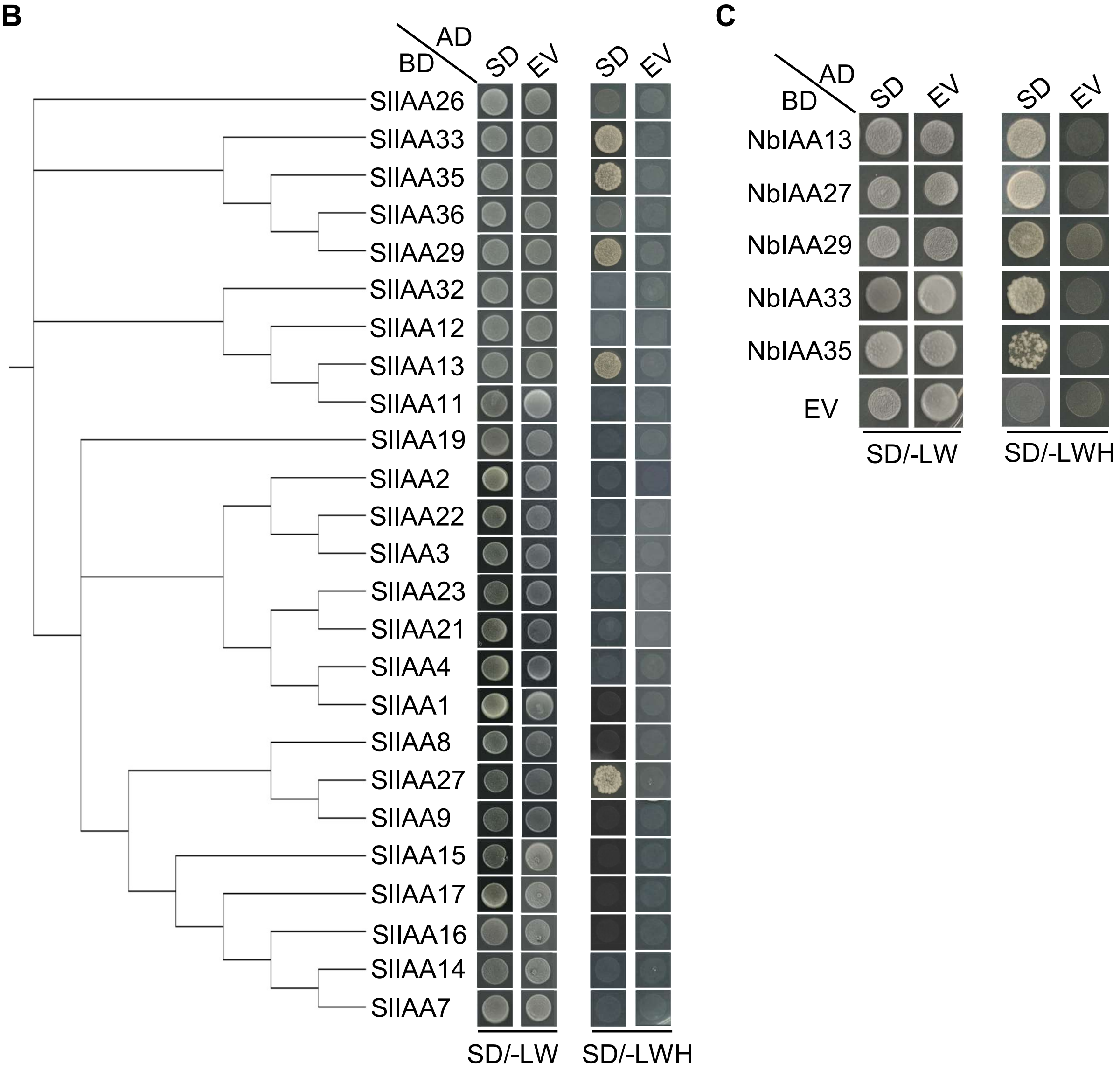

Figure S6. SD interacts with IAA13, IAA27, IAA29, IAA 33, and IAA35.

(**A**) Number and frequency of NbIAA35 identified in Y2H library screening using Sw-5b SD as bait. (**B**) Y2H analysis of the interaction between SD and all 25 IAAs family members from tomato. (**C**) Y2H analysis the interaction between SD and NbIAA13/27/29/33/35. Yeast cells were grown on medium SD/-LW and selected on medium SD/-LWH at 30°C for 4 days. For (B and C), the experiments were repeated three times with similar results.

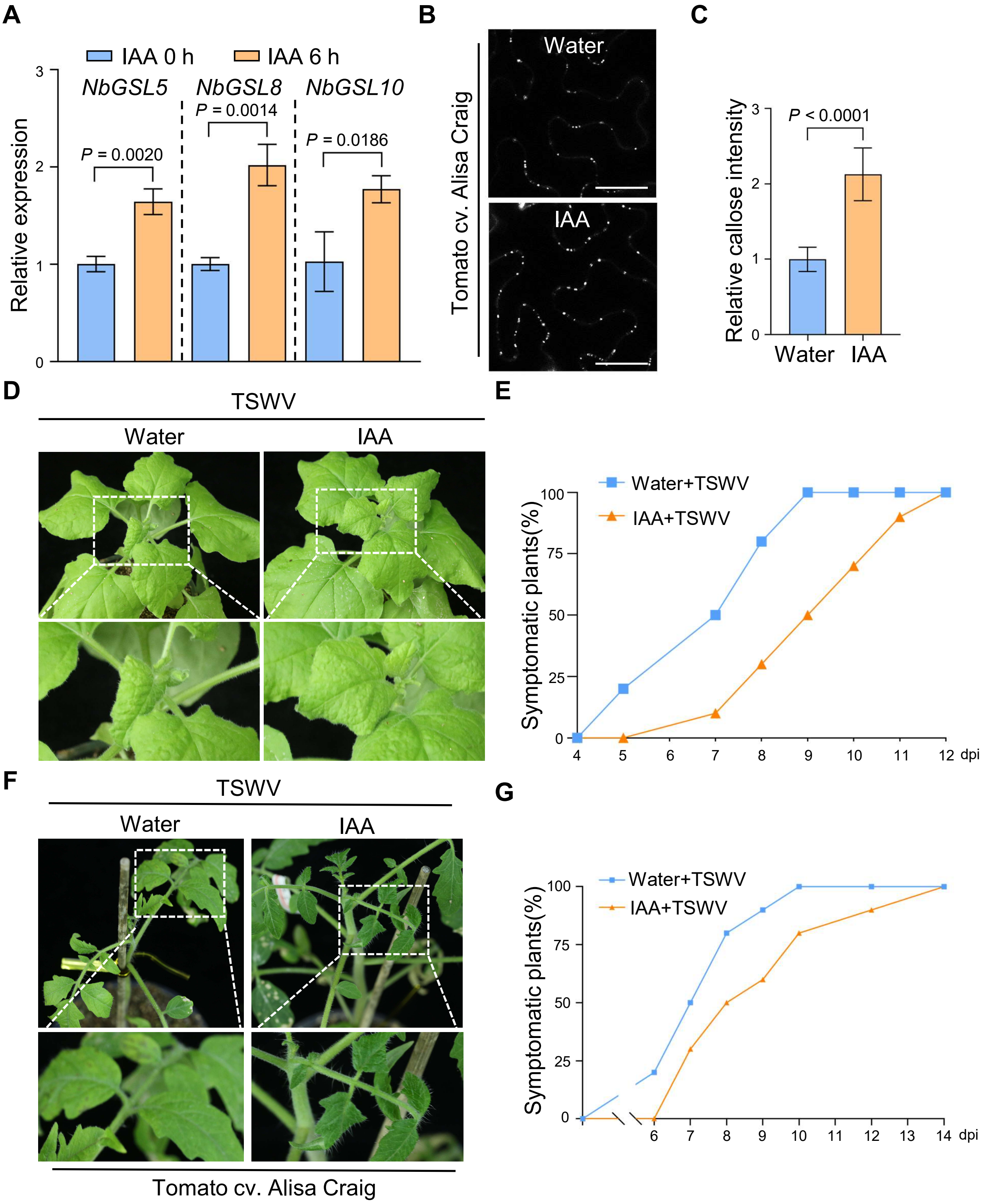

Figure S7. Exogenous IAA induces plasmodesmal callose accumulation and inhibits TSWV systemic infection.

(**A**) qRT-PCR analysis of the expression of *NbGSL5/8/10* in *N. benthamiana* leaves treated with or without IAA. Values are means ± SD of three biological replicates. (**B**) Callose fluorescence at plasmodesmata in water- or IAA-treated tomato leaves. Scale bars, 20 µm. (**C**) Quantification of the callose intensity in panel (B). Values are means ± SD, n = 6 biologically independent samples. (**D**) Symptoms of TSWV-infected *N. benthamiana* plants treated with water or IAA. Photos were taken at 9 dpi. (**E**) Disease development quantification of plants in panel (D). n = 10.

(**F**) Symptoms of TSWV-infected tomato plants treated with water or IAA. Photos were taken at 12 dpi. (**G**) Disease development quantification of plants in panel (F). n = 10. Statistical analysis in (A and C) was performed by two-tailed Student's *t*-test.

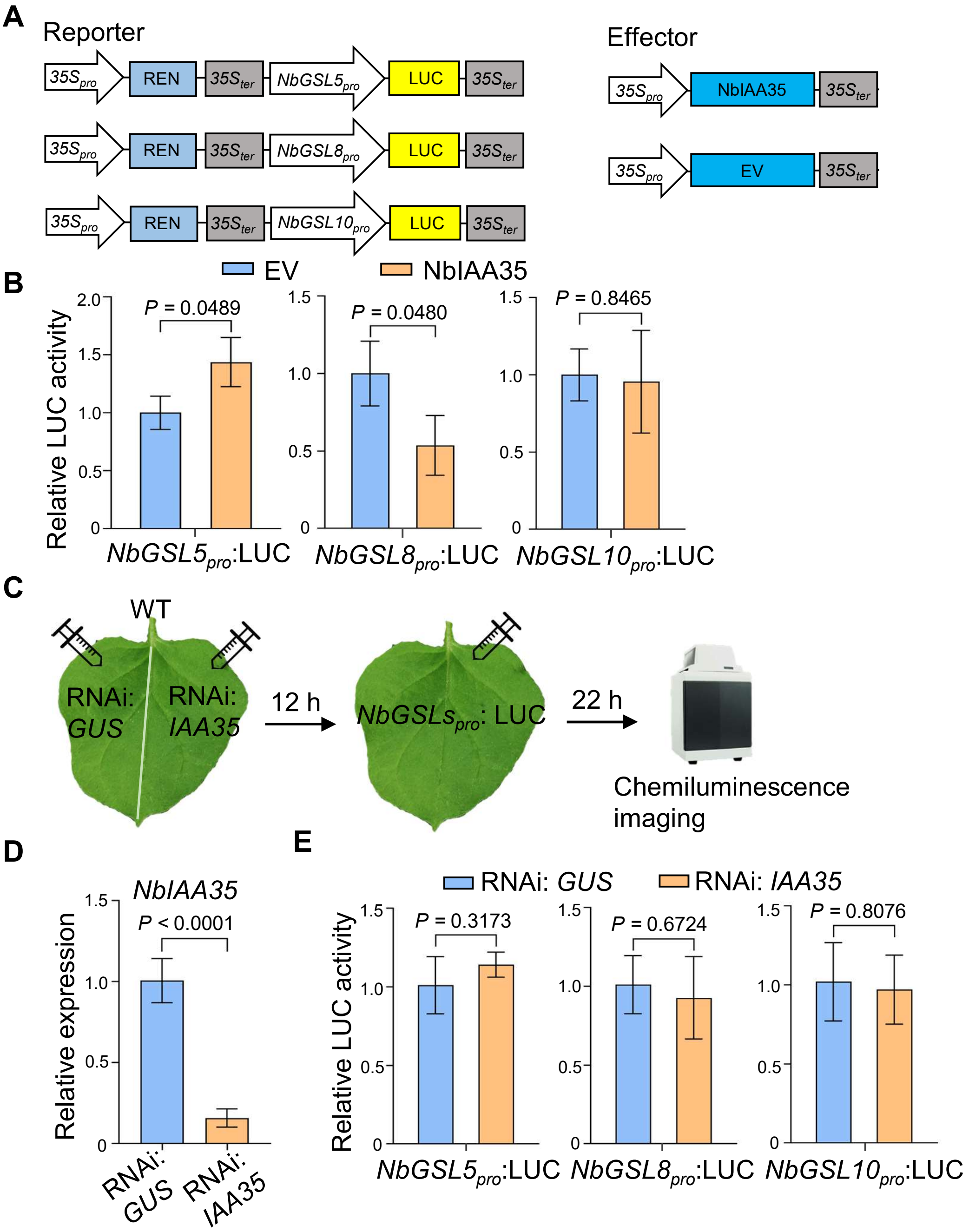

Figure S8. NbIAA35 negatively regulates the transcriptional activity of *NbGSL8* promoter. (**A**) Schematic diagrams of the 'reporter' and 'effector' used in Dual-Luciferase Reporter Assay. (**B**) Relative LUC activities of reporters driven by *NbGSL5/8/10* promoter in *N. benthamiana* leaves expressing NbIAA35 or EV. Values are means ± SD of three biological replicates. (**C**) Schematic diagrams of RNAi of *NbIAA35* using a half-leaf method in *N. benthamiana* leaves and the flowchart showing the experiment performed in fig. S8E. (**D**) qRT-PCR analysis of *NbIAA35* expression level in RNAi: *GUS* or RNAi: *IAA35* leaf samples. Values represent means ± SD, n = 3 biological replicates. (**E**) Relative LUC activities of reporters driven by *NbGSL5/8/10* promoter in *NbIAA35*-silenced or non-silenced *N. benthamiana* leaf samples. Values are means ± SD, n = 3 biological replicates. Data in (B, D, and E) were analyzed by two-tailed Student's t-test.

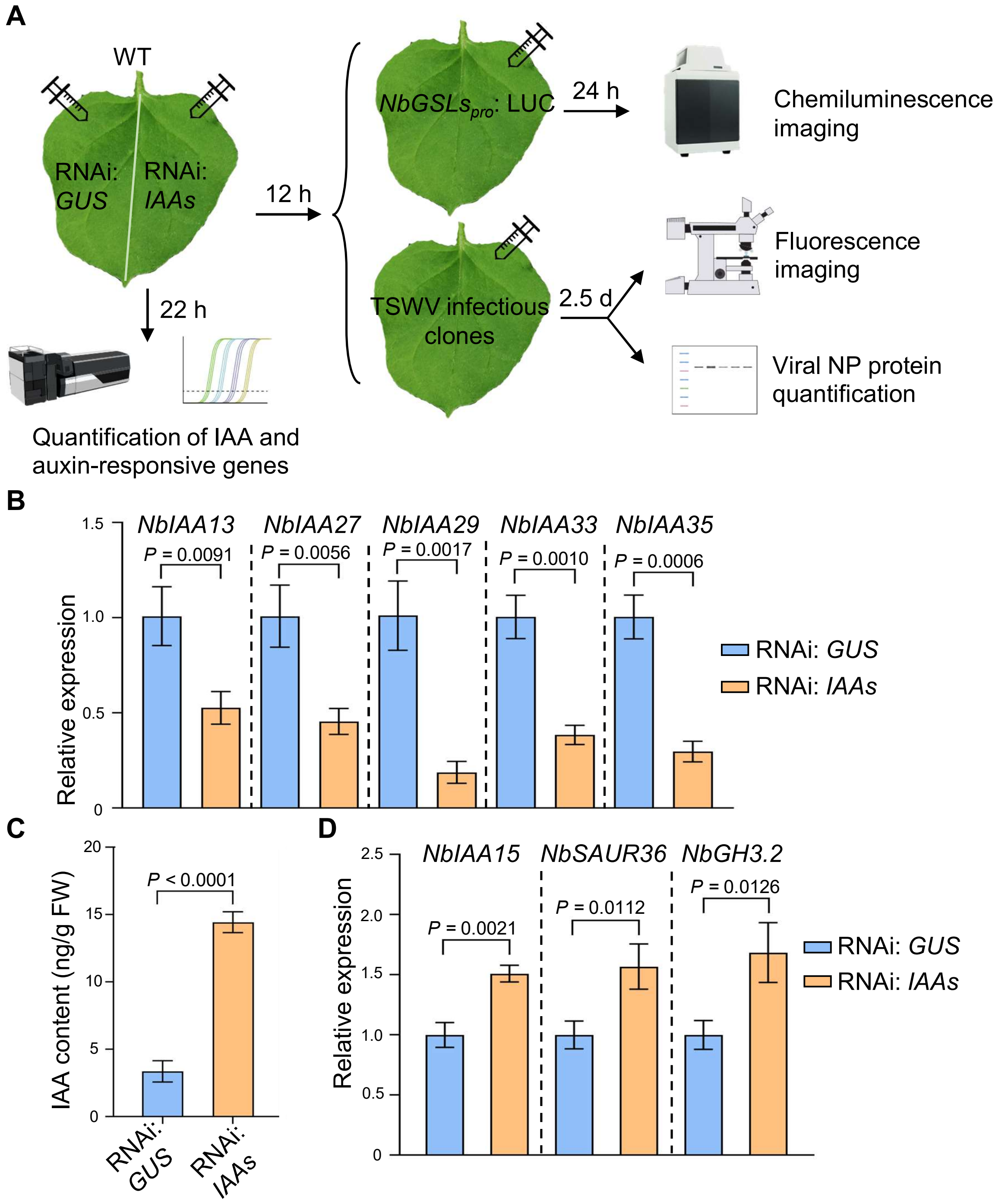

Figure S9. Silencing *NbIAA13/27/29/33/35* enhances the transcriptional activities of *NbGSL5/8* promoter and increases IAA content.

(**A**) Schematic diagrams of dsRNA-based gene silencing of *NbIAA13/27/29/33/35* (*NbIAAs*) using a half-leaf method in *N. benthamiana* leaves and the flowchart showing experiments performed in Fig. 3 and fig. S9. (**B**) Relative expression of *NbIAA13/27/29/33/35* in RNAi: *GUS*- or RNAi: *IAAs*-treated *N. benthamiana* plant leaves. (**C**) Quantification of IAA content in *IAAs*-silenced or non-silenced leaf samples by LC-MS/MS. (**D**) qRT-PCR analysis of the expression levels of auxin-responsive genes in RNAi: *GUS* or RNAi: *IAAs* leaf samples. Values in (B to D) represent means ± SD of three biologically independent replicates, and statistical analysis was performed by two-tailed Student's *t*-test.

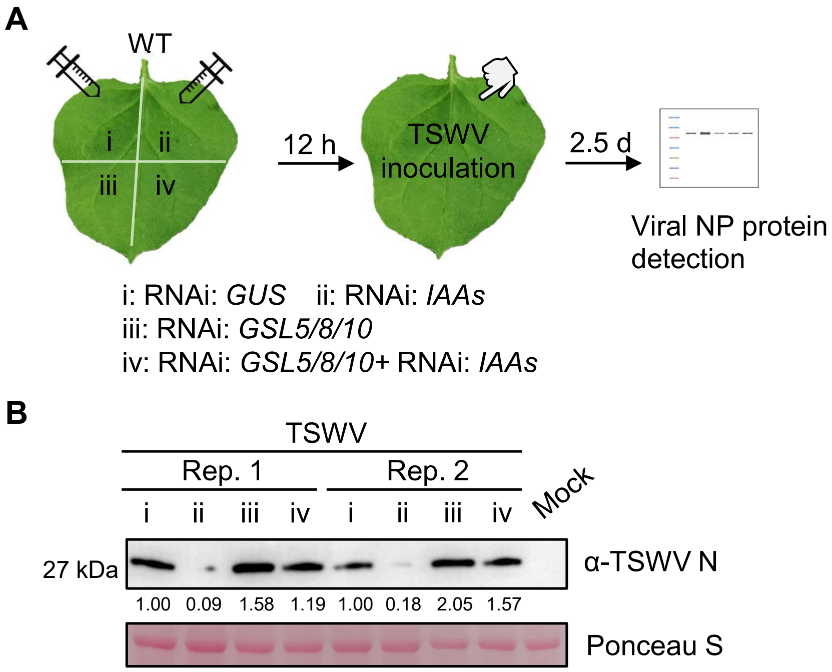

Figure S10. The inhibitory of silencing *NbIAAs* on TSWV infection partially relies on GSL5/8/10.

(**A**) Schematic diagrams of dsRNA-based gene silencing of *NbIAA13/27/29/33/35* (*NbIAAs*) using a half-leaf method in *N. benthamiana* leaves and the flowchart showing experiments performed in fig. S10. (**B**) TSWV accumulation in leaf samples from panel (A) by western blot. Ponceau-stained bands shows protein loading. Protein levels were determined using ImageJ software.

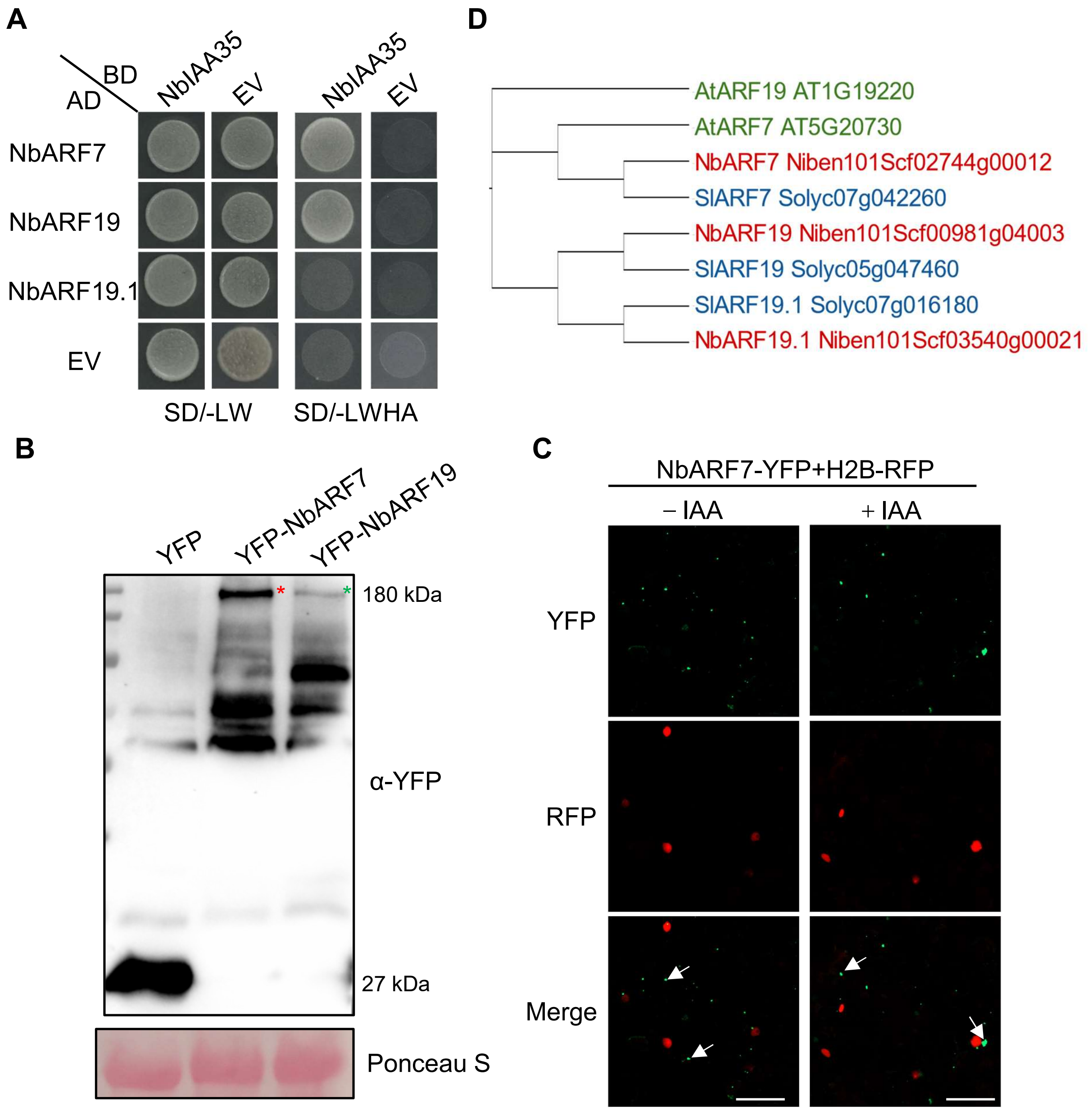

Figure S11. Analysis of NbARF7 and its homolog NbARF19.

(**A**) Y2H analysis for the interaction of NbIAA35 with NbARF7, NbARF19, or NbARF19.1. Yeast cells were grown on medium SD/-LW and selected on medium SD/-LWHA at 30°C for 4 days. The experiment was repeated three times with similar results. (**B**) The protein expression of YFP-NbARF7 and YFP-NbARF19 by immunoblotting. YFP-tagged constructs were expressed in *N. benthamiana* leaves for 22 hpi. The red asterisk indicated YFP-NbARF7 and the green indicated YFP-NbARF19. Ponceau-stained bands shows protein loading. (**C**) IAA treatment does not alter the cytoplasmic localization of NbARF7. Confocal images of NbARF7-YFP in *N. benthamiana* leaves treated with or without IAA. H2B-RFP was used as the nuclear marker. Scale bars, 20 µm. (**D**) Phylogenetic analysis of ARF7/19/19.1 in *A. thaliana*, *S. lycopersicum,* and *N. benthamiana*. The tree was constructed in MAGA7 software using a neighbor-joining method (Poisson model, bootstrap = 1000).

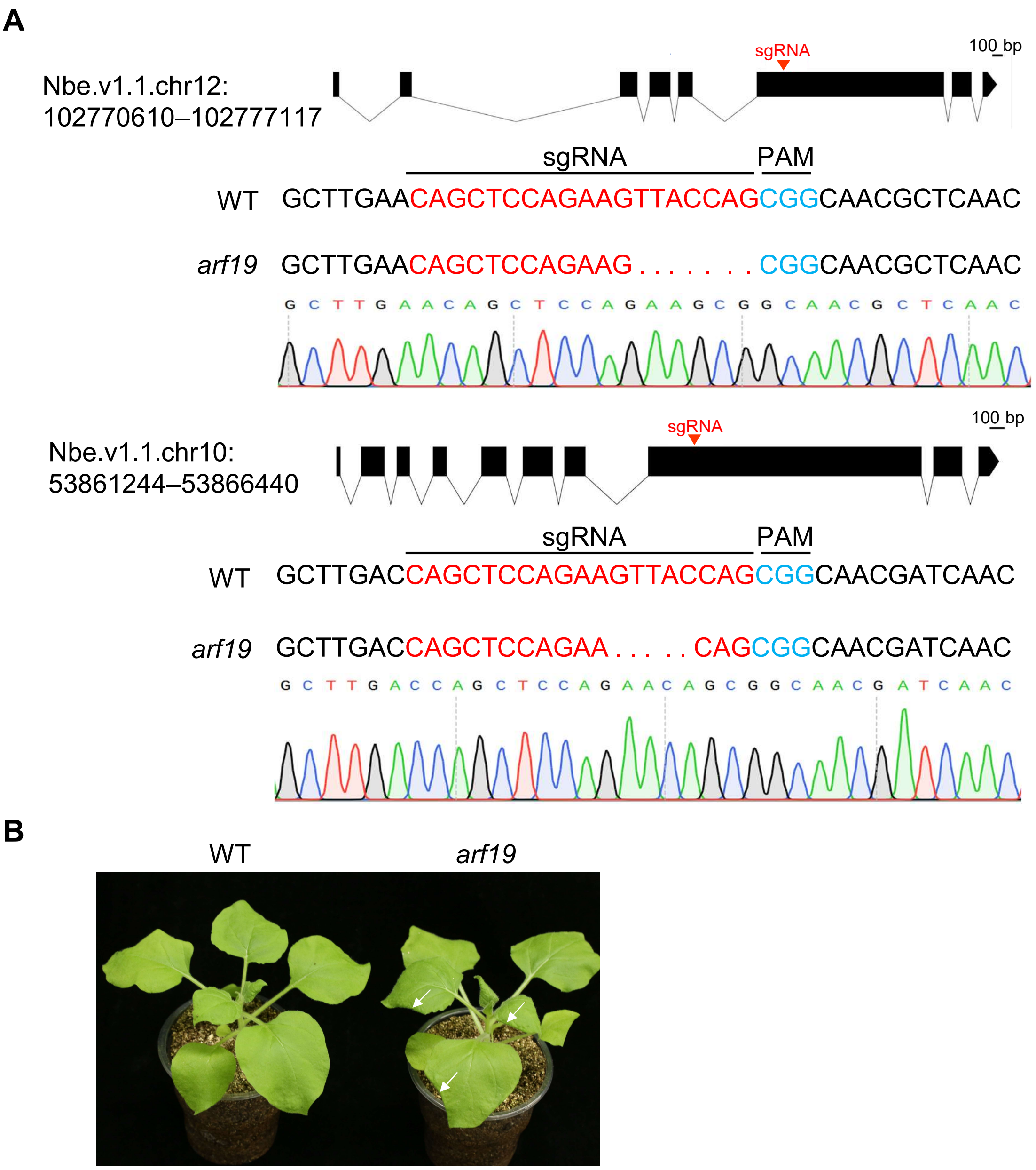

Figure S12. Generation of *Nbarf19* knockout mutant plants by CRISPR/Cas9.

(**A**) Schematic diagram showing the genome structure of *NbARF19*. The black boxes represent the exons and the black lines represent the introns. The sequence of sgRNA and PAM motif are showing in red and blue, respectively. Sequence alignment between WT and *arf19* mutant revealed a 7-bp and 5-bp deletion of two *NbARF19* copies (Nbe.v1.1.chr12: 102770610–102777117 and Nbe.v1.1.chr10: 53861244–53866440), respectively. (**B**) Phenotype of WT and *arf19* mutant *N. benthamiana* plants. Photos were taken at 5 weeks after planting.

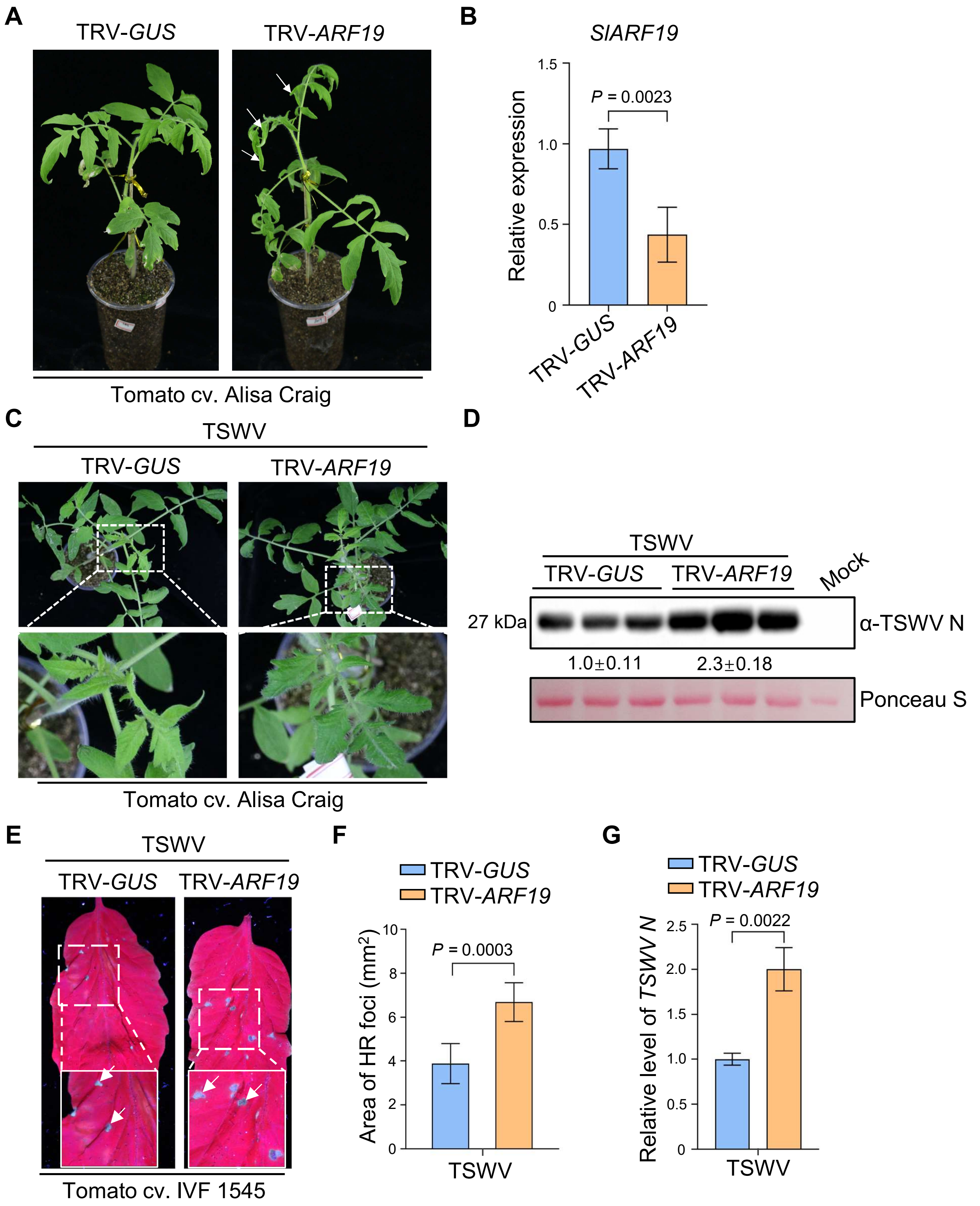

Figure S13. Silencing of *SlARF19* facilitates TSWV infection in tomato plants.

(**A**) Phenotype of TRV-*GUS* and TRV-*ARF19* tomato plants (lacking *Sw-5b*). Photos were taken at 30 dpi. (**B**) qRT-PCR analysis of *SlARF19* expression in *GUS*- or *SlARF19*-silenced plants. Values are means ± SD with three biological independent treatments. (**C**) Symptoms of *SlARF19*-silenced or non-silenced plants inoculated with TSWV. Photos were taken at 12 dpi. (**D**) Immunoblotting analysis of TSWV N accumulation in systemic leaves from panel (C). Ponceau-stained bands shows protein loading. Protein levels were determined using ImageJ software. (**E**) HR foci in *GUS*- or *SlARF19*-silenced tomato (containing *Sw-5b*) leaves

inoculated with TSWV. Photos were taken at 5 dpi under an UV light. (**F**) Quantification of mean areas of HR foci in leaf samples from panel (E). Values are means ± SD, n = 6 biologically independent leaf samples. (**G**) qRT-PCR analysis of TSWV *N* accumulation in panel (E). Values represent means ± SD of three biologically independent treatments. Statistical analysis in (B, F, and G) was performed by two-tailed Student's *t*-test.

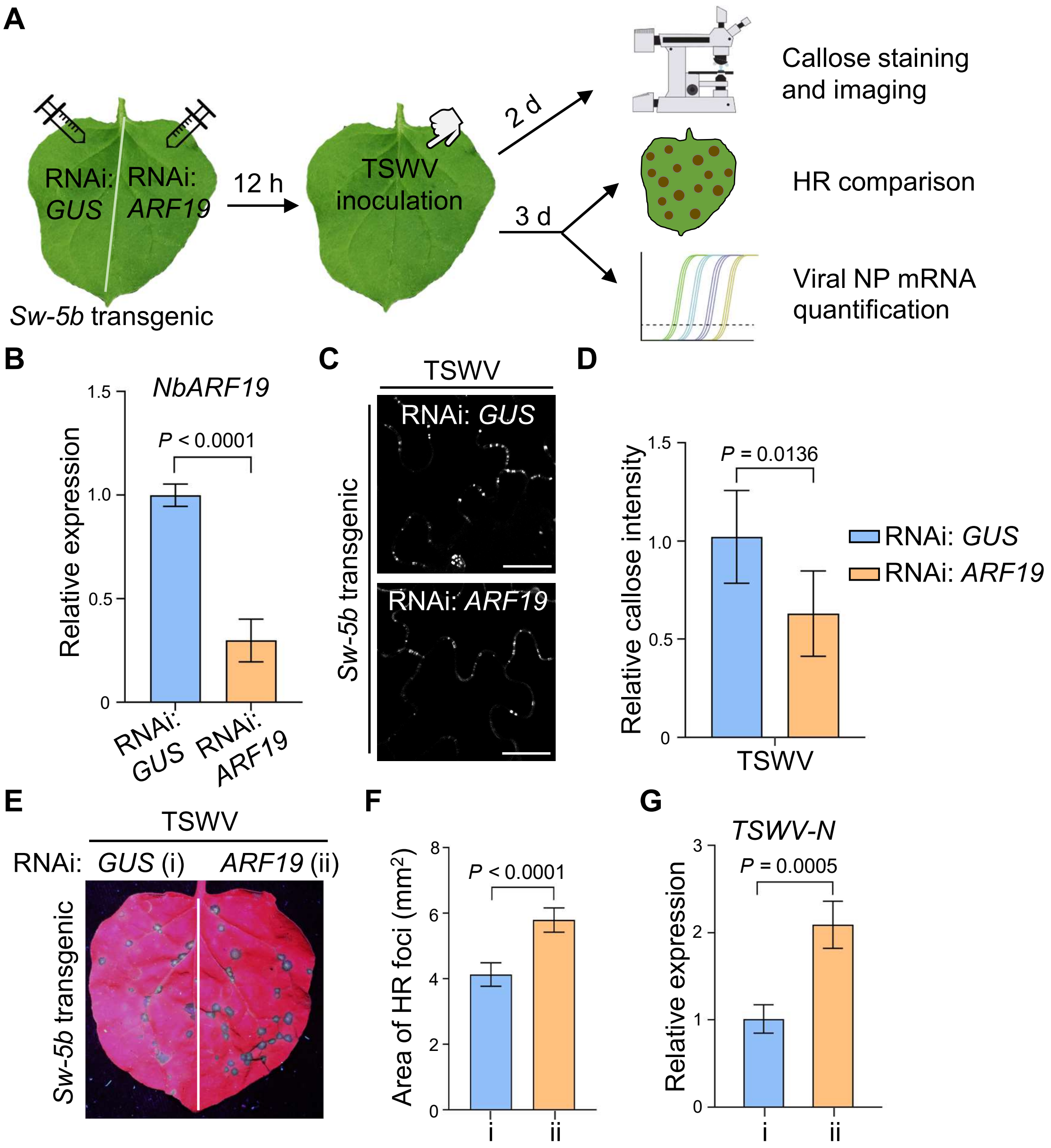

Figure S14. Knockdown of *NbARF19* promotes TSWV local spread in *Sw-5b*-transgenic *N. benthamiana* plants.

(**A**) Schematic diagrams of dsRNA-based gene silencing of *NbARF19* using a half-leaf method in *N. benthamiana* leaves and the flowchart showing experiments performed in fig. S14. (**B**) The expression level of *NbARF19* in leaves treated with RNAi construct by qRT-PCR. Values are means ± SD of three biological replicates. (**C**) Callose fluorescence in *NbARF19*-silenced or non-silenced *Sw-5b*-transgenic plant leaves inoculated with TSWV. Leaf samples were collected at 2 dpi. Scale bars, 20 μm. (**D**) Quantification of the callose intensity in panel (C). Values are means ± SD, n = 6 biologically independent samples. (**E**) HR foci in *ARF19*- or *GUS*-silenced half-leaves from *Sw-5b*-transgenic plants inoculated with TSWV. Photos were taken at 3 dpi under an UV light. (**F**) Quantification of mean areas of HR foci in panel (E). Values are means ± SD, n = 6 biologically independent leaf samples. (**G**) Relative expression level of TSWV *N* in panel (E) by qRT-PCR. Values are means ± SD, n = 3 biologically independent replicates. Statistical analysis in (B, D, F, and G) was performed by two-tailed Student's t-test.

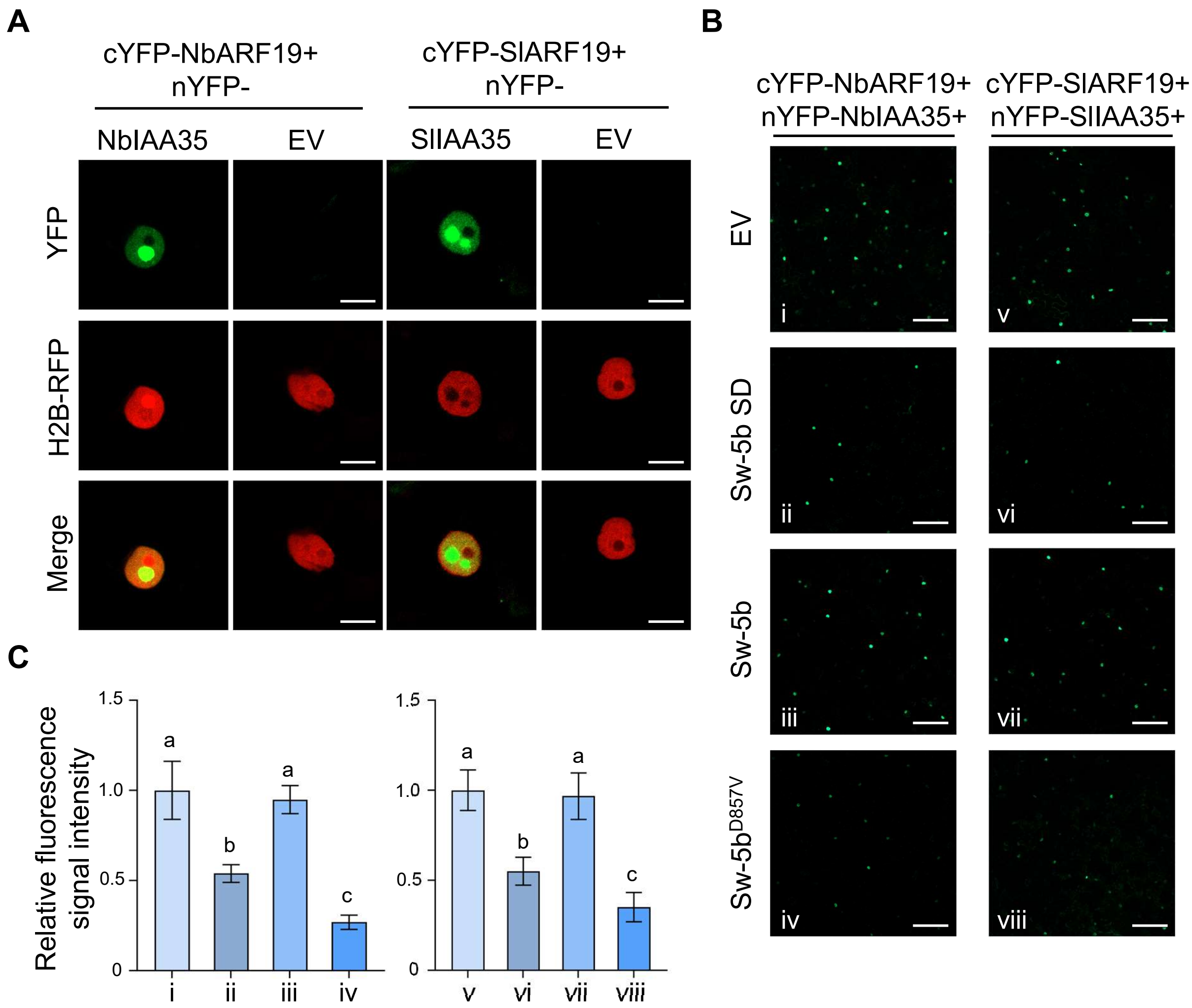

Figure S15. Sw-5b auto-active mutant and SD domain interfere with the interaction between ARF19 and IAA35 *in vivo*.

(**A**) BiFc analysis of the interaction between ARF19 and IAA35. Scale bars, 10 µm. H2B-RFP was served as the nuclear marker. The experiments were repeated three times with similar results. (**B**) The effect of SD, Sw-5b, or Sw-5b^{D857V} on ARF19-IAA35 interaction by BiFc assay. Scale bars, 100 µm. 25 µM MG132 was applied at 14 hpi to prevent protein degradation, and images were taken at 22 hpi under a confocal microscopy. (**C**) Relative fluorescence signal intensity in panel (B). Values are means ± SD (one-way ANOVA, *n* = 3 biologically independent samples). Different letters represent significant differences.

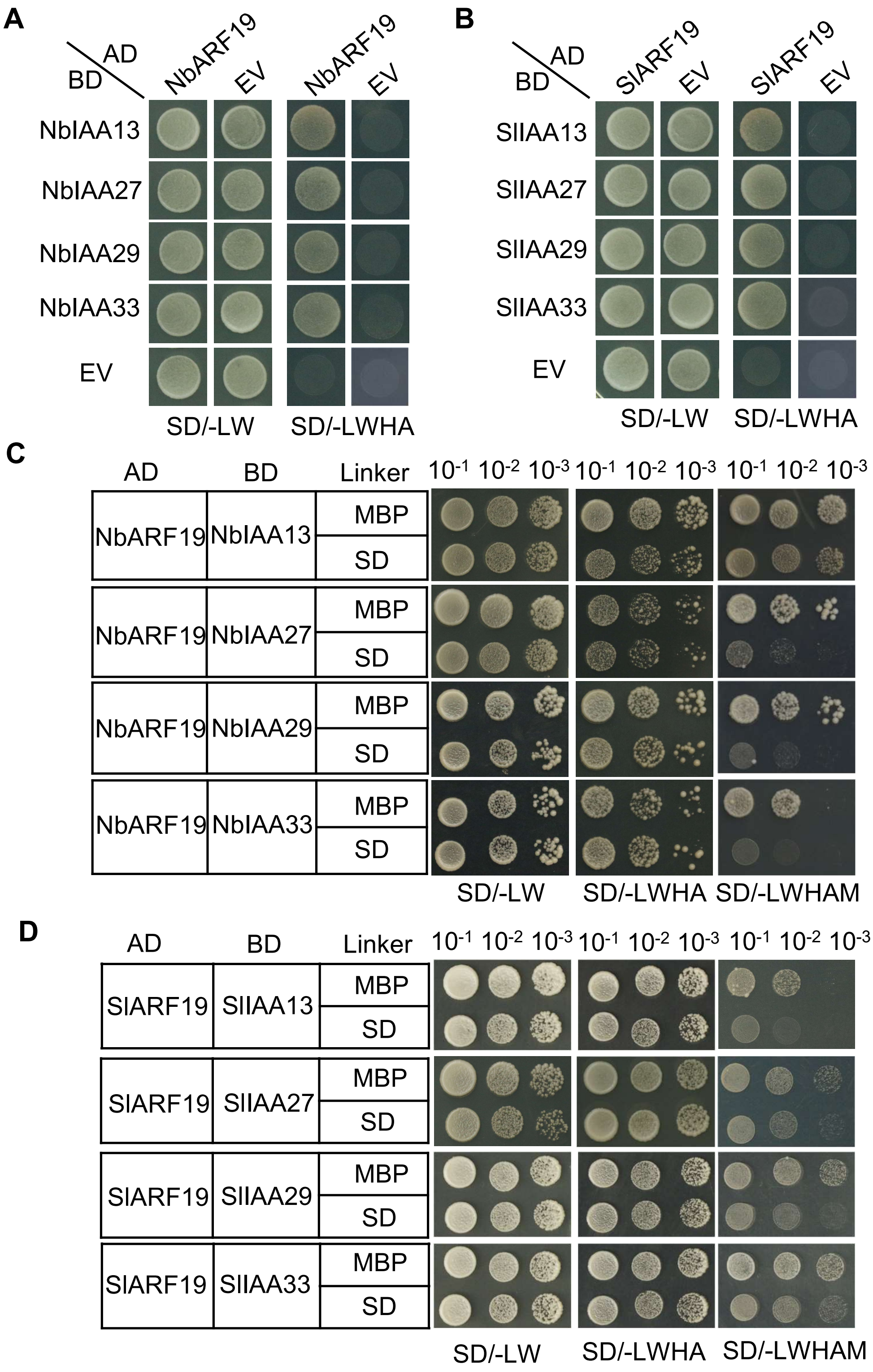

Figure S16. Sw-5b SD interferes with the interaction between ARF19 and IAA13/27/29/33. (**A** and **B**) Y2H analysis of the interaction between NbARF19 and NbIAA13/27/29/33 (A) or between SlARF19 and SlIAA13/27/29/33 (B). Yeast cells were grown on medium SD/-LW and selected on medium SD/-LWHA at 30°C for 4 days. (**C** and **D**) Y3H analysis showing the effect of Sw-5b SD on the interaction between NbARF19 and NbIAA13/27/29/33 (C) or between SlARF19 and SlIAA13/27/29/33 (D). Yeast cells were grown on medium SD/-LW and selected on medium SD/-LWHAM at 30°C for 4 days.

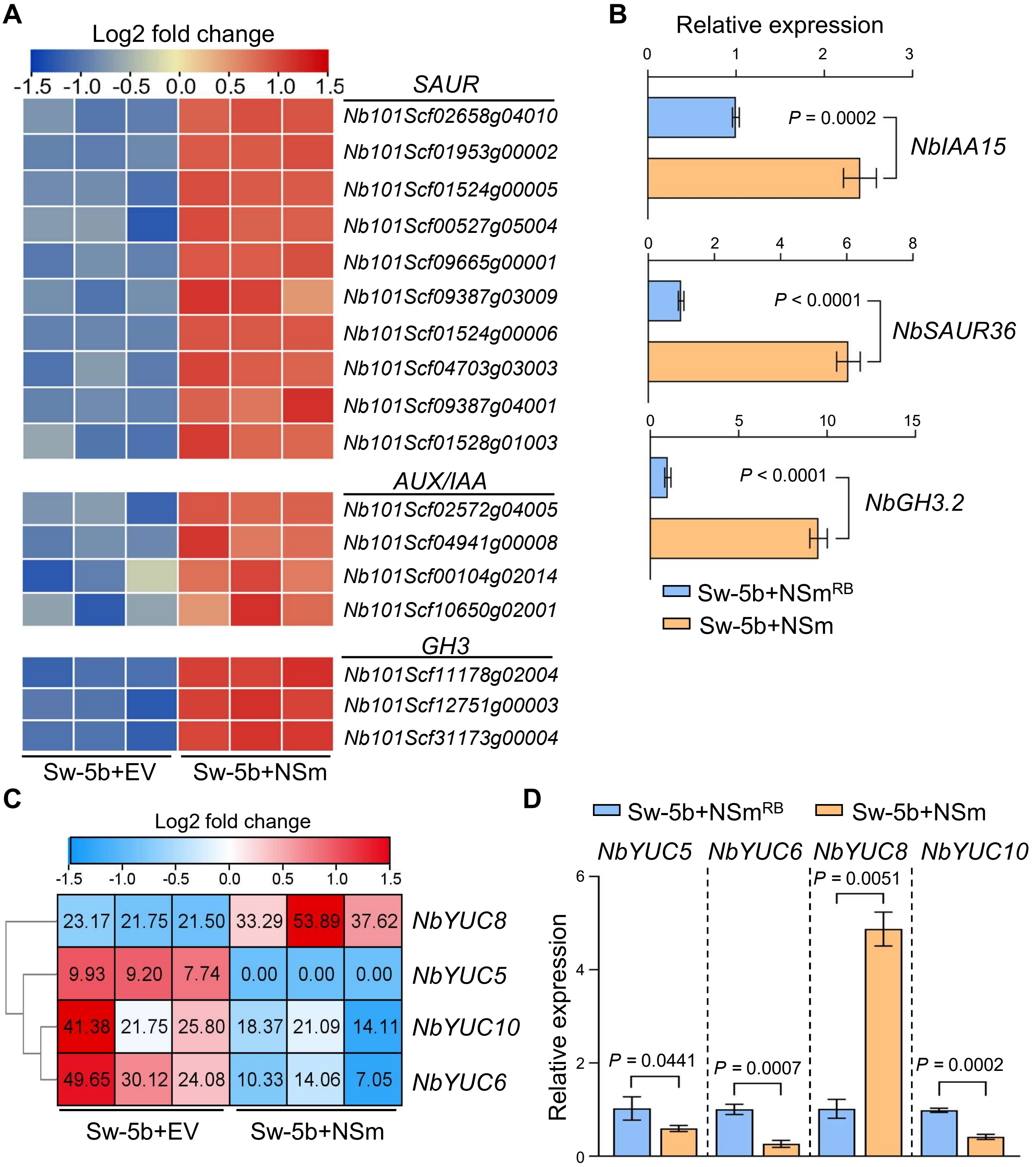

Figure S17. Sw-5b activates auxin response and biosynthesis signaling.

(**A**) Heatmap of upregulated auxin-responsive genes from the RNA-seq data using Sw-5b and NSm co-expressed *N. benthamiana* leaf samples. (**B**) Relative expression levels of auxin-responsive genes in *N. benthamiana* leaves co-expressing Sw-5b and NSm or NSm^{RB} by qRT-PCR. (**C**) Heatmap of differential expressed *NbYUC* genes from the RNA-seq data using Sw-5b and NSm co-expressed *N. benthamiana* leaf samples. The heatmap in (A and C) was drew using TBtools software, and values presented in panel (C) represent FPKM. (**D**) Relative expression levels of *NbYUC5/6/8/10* in leaves expressing Sw-5b with NSm or NSm^{RB} by qRT-PCR. Leaf samples in (B and D) were collected at 22 hpi and values are means ± SD (two-tailed student's *t*-test, *n* = 3 biologically independent replicates).

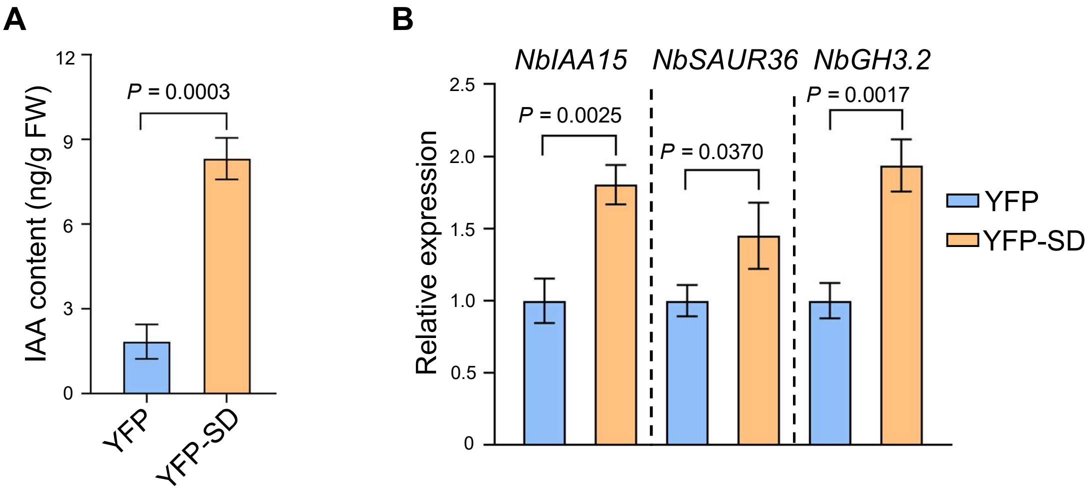

Figure S18. Overexpression of Sw-5b SD activates auxin biosynthesis and response signaling.

(**A**) Quantification of IAA content in leaf samples expressing YFP or YFP-SD by LC-MS/MS. Values represent means ± SD, $n = 3$ biologically independent replicates. (**B**) qRT-PCR analysis of auxin-responsive genes in YFP- or YFP-SD-expressed leaf samples. Values are means ± SD of three biologically independent replicates. Leaf samples in (A and B) were collected at 22 hpi. Statistical analysis in (A and B) was performed by two-tailed Student's *t*-test.

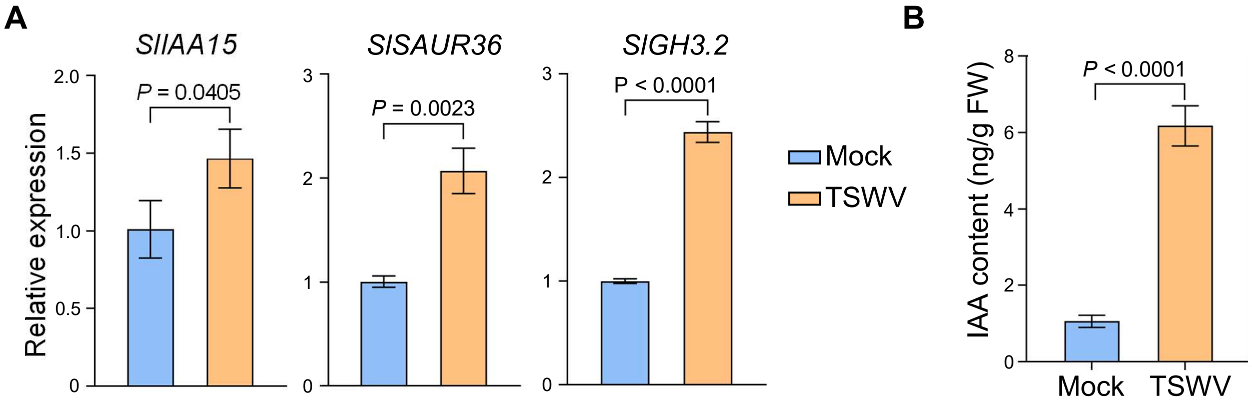

Figure S19. TSWV inoculation activates auxin signaling in tomato cv. 1545 (harboring *Sw-5b*) leaves.

(**A**) Relative expression levels of auxin-responsive genes in tomato leaves inoculated with TSWV by qRT-PCR. Values represent means ± SD of three biological samples. (**B**) Quantification of IAA content in tomato leaves (containing *Sw-5b*) inoculated with TSWV by LC-MS/MS. Leaf samples in (A and B) were collected at 2 dpi, and statistical analysis was performed by two-tailed Student's *t*-test.

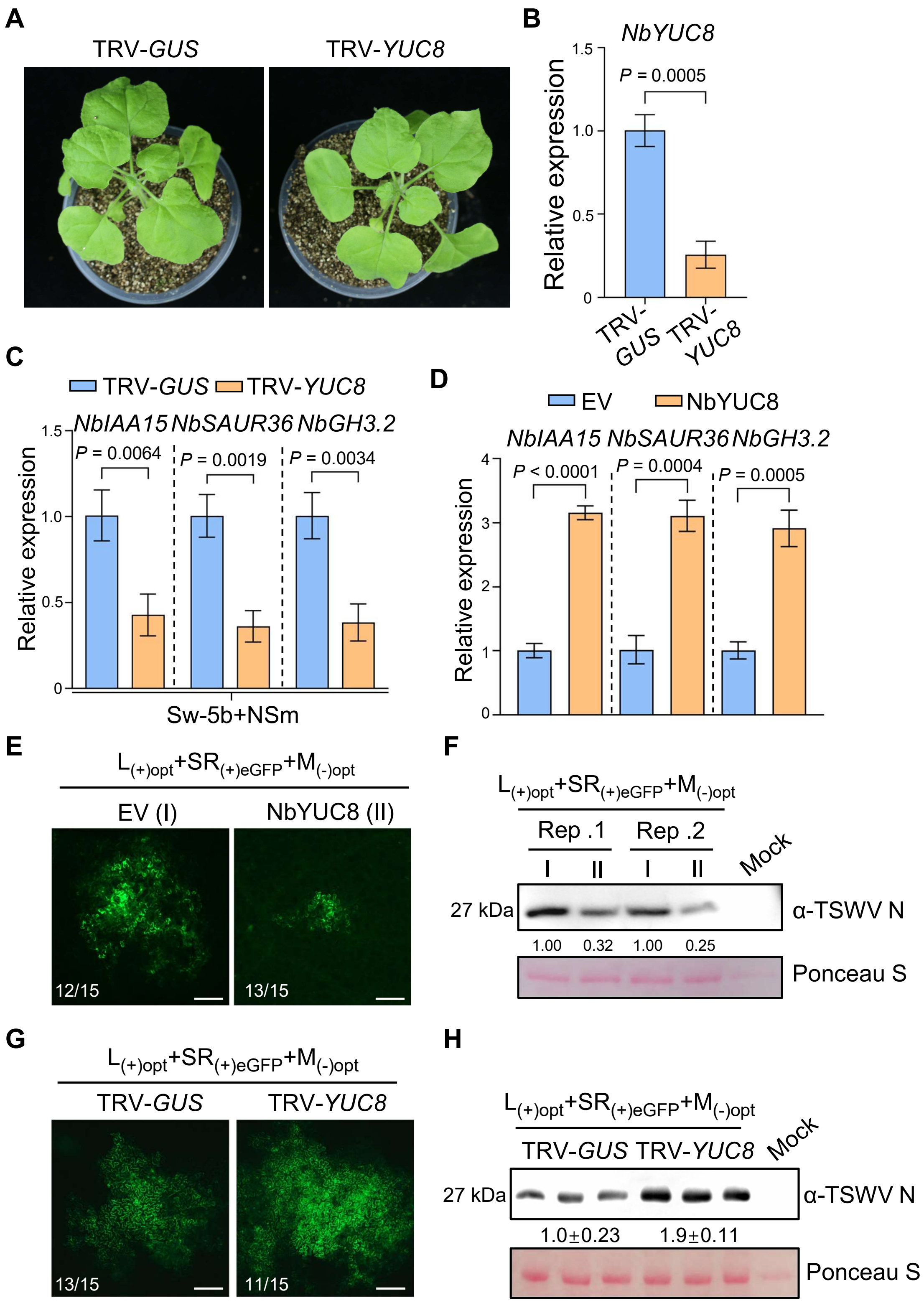

Figure S20. NbYUC8 negatively regulates TSWV infection in *N. benthamiana* plants.

(**A**) Phenotype of TRV-*YUC8* and TRV-*GUS N. benthamiana* plants by TRV-based VIGS. Photos were taken at 21 dpi. (**B**) qRT-PCR analysis of *NbYUC8* expression in *GUS*- or *NbYUC8*-silenced plants. (**C**) Relative expression levels of auxin-responsive genes in *YUC8*- or *GUS*-silenced leaves co-expressing Sw-5b and NSm by qRT-PCR. (**D**) The expression levels of auxin-responsive genes in *N. benthamiana leaves* expressing EV or NbYUC8. Values in (B and C) are means ± SD (two-tailed Student's *t*-test, *n* = 3 biological replicates).

(**E**) Fluorescence images of TSWV infectious clones co-expressed with EV or NbYUC8 in *N. benthamiana* leaves. Scale bars, 200 μm. (**F**) Western blot analysis of TSWV N in leaf samples from panel (E) using N specific antibodies. (**G**) Fluorescence images of TSWV infectious clones expressed in *GUS*- or *NbYUC8*-silenced plants. Scale bars, 200 μm. (**H**) Immunoblotting analysis of TSWV N accumulation in panel (G). Ponceau staining in (F and H) shows protein loading, and protein levels were determined using ImageJ software.

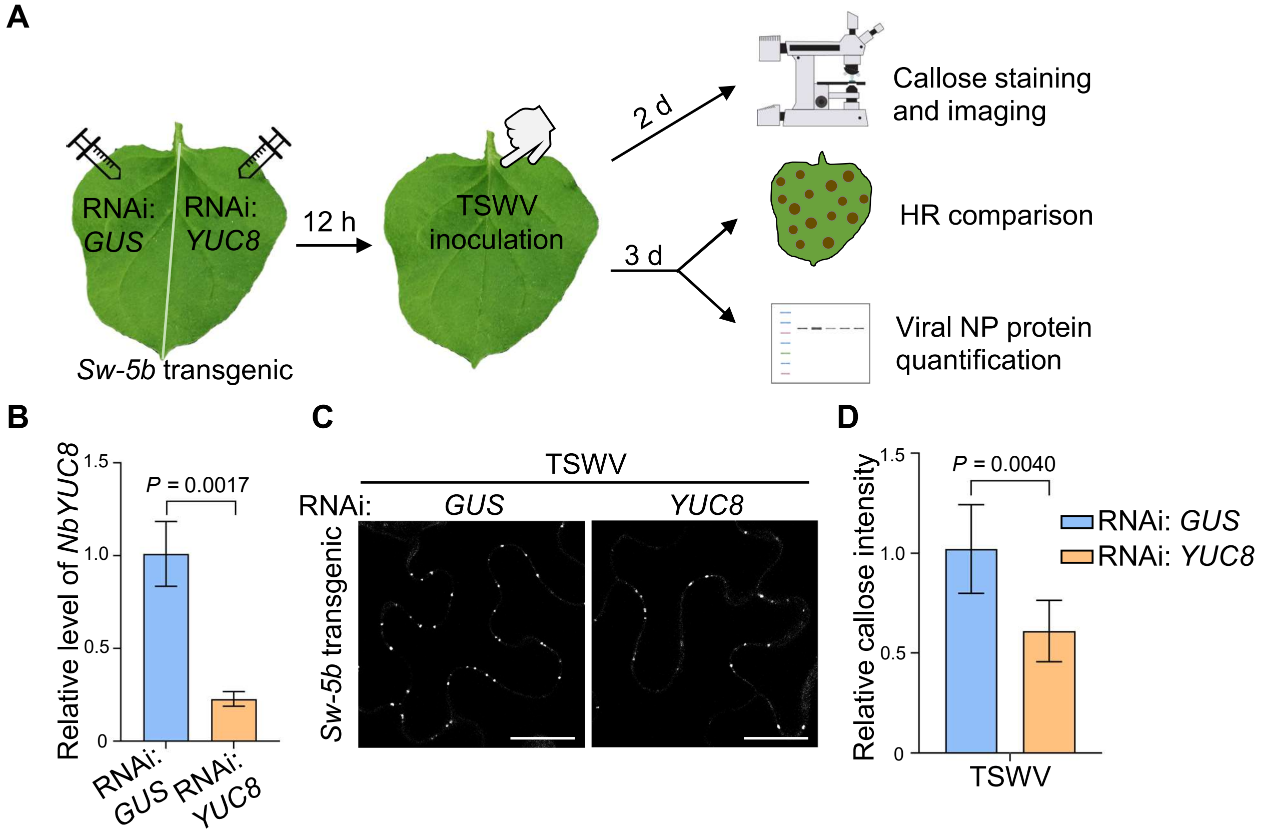

Figure S21. Silencing of *NbYUC8* reduces callose accumulation in *Sw-5b*-transgenic *N. benthamiana* inoculated with TSWV.

(**A**) Schematic diagrams of dsRNA-based gene silencing of *NbYUC8* using a half-leaf method in *N. benthamiana* leaves and the flowchart showing experiments performed in Fig. 6 and fig. S21. (**B**) The expression level of *NbYUC8* in RNAi: *GUS* or RNAi: *YUC8* leaf samples by qRT-PCR. Values are means ± SD, n = 3 biological replicates. (**C**) Callose fluorescence in RNAi: *GUS* or RNAi: *YUC8* leaves inoculated with TSWV. Leaf samples were collected at 2 dpi. Scale bars, 20 µm. (**D**) Quantification of the callose intensity in panel (C). Values are means ± SD of six biologically independent samples. Statistical analysis in (B and D) was performed by two-tailed Student's *t*-test.

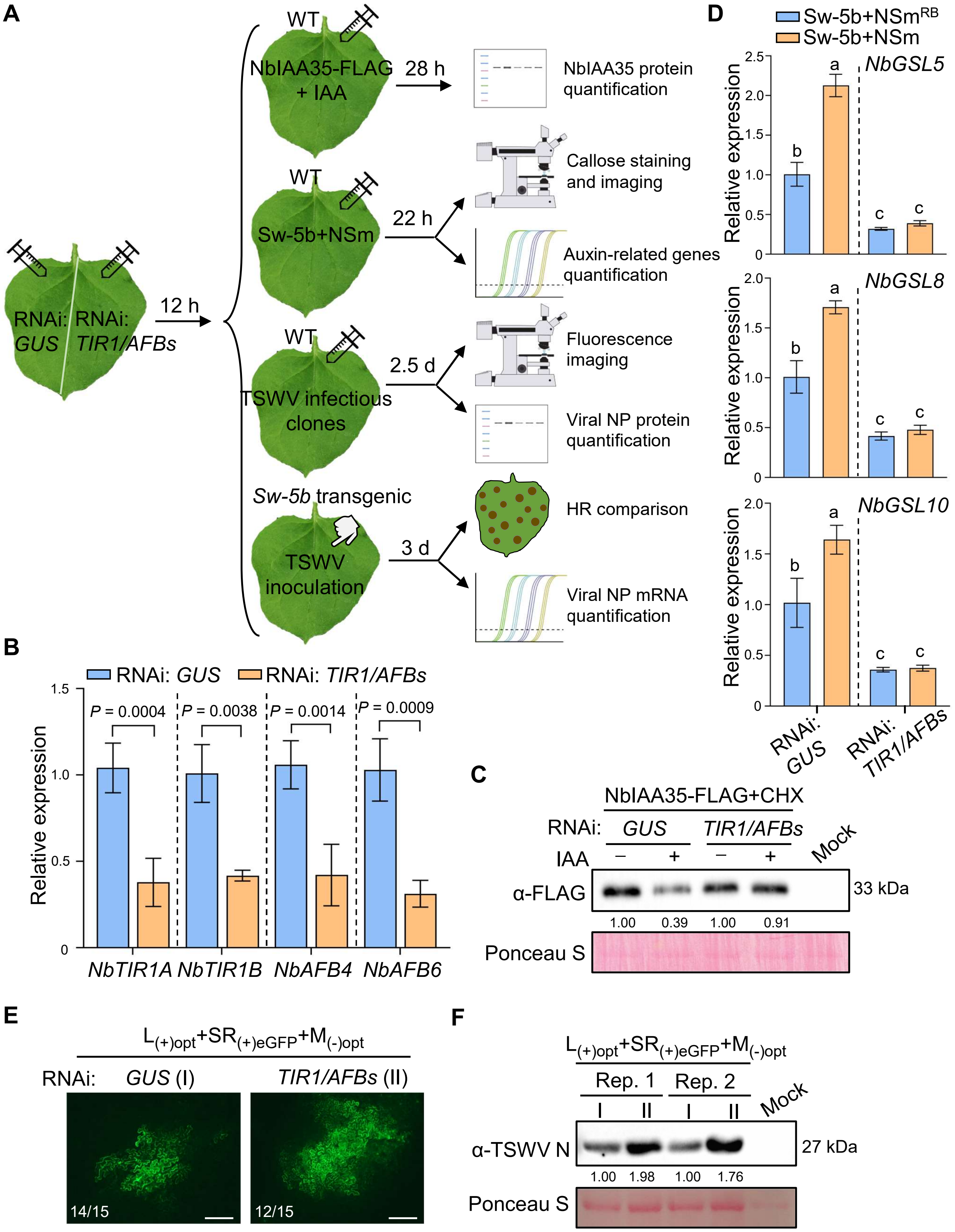

Figure S22. Silencing of auxin receptors *TIR1/AFBs* inhibits *NbGSLs* expression and facilitates TSWV infection in *N. benthamiana.*

(**A**) Schematic diagrams of dsRNA-based gene silencing of *NbTIR1/AFBs* in *N. benthamiana* leaves and the flowchart showing experiments performed in Fig. 7 and fig. S22. (**B**) The expression levels of *NbTIR1A*, *NbTIR1B*, *NbAFB4,* and *NbAFB6* in RNAi: *TIR1/AFBs*-silenced

or non-silenced leaves by qRT-PCR. Values are means ± SD (two-tailed Student's *t*-test, *n* = 3 biological replicates). (**C**) Western blot analysis of NbIAA35-FLAG in RNAi: *GUS* or RNAi: *TIR1/AFBs* leaf samples treated with IAA or water. 25 μM CHX was applied at 22 hpi to block protein production, immediately followed by IAA treatment. Leaf samples were collected at 28 hpi. (**D**) qRT-PCR analysis of *NbGSL5/8/10* expressions in RNAi: *GUS* or RNAi: *TIR1/AFBs* leaf samples co-expressing Sw-5b with NSm or NSm^{RB}. Values are means ± SD (two-way ANOVA, *n* = 3 biological replicates). Different letters represent significant differences. (**E**) Fluorescence images of TSWV infectious clones expressed in *GUS*- or *TIR1/AFBs*-silenced *N. benthamiana* leaves. Scale bars, 400 μm. (**F**) Immunoblotting analysis of TSWV N accumulation in panel (E). Ponceau-stained bands in (C and F) show protein loading and protein levels were determined using ImageJ software.

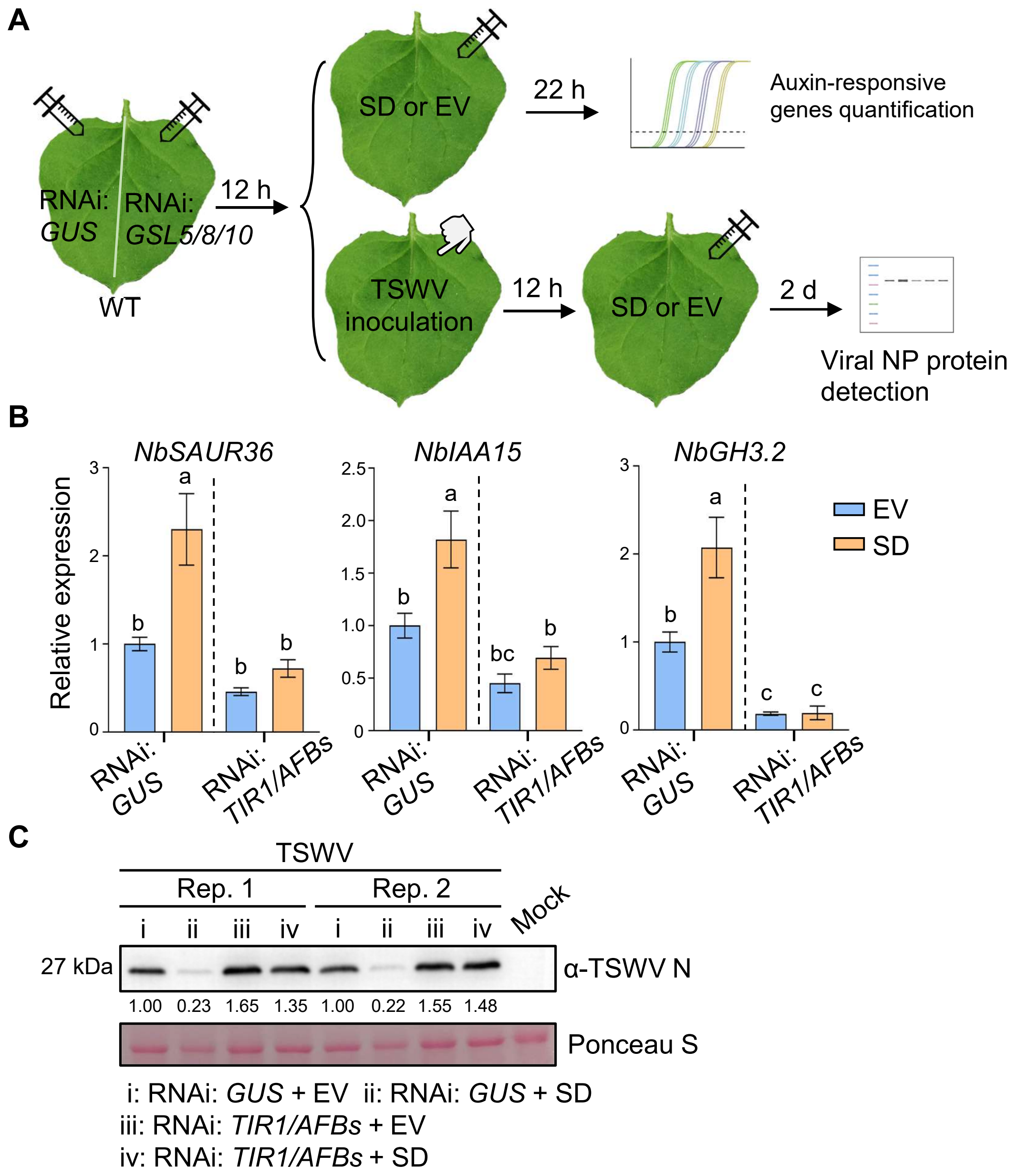

Figure S23. Knockdown of auxin receptors *TIR1/AFBs* impairs SD's ability of activating auxin-responsive genes and inhibiting TSWV infection.

(**A**) Schematic diagrams of RNAi: *TIR1/AFBs* in *N. benthamiana* leaves and the flowchart showing experiments performed in fig. S23. (**B**) The expression levels of *NbSAUR36*, *NbIAA15*, and *NbGH3.2* in *GUS* or *TIR1/AFBs*-silenced leaves expressing EV or SD, respectively. Values are means ± SD, n = 3 biological replicates. Statistical was performed by two-way ANOVA, different letters represent significant differences. (**C**) Immunoblotting analysis of TSWV N accumulation in panel (A). Ponceau staining shows protein loading. Protein levels were determined using ImageJ software.

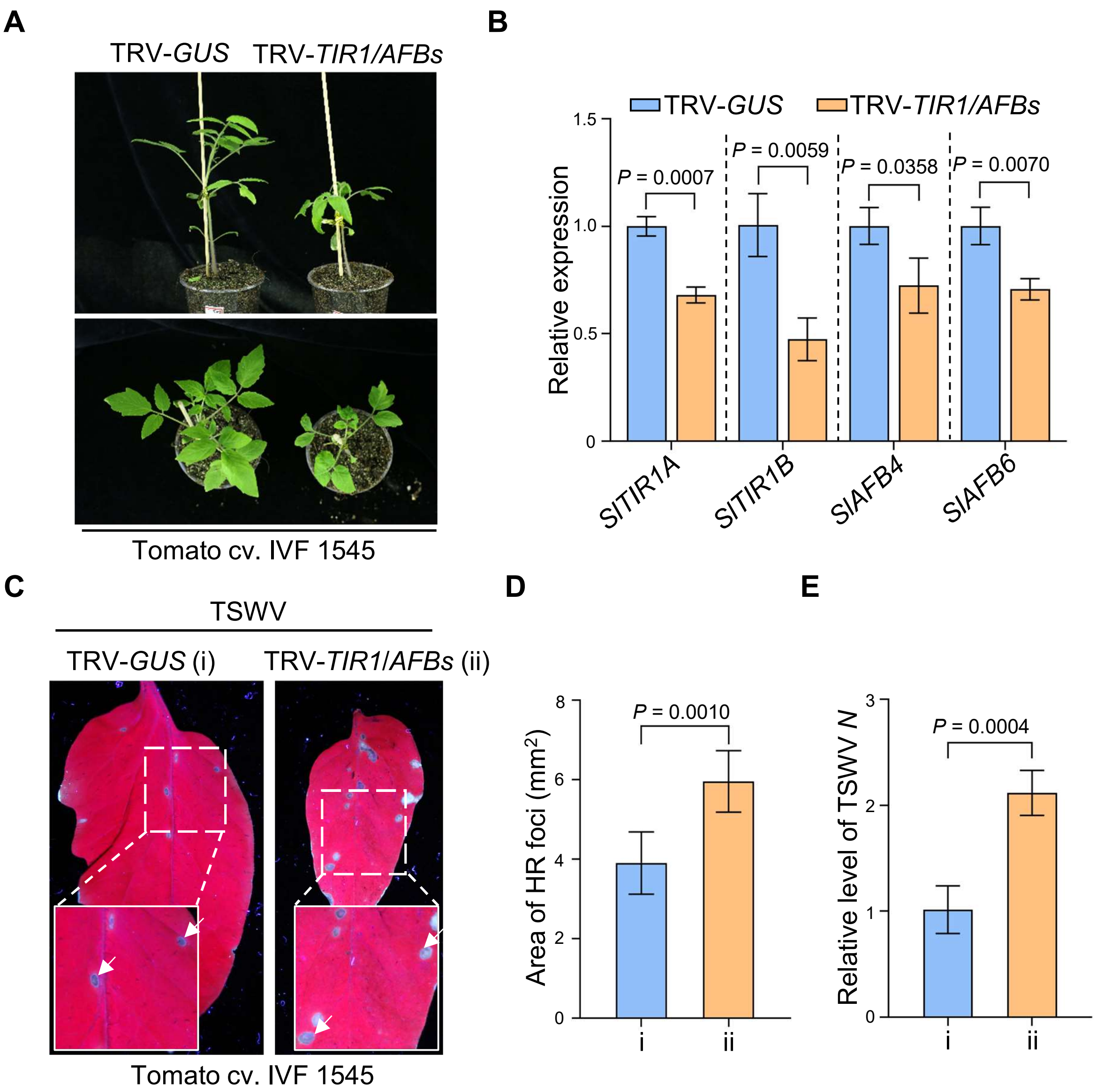

Figure S24. Knockdown of auxin receptors *TIR1/AFBs* promotes TSWV local spread in tomato cv. 1545 (containing *Sw-5b*) leaves.

(**A**) Phenotype of TRV-*GUS* and TRV-*TIR1/AFBs* tomato plants. Photos were taken at 30 dpi. (**B**) qRT-PCR analysis of *SlTIR1A*, *SlTIR1B*, *SlAFB4*, and *SlAFB6* expression levels in TRV-*GUS* or TRV-*TIR1/AFBs* plants. Values are means ± SD, *n* = 3 biological replicates. (**C**) HR foci in *SlTIR1/AFBs*-silenced or non-silenced tomato leaves inoculated with TSWV. Photos were taken at 5 dpi under an UV light. (**D**) Quantification of mean areas of HR foci in leaves from panel (C). Values are means ± SD of six biologically independent samples. (**E**) qRT-PCR analysis of TSWV *N* accumulation in panel (C). Values represent means ± SD of three biologically independent replicates. Statistical analysis in (B, D, and E) was performed by two-tailed Student's *t*-test.

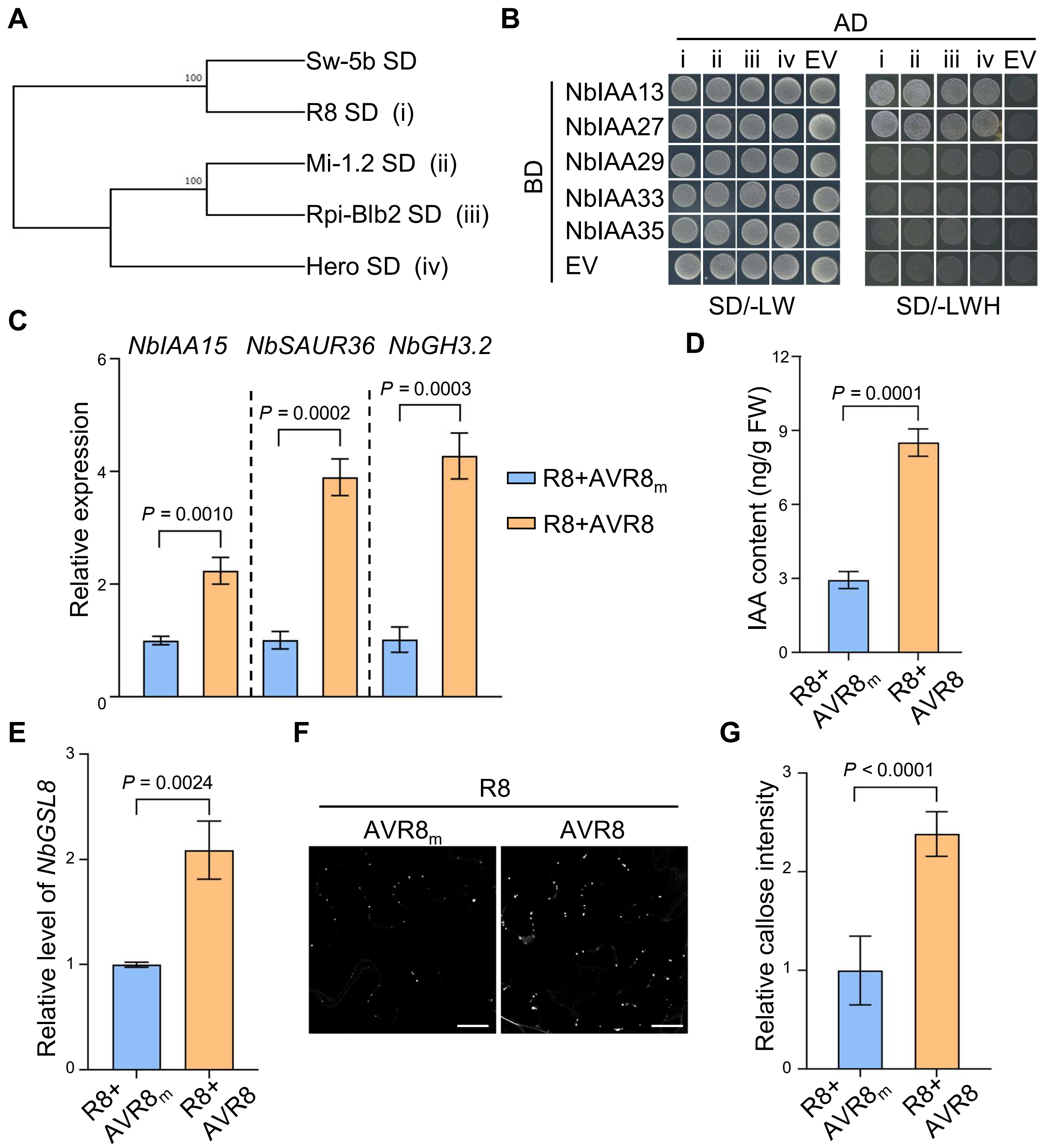

Figure S25. SD-containing NLR R8 activates auxin signaling and callose deposition.
(**A**) Phylogenetic analysis of SDs from *Solanaceae* CNLs. The tree was constructed in MEGA7 using Poisson model with bootstrap values of 1,000 replicates. (**B**) Y2H analysis of the interaction between SDs and NbIAA13/27/29/33/35. Yeast cells were grown on medium SD/-LW and selected on medium SD/-LWH at 30℃ for 4 days. The experiment was repeated three times with similar results. (**C**) Relative expression levels of auxin-responsive genes in *N. benthamiana* leaves co-expressing R8 with AVR8 or AVR8m. (**D**) Quantification of IAA content in *N. benthamiana* leaves co-expressing R8/AVR8 or R8/AVR8m by LC-MS/MS. (**E**) Relative mRNA level of *NbGSL8* in *N. benthamiana* leaves co-expressing R8 with AVR8 or AVR8m by qRT-PCR. In (C to E), Values are means ± SD of three biologically independent replicates. (**F**) Callose fluorescence in *N. benthamiana* leaves co-expressing R8 with AVR8 or AVR8m.

Scale bars, 20 μm. (**G**) Quantification of callose intensity in panel (F). Values are means ± SD of six biologically replicates. Leaf samples in (C to F) were collected at 19 hpi. Statistical analysis in (C, D, E, and G) was performed by two-tailed student's *t*-test.

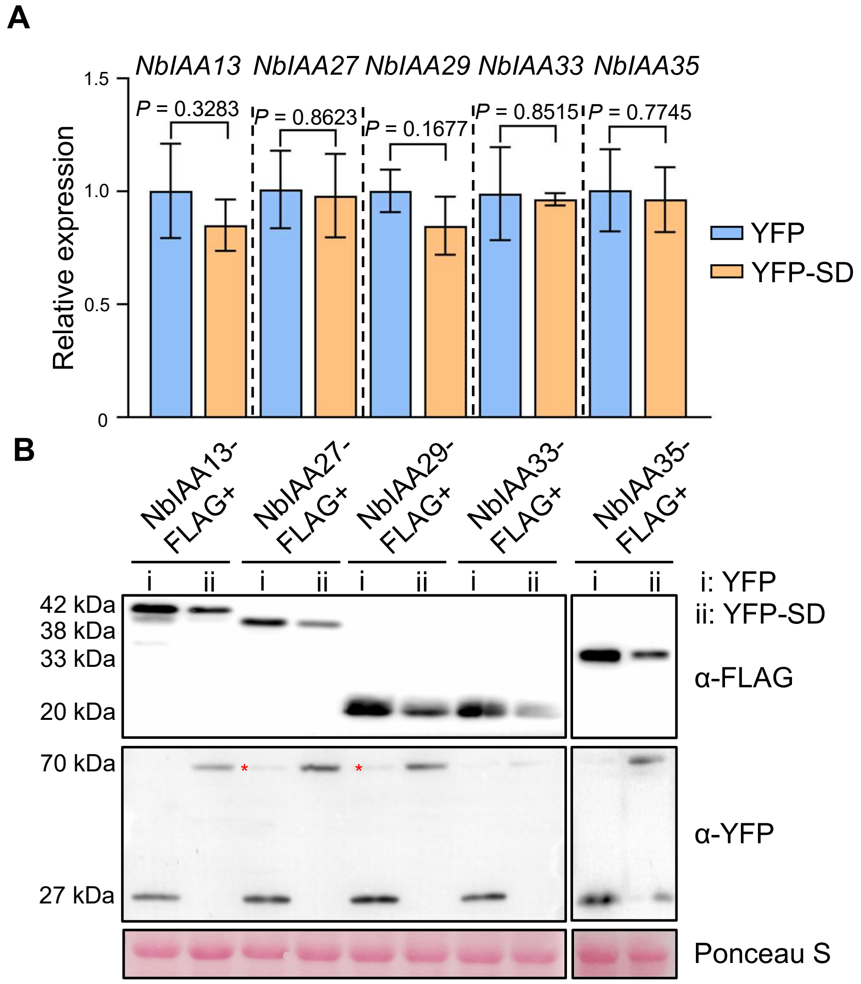

Figure S26. Overexpression of Sw-5b SD decreases the protein stability of IAA13/27/29/33/35.

(**A**) qRT-PCR analysis of the expression levels of *NbIAA13/27/29/33/35* in YFP- or YFP-SD-expressed leaf samples. Values are means ± SD (two-tailed Student's *t*-test, n = 3 biological replicates). (**B**) Western blot analysis of protein levels of NbIAA13/27/29/33/35 in YFP- or YFP-SD-expressed leaf samples. Red asterisks represent non-specific bands. Ponceau staining shows protein loading.

Table S1 (separate file). DEGs in RNA-seq data.
Table S2 (separate file). List of primers used in this study.
